# Unlocking flavin photoacid catalysis through electrophotochemistry†

**DOI:** 10.1039/d4sc03054k

**Published:** 2024-06-18

**Authors:** Samuel Gary, Jack Woolley, Sofia Goia, Steven Bloom

**Affiliations:** a Department of Medicinal Chemistry, University of Kansas Lawrence 66045 USA spbloom@ku.edu; b Department of Physics, University of Warwick Coventry CV4 7AL UK; c Forensic Centre for Digital Scanning and 3D Printing, WMG, University of Warwick Coventry CV4 7AL UK

## Abstract

Molecular flavins are one of the most versatile photocatalysts. They can coordinate single and multiple electron transfer processes, gift hydrogen atoms, form reversible covalent linkages that support group transfer mechanisms, and impart photonic energy to ground state molecules, priming them for downstream reactions. But one mechanism that has not featured extensively is the ability of flavins to act as photoacids. Herein, we disclose our proof-of-concept studies showing that electrophotochemistry can transform fully oxidized flavin quinones to super-oxidized flavinium photoacids that successfully guide proton-transfer and deliver acid-catalyzed products. We also show that these species can adopt a second mechanism wherein they react with water to release hydroxyl radicals that facilitate hydrogen-atom abstraction and sp^3^C–H functionalization protocols. Together, this unprecedented bimodal reactivity enables electro-generated flavinium salts to affect synthetic chemistries previously unknown to flavins, greatly expanding their versatility as catalysts.

## Abstract 2

One untapped reactivity mode of flavins is excited-state proton transfer. We show that electrophotochemistry enables flavins to achieve this elusive reactivity, in addition to C–H functionalization protocols, expanding their mechanistic repertoire.

## Graphical abstract

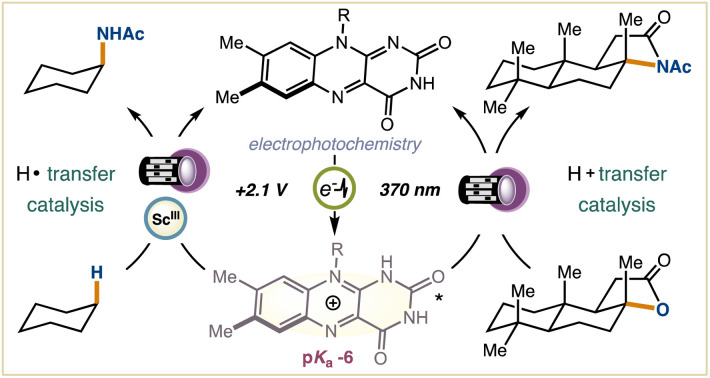

## Introduction

**Fig. 1:**
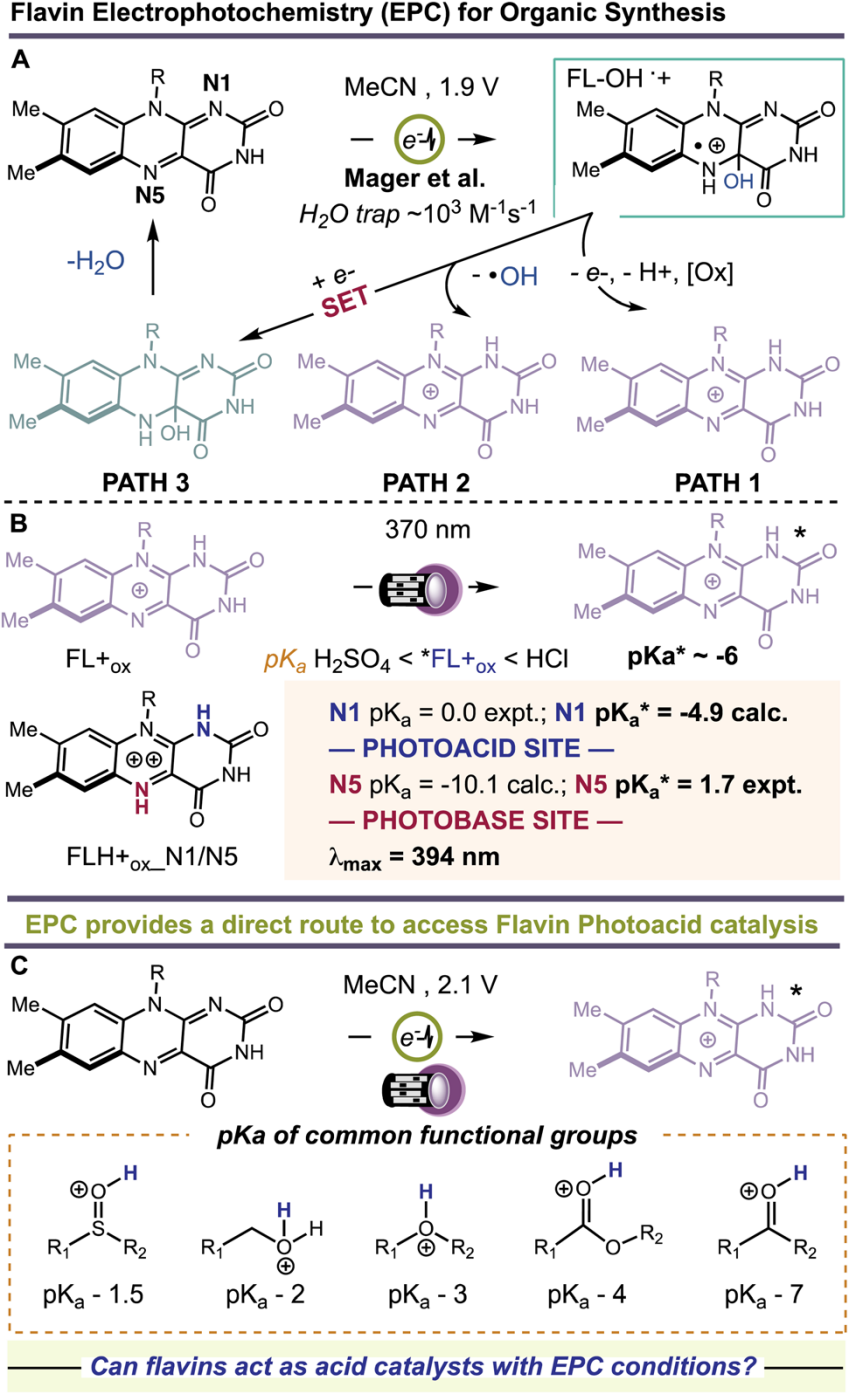
(A) Electrochemical generation of 4a-hydroxy flavins and decomposition to flavinium salts. (B) Photoacid and photobase behavior of flaviniums. (C) Electrophotochemistry route to flavin photoacid catalysis.

Flavins are Nature's multi-tools, choreographing a myriad of chemical reactions. Flavins catalyze single-^1–3^ and multiple-electron transfer processes,^4,5^ group transfer chemistries,^6^ and other redox fluctuations^7^ that orchestrate complex bimolecular synthesis and cellular crosstalk. Flavins also have rich photochemistry.^1^ Depending on their redox state—quinone, semiquinone, or hydroquinone—flavins serve as powerful photo-oxidants and -reductants and can even confer their newly acquired photonic energy to ground state molecules, activating them for high-barrier transformations.^8,9^ Many of these pathways have been successfully leveraged for modern organic synthesis with visible light.^10–12^ A less explored pathway involves the use of flavins as photoacids. Protonation of the flavin quinone affords a flavinium salt, FL+_ox_. In the excited state (*λ*_max_ = 394 nm), these species become ‘strong’ acids. Rarely do organic photoacids exceed p*K*_a_ < 0.^13–18^ Schulman determined a 
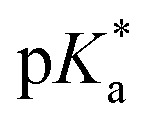
 for protonated riboflavin (RF+_ox_) of −6.2.^19^ Liu *et al.* theorized that bis-protonation of the flavin quinone gives rise to an intermediate, FLH+_ox__N1/N5, with even greater acid-like character in the ground state, p*K*_a_ values of 0 and −10.1 at N1 and N5, respectively.^20^ Photoexcitation increases the acidity of N1 to −4.9 and shifts the basicity of N5 to +1.7, Fig. 1. Unlocking the unique photoacid reactivity of FL+_ox_ has been a significant challenge. In the excited state, FL+_ox_ is a strong oxidant, and promotes competitive single-electron transfer (SET) and hydrogen-atom transfer (HAT) mechanisms.^21–26^ Strong acids are typically used to generate FL+_ox_ from their precursor quinones but cause competing background reactions with many acid-sensitive substrates.^27^ Mager *et al.* showed that flavin quinones are rapidly converted to FL+_ox_ by anodic oxidation at +1.9 V *vs.* SCE in MeCN solvent, Fig. 1.^28^ The advantage of this electrochemical approach is that many unproductive free radicals are eliminated by reaction at the cathode or anode, suppressing background SET and HAT processes. It also precludes the use of strong acids as additives. Hence, the combination of electrochemistry and photochemistry, a.k.a., electrophotochemistry (EPC), could offer a superior route to access FL+_ox_, enabling organic chemists to probe its photoacid reactivity for the first time, Fig. 1. These studies would further complement previous work showing that the combination of electricity and flavins can entice new reaction discovery^29^ and that intermediates generated through EPC can facilitate synthetic disconnections and mechanisms not possible with prior art.^30–34^

## Results and discussion

**Fig. 2:**
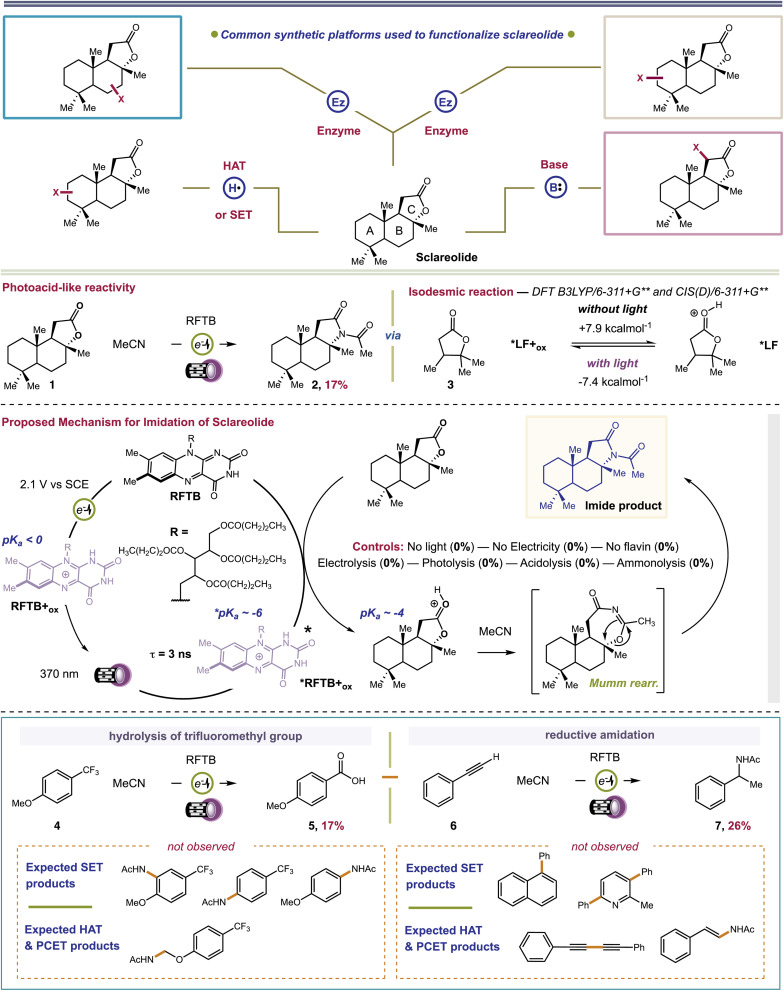
Top panel: Established platforms and regioselectivities observed for functionalization of sclareolide. Second panel: Observed imidation of sclareolide under flavin photoelectrocatalysis conditions and isodesmic calculations for excited-state proton transfer using a computational surrogate for sclareolide. Third panel: Proposed mechanism for formation of imido-sclareolide 2. Bottom panel: Substrates used to distinguish photoacid reactivity from alternate mechanisms.

Sclareolide (1) is an ideal substrate to probe the competency of *FL+_ox_ as a photoacid. Functionalization of sclareolide has been demonstrated with a swathe of chemical platforms that include HAT,^35–37^ SET,^38^ enzymatic,^39–41^ base-mediated,^42,43^ and transition metal-catalyzed processes,^44,45^Fig. 2. Most of these technologies replace a single C–H bond with a new functional group. No extent method directly replaces the key skeletal atoms of sclareolide, *i.e.*, carbon and oxygen, with new atomic structures; although some methods do facilitate O → N ‘swapping’ by first opening the ‘C’ ring lactone to a hydroxamate or dioxazolone, and then recyclizing the ring.^46,47^ A more straightforward path could be achieved through photoacid catalysis. The carbonyl oxygen of the ‘C’ ring lactone can be protonated with a suitable acid, p*K*_a_ CH_3_(C=OH^+^)OMe = −3.9.^48^ Protonation induces C–O bond heterolysis and 3° carbocation formation. The carbocation can be trapped with a suitable nucleophile, such as solvent MeCN, to establish a new C–N bond. Closure of the ‘C’-ring through a Mumm rearrangement affords an imide product, excising the ‘O’ atom from the former lactone and introducing a ‘N’ atom in its place.^49,50^ If *FL+_ox_ is a sufficient photoacid, we should obtain the imide product *in lieu* of C–H functionalized products. For this pilot reaction we selected riboflavin tetrabutyrate (RFTB) as a catalyst (10 mol%). RFTB (*λ*_Abs_ = 440 nm, *λ*_Em_ = 519 nm) undergoes irreversible electrooxidation at +1.9 V *vs.* SCE to form the flavinium RFTB+_ox_ (*λ*_Abs_ = 396 nm, *λ*_Em_ = 512 nm), Fig. 1A. The four large butyrate groups give RFTB exceptional solubility in MeCN and shield the photoexcited state from nucleophilic attack, extending its lifetime in solution. The 
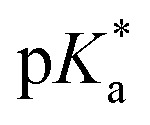
 of RFTB+_ox_ is expected to be in line with other protonated flavins, −5 to −8.^19^ We elected to use an IKA ElectraSyn 2.0 Pro system with an undivided cell, a pair of graphite electrodes, and MeCN solvent. LiClO_4_ was included as an electrolyte and AcOH was added as a cathodic oxidant. An alternating current of +2.1 V *vs.* SCE was applied for 16 h and irradiation was provided by two 370 nm Kessil lights (40 W each). From this reaction, we observed a trace amount of sclareolide-imide product (2). Increasing the loading of RFTB to 50 mol% improved the isolated yield to 17%. The imide product retained the original ‘C’ ring stereochemistry, yielding a single stereoisomer by X-ray crystallography (see ESI, page S105†). No C–H functionalization products were evident. A large fraction of the remaining mass balance is unreacted sclareolide, which was not consumed even with longer reaction times (>4 days). The imide product could not be formed in MeCN through bulk electrolysis at +4.0 V *vs.* SCE, direct photolysis at 370 nm, or treatment with stoichiometric acids such as H_2_SO_4_ and HClO_4_. All conditions led to epimerization and/or carbon skeleton rearrangements, as determined by crude ^1^H NMR of the reaction mixtures.^51,52^ Epimerization with acids like H_2_SO_4_ and HClO_4_ is consistent with reversible proton transfer to the lactone. Proton transfer from *FL+_ox_ is irreversible (p*K*_a_ of ground state FL+_ox_ < 0), obviating epimerization, and allowing the intermediate carbocation that results from C–O bond heterolysis to be trapped by solvent acetonitrile. Ammonolysis with NH_3_ in MeOH also failed to produce the imide product, affording only the ‘C’ ring-opened amide alcohol.^53^ Alternative photoacid catalysts, *i.e.* eosin Y, pyranine, Schreiner's thiourea, and 7-bromonaphthalen-2-ol, were likewise unable to furnish imide 2. One-step formation of an imide 2 was only observed with flavin photoacid catalysis.

We attempted to optimize the reaction by testing alternative organic acids, electrodes, and electrolytes, but none of these changes offered any improvement. We also surveyed different flavin photocatalysts, *e.g.* lumiflavin (R = Me), riboflavin, and riboflavin 5′-monophosphate. Only lumiflavin afforded imide 2 (29% yield), but its cost is prohibitive at the high catalyst loadings needed in our reaction. Based on these results, we performed four sets of mechanistic experiments to gain deeper insight into our reaction. First, we carried out transient spectroelectrochemistry experiments to determine the excited state lifetime of RFTB+_ox_. Passing a current of +1.7 V *vs.* SCE through an MeCN solution of RFTB with irradiation at 390 nm produced *RFTB+_ox_, which decayed over ∼3 ns by fluorescence at ∼512 nm (see ESI, page S44†). A 3 ns lifetime is comparable to riboflavin tetraacetate in 10 mM HClO_4_, *τ* = 2.4 ns, which has a relatively low fluorescence quantum yield, *Φ*_F_ ∼ 0.03.^24^ The fact that *RFTB+_ox_ exhibits a nanosecond lifetime means that it is sufficiently long-lived to engage in bimolecular photocatalysis. One important observation made during these experiments was that extended exposure to light and anodic current converted small amounts of RFTB to several side products. By LC-MS, we tentatively assigned these products as oxygenated and amidated materials, and flavin derivatives having one less butyrate group attached (ESI, page S39†). These same byproducts were detected in our crude reaction mixture with sclareolide. While these products may only form in low amounts, their appearance suggests that at least some of our catalyst degrades over time and this could hinder the efficiency of our reaction. It also provides experimental evidence that some reactive intermediates, such as free radicals derived from MeCN (amidated catalyst) or water (oxygenated catalyst), may still exist in our reaction, and their open-shell chemistries could become more apparent when substrates with non-basic functional groups are employed; a case that we will address later in this manuscript. Second, we performed computations to study the thermochemistry of proton transfer. We used lactone 3 as a computational surrogate for the ‘C’ ring of sclareolide. Ground state proton transfer from a simplified flavin, lumiflavin+_ox_ (LF+_ox_; R = CH_3_), to 3 is endergonic by +7.9 kcal mol^−1^. It is conceivable that this barrier can be overcome by visible light excitation of LF+_ox_. Absorption of a single photon of 370 nm light provides +77 kcal mol^−1^ driving force. In the first singlet excited state, proton transfer becomes exergonic by −7.4 kcal mol^−1^. Our computational analysis shows that proton transfer is only favorable in the excited state, in agreement with the proposed role of RFTB+_ox_ as a photoacid. It is also consistent with the fact that no imide product is formed in the absence of light. Third, we conducted Stern–Volmer quenching studies. We found that sclareolide quenched the excited state of RFTB+_ox_, generated by treatment of RFTB with HClO_4_. Sclareolide failed to quench neutral RFTB. However, the Stern–Volmer plot for RFTB+_ox_ displayed non-linear quenching (ESI, page S15†). At high concentrations of sclareolide, we observed a negative curvature. This occurs when a decrease in the quenching constant is evident and can manifest with a change in the fluorescence spectra of the fluorophore.^54^ It was clear from our experiment that RFTB+_ox_ fluorescence shifted from 510 to 505 nm. The absorption spectrum of RFTB+_ox_ remained constant when subjected to increasing concentrations of sclareolide, ruling out a Stokes' shift due to a change in solvent polarity. A deviation in the fluorescence spectra is also associated with the existence of multiple fluorescent states, with at least two subpopulations of the fluorophore being available. In our case, RFTB+_ox_ can exist in two tautomeric forms where the proton resides on either N1 or N5.^19^ These can interconvert upon absorption and fluorescence (phototropism), accompanied by a dramatic change in charge density, accounting for the observed negative curvature. But this finding does not distinguish between individual mechanisms, *i.e.*, proton transfer, HAT, SET or PCET. To delineate these pathways, we proceeded to our fourth set of mechanistic experiments, that is, the extension of our method to other substrates. We chose two substrates where a photoacid product would be mechanistically distinct from PCET, HAT, SET, base-catalyzed or transition metal catalyzed processes. We initially examined 4-trifluoromethylanisole (4). Trifluoromethylarenes are hydrolyzed under strongly acidic conditions, producing benzoic acids.^55–63^ SET, HAT, or PCET mechanisms lead to S_N_Ar of the methoxy-^64–66^ or trifluoromethyl-group,^67^ electrophilic aromatic substitution,^68^ or α-functionalization of the aryl ether,^69–71^Fig. 2. Benzoic acid 5 was isolated as the *exclusive* product from our reaction. We next examined phenylacetylene (6). Phenylacetylene undergoes aromatic dimerization and N-insertion chemistries when irradiated at 400 nm in the presence of strongly oxidizing (SET) photocatalysts in MeCN solvent.^72^ It can also react with electrophilic free radicals (HAT species) to give β-substituted styrenes or alkyne dimers.^73,74^ We observed none of these products. The *only* product we found was benzylic acetamide 7, which forms from styrene produced by partial reduction of the alkyne at the cathode. Indeed, the use of styrene as reactant also afforded 7, albeit with competitive styrene polymerization. Addition of the amide group to the benzylic carbon is consistent with formation of a carbocation intermediate, expected from a photoacid mechanism. Neither of the two products reported above could be formed by electrolysis, photolysis, acidolysis or ammonolysis. Collectively, our experiments provide evidence for the involvement of RFTB+_ox_ as a photoacid and show that its catalytic efficiency may be compromised by low levels of radical intermediates that survive past the electrodes and covalently modify the catalyst.

**Fig. 3:**
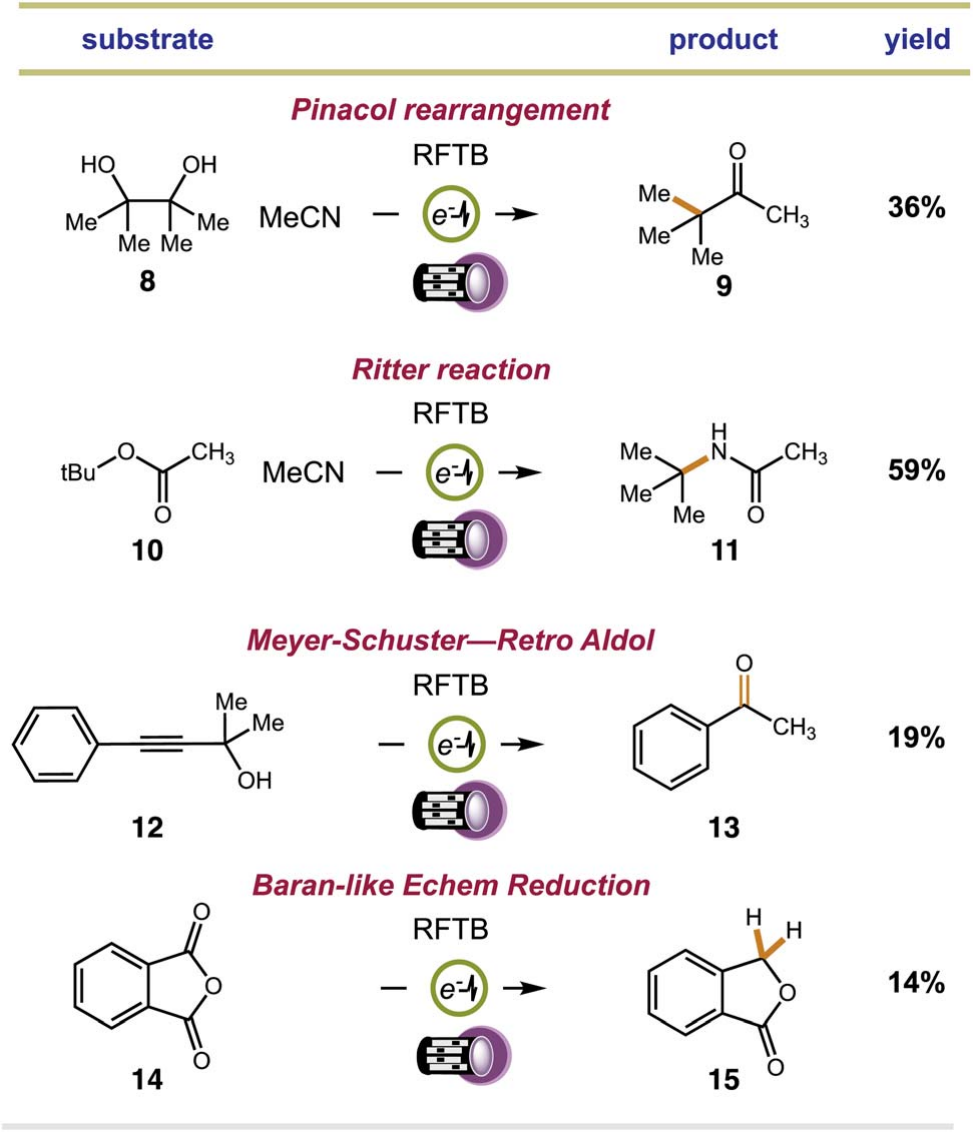
Extension of electrophotochemical flavin photoacid generation to alternative acid-catalyzed reactions.

We examined substrates that are known to react through acid-catalyzed mechanisms to give defined products. Our first efforts focused on the Pinacol rearrangement. Our approach converted pinacol (8) to the classical pinacolone product (9) with transposition of a methyl group. Next, we examined a Ritter reaction with *tert*-butyl acetate (10). Proton transfer from RFTB+_ox_ causes heterolytic C–O bond fragmentation and the release of *t*Bu^+^, which can be trapped by MeCN solvent to give acetamide 11 after hydrolysis. This reaction worked well, yielding *tert*-butyl acetamide 11 in 59% isolated yield. Finally, we examined a Meyer–Schuster reaction with propargyl alcohol 12 and carbonyl reduction with phthalic anhydride 14. For our Meyer–Schuster reaction, we obtained acetophenone (13) as the *exclusive* product. This species can be explained by acid-catalyzed [3,3]-sigmatropic rearrangement of the tertiary alcohol to give the α,β-unsaturated phenone followed by acid-catalyzed conjugate addition of water and a retro aldol. The acid-catalyzed reduction of a single carbonyl group in phthalic anhydride also proceeded smoothly to provide phthalide (15). Here, the flavin photoacid activates the carbonyl group through proton transfer and reducing equivalents of electrons are supplied by the cathode. This type of electrochemical reduction was demonstrated by Baran for a related phthalimide system.^75^ In all cases, we fully acknowledge that many of our isolated yields are ‘low to moderate’. We should mention that all isolations were complicated by co-elution of the desired products with the flavin catalyst. Multiple columns were required to fully remove the catalyst, lowering our isolated yields. Still, despite the lower yields obtained, this is the first time that a flavin has ever been shown to promote acid catalyzed reactions with any success. Moreover, control reactions show that none of the products in Fig. 3 can be made in the absence of either light or current.

Earlier, we showed that in the absence of a suitably basic substrate, that our catalyst is covalently modified. This likely occurs by reaction with background free radical species. Mager *et al.* showed that electrophilic ˙OH can form during flavin electrooxidation, Fig. 1 – path 2. The ˙OH can abstract strong C–H bonds producing open shell C˙ intermediates. If this pathway becomes viable in the absence of non-basic substrates, then we should be able to observe C–H functionalized products in such cases. To test this, we examined cyclohexane as substrate. Cyclohexane contains no functional groups for our flavin photoacid to activate, and it is very unlikely that RFTB+_ox_ is acidic enough to perform hydride abstraction or carbonium ion formation with alkanes, which requires acids with p*K*_a_ < −20.^76^ Applying identical conditions as before, we isolated a small amount of *N*-cyclohexyl acetamide as the sole product from our reaction. A plausible mechanism involves C–H abstraction by ˙OH, electrooxidation of the cyclohexyl radical to a carbocation, trapping by MeCN, and hydrolysis of the transient nitrilium ion to give the final acetamide product. The only byproduct of the reaction is H_2_O, consistent with prior art by Lambert using electrophotochemical conditions.^77,78^ Mager showed that addition of H_2_O to FL+_ox_ affords the 4a-hydroxy flavin, Fig. 1. This species is quite stable but can inevitably dehydrate to return the ground state flavin, which is then anodically re-oxidized. Because the final dehydration event dictates catalyst turnover, we wondered if we could accelerate this step. Glusac *et al.* showed that Lewis-acidic Ce^IV^ can encourage dehydration of 4a-hydroxy flavins by forming metal-hydroxides.^79^ We surveyed a number of Lewis acids for this task, finding a significant improvement with Sc(OTf)_3_. The cooperative effect between FL and Sc(OTf)_3_ is well established, and these two species are often combined to achieve new photocatalytic reactions.^80–82^ Before proceeding, we wished to validate the proposed free radical pathway. We ran a competitive Kinetic Isotope Effect (KIE) experiment with d12-cyclohexane and performed Stern–Volmer quenching studies with cyclohexane, *2ScRFTB and *RFTB+_ox_ (ESI, pages S11–S14†). We obtained a KIE = 1.2, which is not typically consistent with Csp^3^–H HAT in the rate-determining step, especially by highly reactive radicals like ˙OH (*k* ∼ 10^12^ cm^3^ per molecule per s)^83^ or others that can be generated by electrophotochemical methods to activate C–H bonds.^84–87^ In our case, generation of hydroxyl radicals, which was experimentally validated using methylene blue quenching of our crude reaction mixture—see ESI, pages S40–S41†—from 4a-hydroxyflavin radical cation is expected to be a much slower process,^88^ and likely rate-determining, which better reflects our KIE value. We documented no quenching of any flavin species with cyclohexane, occluding the possibility of SET, proton transfer, or energy transfer.

**Fig. 4:**
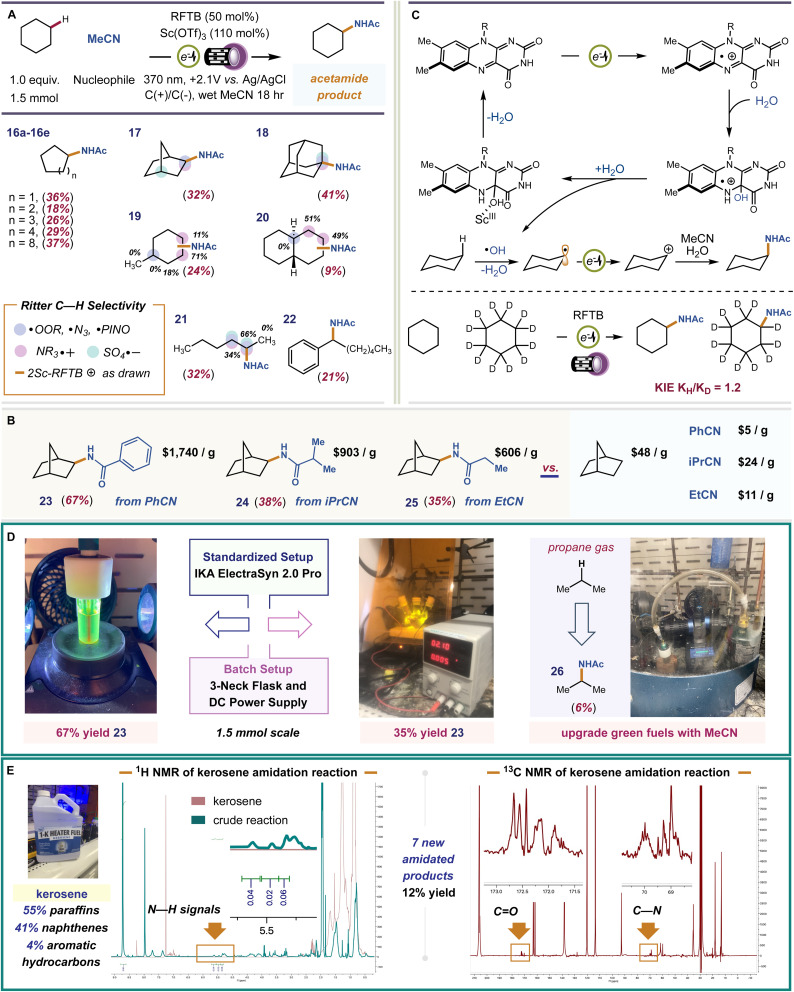
Scope of the reaction and application to hydrocarbon fuels. (A) Survey of alkane substrates and a comparison of observed site-selectivities to established amidation protocols (Ritter-type) that use different catalysts/mediators. % Regiochemical distribution for substrates with multiple products is indicated beside each site. (B) Scope of alternative nitrile nucleophiles with average prices of products and starting materials from commercial vendors. (C) Proposed mechanism of C–H amidation. (D) Results with gaseous propane and a comparison of reaction efficiencies between our standard IKA ElectraSyn 2.0 Pro setup and a three-neck flask electrochemical setup. (E) Experiments performed using commercial kerosene as C–H substrate.

We examined the scope and chemoselectivity of alkane amidation. For most alkanes, poor solubility was observed in MeCN solvent. Therefore, we included trichloroacetonitrile (TCA) as a cosolvent. TCA greatly improves solubility without hindering reaction efficiency or competing with MeCN in the solvent trapping step. We are aware that TCA can expel Cl˙ when irradiated with UV light and that Cl˙ can abstract hydrogen atoms from alkanes.^89^ In a control reaction with 370 nm or 254 nm lights and 14 : 5 MeCN/TCA solvent, no product was formed when RFTB was excluded, showing that Cl˙ does not engender product formation when TCA is used as a cosolvent. We found that cycloalkanes C_5_–C_12_ (compounds 16a–e, *E*^0^_ox_ = 2.3–2.6 V *vs.* SCE)^90^ were converted to new acetamide products, isolated yields 18–37%. For alkanes with both 2° and 3° C–H bonds (compounds 17–20), we observed site-selectivities consistent with Ritter-type amidation procedures mediated by an electrophilic HAT species, like ˙OH.^91–101^ For instance, in methylcyclohexane, amidation at more sterically congested C2 is preferred over less crowded C4. The difference is that C2 has 2-fold more hydrogen atoms to choose from, mirroring C3. The fact that C3 has four hydrogen atoms and is sterically accessible makes it the most preferred site for kinetic C–H amidation. Our observed selectivity, C3 > C2 > C4 is reflective of some evolved P450 nitrene-transferases^102^ and metallaphotoredox platforms.^103^ This same effect can be seen for *trans*-decalin 20 and *n*-hexane 21. When the number of hydrogen atoms is equal, the methylene site that is most accessible is amidated. Thus, a general set of rules for predicting site-selectivity in alkane substrates is established. These regiochemical preferences should hold true regardless of the selected nitrile (nucleophile) or the experimental setup used to perform our reaction. To test this, we selected three commercial nitriles that replace acetonitrile as solvent and nucleophile, and a three-neck round bottom flask equipped with a DC power supply and graphite electrodes as a substitute for our standard IKA ElectraSyn 2.0 Pro electrochemical setup. For all experiments, norbornane was used as the test alkane and TCA was omitted due to improved solubility, Fig. 4B and D. All three nitriles fashioned a single amide product (compounds 23–25, yields 35–67%) that would be very expensive to procure otherwise. The site- and stereo-selectivities of the new amide products were identical to reactions performed with acetonitrile. In a three-neck flask, 23 was formed in 35% isolated yield. Despite its lower efficiency, this proof-of-principle experiment is evidence that our reaction can be performed in alternative setups, including those that are more conducive to large-scale batch reactions, without loss in product selectivity. Finally, we tested the capacity of our catalyst to upgrade off-the-shelf samples of common hydrocarbon fuels. The hydroxy radical formed by flavin electrooxidation is highly reactive and, therefore, can abstract a wide variety of C–H bonds (1°, 2°, and 3°) in different alkanes, improving feedstock throughput. This is harder to achieve with more selective HAT reagents that may only activate a select few hydrogen atoms, 2° and 3°. Our preliminary studies focused on gaseous propane (*E*^0^_ox_ = 2.90 V *vs.* SCE) and kerosene (jet fuel), a primary distillate from crude oil that contains a heterogeneous mixture of C9–C16 alkanes and alkylarenes (Fig. 4D and E). Functionalization of C–H bonds in light alkanes (C_1_–C_4_) is intrinsically more difficult because of their higher bond-dissociation energies (BDE) and low polarities. Under our electrophotochemical conditions, *N*-isopropylacetamide (26) was forged in 6% LC-MS yield (Fig. 4D). For kerosene, our conditions produced a ∼12% NMR yield of amidated products, which includes ∼7 unique amides by ^1^H–^13^C HMBC. Thus, our system can harness petroleum side streams as veritable bench deposits to pan out value-added amide products.

## Conclusions

In summary, we show that electrophotochemistry provides a direct route to access flavin photoacids, allowing them to be harnessed in organic synthesis for the first time. We show that when substrates lacking a basic functional group are employed that ˙OH formation becomes more apparent, enabling C–H amidation protocols. Together, our results demonstrate the unique ability of flavins (FL+_ox_) to exhibit bimodal reactivity under electrophotochemical conditions, unveiling new mechanistic opportunities for using flavins as dynamic electrophotocatalysts.

## Data availability

Crystallographic data for compound 2 has been deposited at the Cambridge Crystallographic Data Centre (CCDC) under accession number 2255574. All other data is present in the manuscript or ESI.†

## Author contributions

S. Ga. performed all chemical reactions. J. W. and S. Go. acquired all spectroelectrochemical spectra. S. B. supervised this work. The manuscript was written with input and approval from all authors.

## Conflicts of interest

There are no conflicts to declare.

## Supplementary Material

SC-015-D4SC03054K-s001

SC-015-D4SC03054K-s002
