# Supplementary material for: Unlocking flavin photoacid catalysis through electrophotochemistry
Source: Chem Sci. 2024 Jun 18;15(29):11444–54. doi: 10.1039/d4sc03054k (PMC11268482; doi:10.1039/d4sc03054k)
Supplement: SC-015-D4SC03054K-s001 [file SC-015-D4SC03054K-s001.pdf]

Electronic Supplementary Material (ESI) for Chemical Science.
This journal is © The Royal Society of Chemistry 2024

Unlocking Flavin Photoacid Catalysis through Electrophotochemistry

Samuel Gary, Jack M. Woolley, Sofia Goia, and Steven Bloom*

Corresponding author: spbloom@ku.edu

Table of Contents

1. General Information 2
2. Synthetic Procedures 2
3. UV/Vis, Fluorescence, and Fluorescence Quenching Studies 9
4. Cyclic Voltammetry 16
5. Calibration Curve Data and Control Reactions 33
6. Transient Absorption Spectroelectrochemistry 42
7. Product Characterization 47
8. NMR Spectra 58
9. Crystallographic Data for Compound 2 105
10. Computations 118
11. References 133

1. General Information

All substrates were obtained from commercial vendors (TCI America, Combi-Blocks, Sigma-Aldrich, and Oakwood Chemicals) and used as received. Electrochemical reactions were performed using an IKA ElectraSyn 2.0 Pro system. Spectroelectrochemical measurements were performed using a PalmSens EmStat3 potentiostat. Two Kessil 370 nm LEDs were used for irradiation. Normal phase chromatography was performed on an Isolera 1 Biotage instrument with the indicated solvent systems with Buchi FlashPure EcoFlex prepacked columns. Reverse phase chromatography was performed on a Buchi Pure C-815 instrument S3 with Biotage Sfär C18 Duo prepacked columns with the indicated solvent system. LC-MS spectra were acquired on a Waters Acquity UPLC HClass equipped with a Waters QDa Mass Detector using a Discovery BIO Wide Pore C8 HPLC column (Supelco; 5 μm, 10 cm x 4.6 mm) or an XBridge BEH C18 column (Waters; 2.5 μm, 150 cm x 4.6 mm). Electrospray ionization spectra were acquired on a LCT Premier (Waters Corp.) time of flight mass spectrometer (HRMS). NMR spectra were obtained on a a Bruker Ascend 400 NMR spectrometer or an Avance AVIII 500 MHz spectrometer equipped with a multi-nuclear BBFO cryoprobe. Peak multiplicities are designated by the following abbreviations: br, broad; s, singlet; d, doublet; t, triplet; q, quartet; quint, quintet; sept, septet; m, multiplet; dd, doublet of doublets; dt, doublet of triplets; tt, triplet of triplets; and ddd, doublet of doublet of doublets. ^{1}H NMR and ^{13}C NMR spectra were referenced to residual solvent peaks.

2. Synthetic Procedures

Standard Procedure for Photoacid Chemistry

To a 20-mL ElectraSyn vial equipped with a magnetic stir bar was added riboflavin tetrabutryate (493 mg, 0.75 mmol), $LiClO_4$ (404 mg, 3.8 mmol), and glacial AcOH (858 μL, 15 mmol). MeCN (19 mL) and substrate (1.5 mmol) were added, the vial was capped with a septum, and the solution was degassed with N_2 for 5 minutes. All reactions were conducted using graphite as both the cathode and anode material. A silver wire (submerged in 3 M aq. KCl) was used as a reference electrode. Reactions were set to maintain a constant voltage of +2.1 V for 18 hours with alternating polarity every 1 minute. Two 370 nm LEDs were placed ~ 1 inch from either side of the vial, and the temperature was maintained near-ambient with a household fan. At the completion of the reaction, the solvent was evaporated under reduced pressure. The reaction mixture was redissolved in ethyl acetate (70 mL) and extracted with sat. aq. $NaHCO_3$ (50 mL). The aqueous layer was extracted with ethyl acetate twice more (2 x 70 mL) and the combined organic layers were washed with brine (100 mL). The organic layer was dried over $MgSO_4$, filtered, and concentrated under reduced pressure. The product was then purified by flash chromatography as indicated.

Standard Procedure for Amidation of Alkanes

To a 20-mL ElectraSyn vial equipped with a magnetic stir bar was added riboflavin tetrabutyrate (493 mg, 0.75 mmol), $Sc(OTf)_3 \bullet H_2O$ (842 mg, 1.65 mmol), $LiClO_4$ (404 mg, 3.8 mmol), and glacial AcOH (858 μL, 15 mmol). MeCN (HPLC grade, containing ~1% water; 14 mL) and trichloroacetonitrile (5 mL) were added and the solution was degassed with nitrogen for 5 minutes. The alkane substrate (1.5 mmol) was then added and the vial capped with a septum. All reactions were conducted using graphite as both the cathode and anode material. A silver wire (submerged in 3 M aq. KCl) was used as a reference electrode. Reactions were set to maintain a constant voltage of +2.1 V for 18 hours with alternating polarity every 1 minute. Two 370 nm LEDs were placed ~ 1 inch from either side of the vial, and the temperature was maintained near-ambient with a household fan. At the completion of the reaction, the solvent was evaporated under reduced pressure and directly purified by column chromatography.

Notes:

– The use of alternating polarity is not required for the reaction to be successful, although it does greatly increase the lifetime of the electrodes.

– For the majority of products, reverse phase chromatography was the only viable means of purification. Under a variety of either common or exotic solvent systems using normal phase chromatography, products co-eluted with catalyst and/or catalyst degradation products.

– All alkane products were lyophilized prior to characterization. However, most products retain traces of methanol, acetone, and/or water in NMR spectra.

Procedure for Amidation of Kerosene

The procedure above was followed except with the use of an additional cooling fan to ensure ambient temperature was maintained. Kersosene (315 μL) was used on the basis of the reported average molecular weight of kerosene components,[1] which served as an approximation for the standard 1.5 mmol scale. After the completion of the reaction, the solvent was removed under reduced pressure and the reaction mixture was directly loaded onto a C18 column. Water was used to flush paramagnetic salts (e.g., Sc^{III}) from the mixture for several minutes. (Note: this step was essential in obtaining usable NMR data). The remaining components were eluted with 95:5 acetone:water, and all of the resulting fractions were combined, concentrated, and lyophilized. Prior to NMR analysis, 1,2,4,5-tetracyanobenzene (1.5 mmol) was added as an internal standard.

Procedure for Amidation of Propane

For determination of the quantity of propane that was added, propane gas was bubbled through a solution of 14:5 MeCN:trichloroacetonitrile for 30 minutes. A 500 μL aliquot was directly added to an NMR tube, along with 0.10 mmol of CH_2Br_2 as an internal standard, which was sealed and immediately analyzed by ^1H NMR. From this experiment, a solubility of 72 mM was determined for propane. This led to the electrophotochemical reaction being performed on a slightly lower 1.44 mmol scale. The reaction was conducted under the same procedure, with propane being bubbled through the full reaction mixture for 30 minutes prior to the start of the reaction.

Procedure for Batch Reaction

The standard procedure for amidation was followed using 19 mL of benzonitrile as solvent and norbornane as substrate, except the reaction vessel was a three-neck round bottom flask and an external DC power supply was used (see pictures below). 113 mg (35% isolated yield) was obtained of **7** as the same spectral data as obtained from our standardized reaction.

Figure S1. Close-up photograph of batch setup.

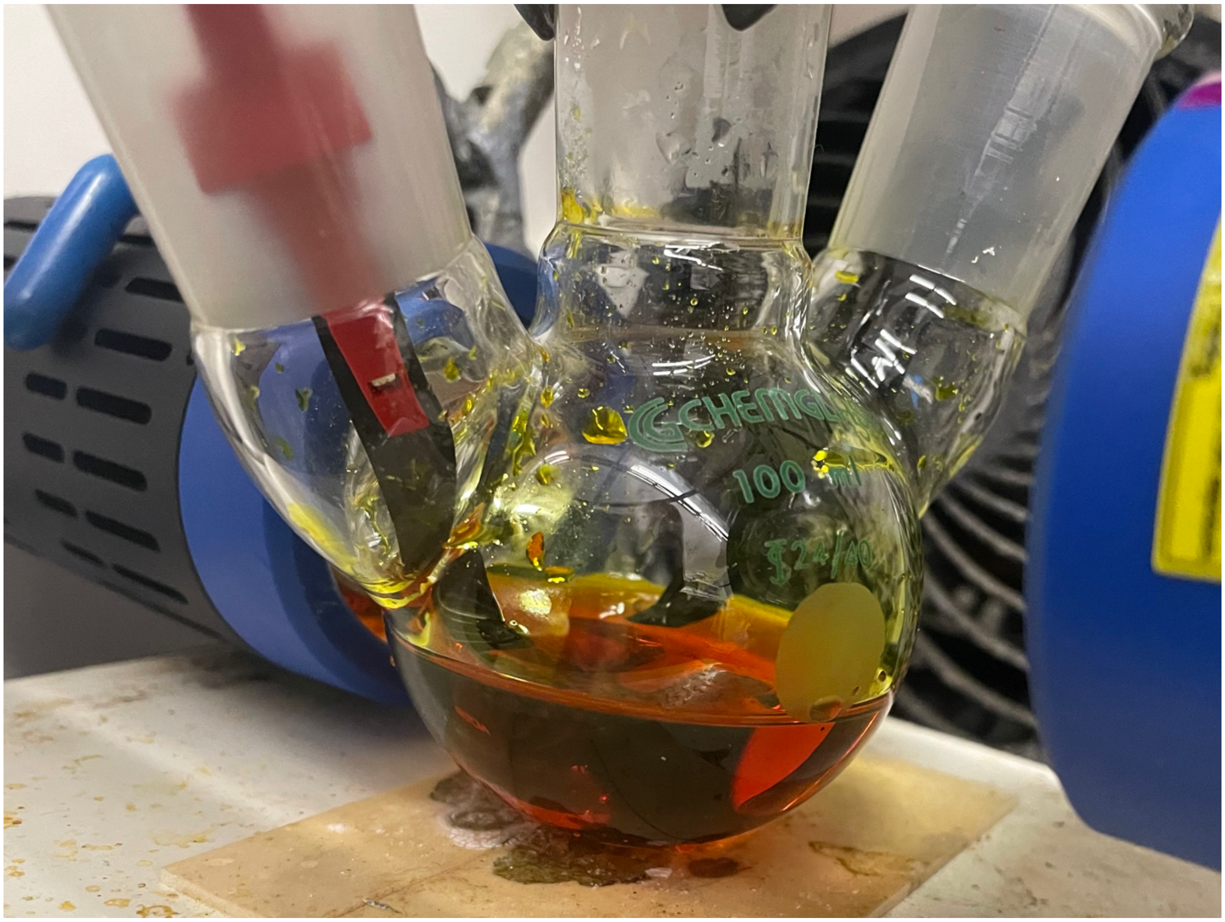

Figure S2. Full-scale photograph of batch setup.

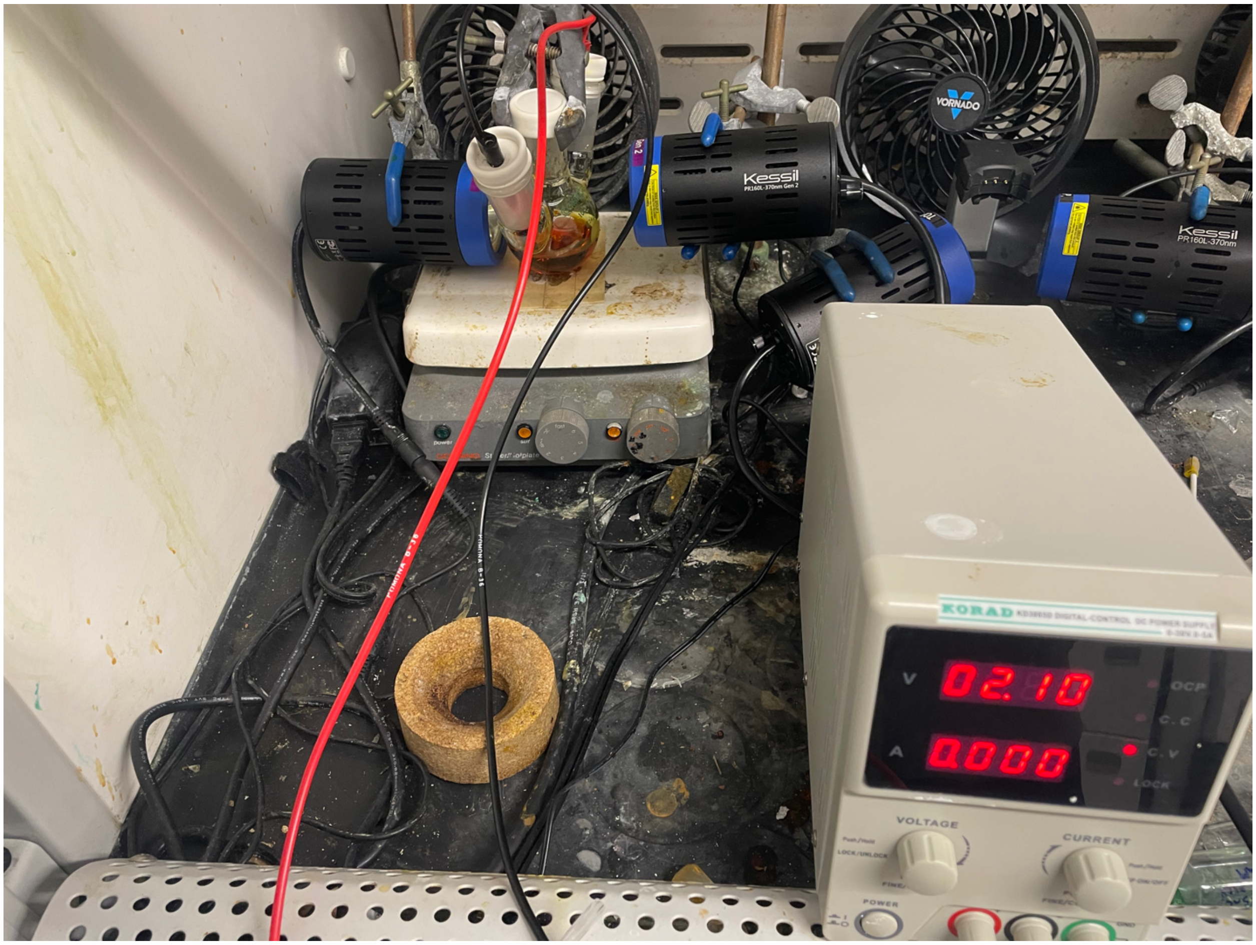

Figure S3. Photograph of illuminated batch setup.

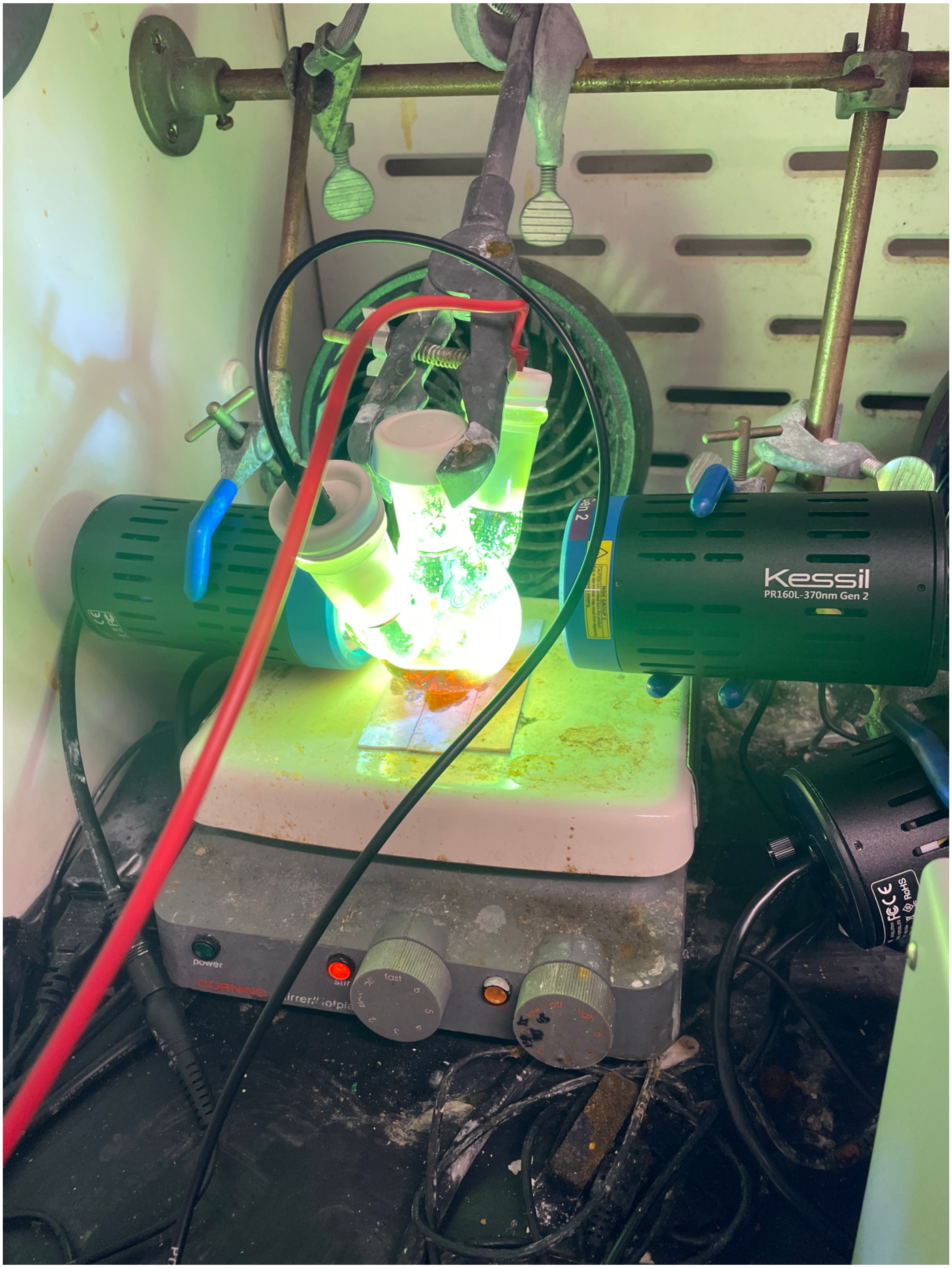

Figure S4. Photograph of illuminated standardized (IKA ElectraSyn 2.0 Pro) setup.

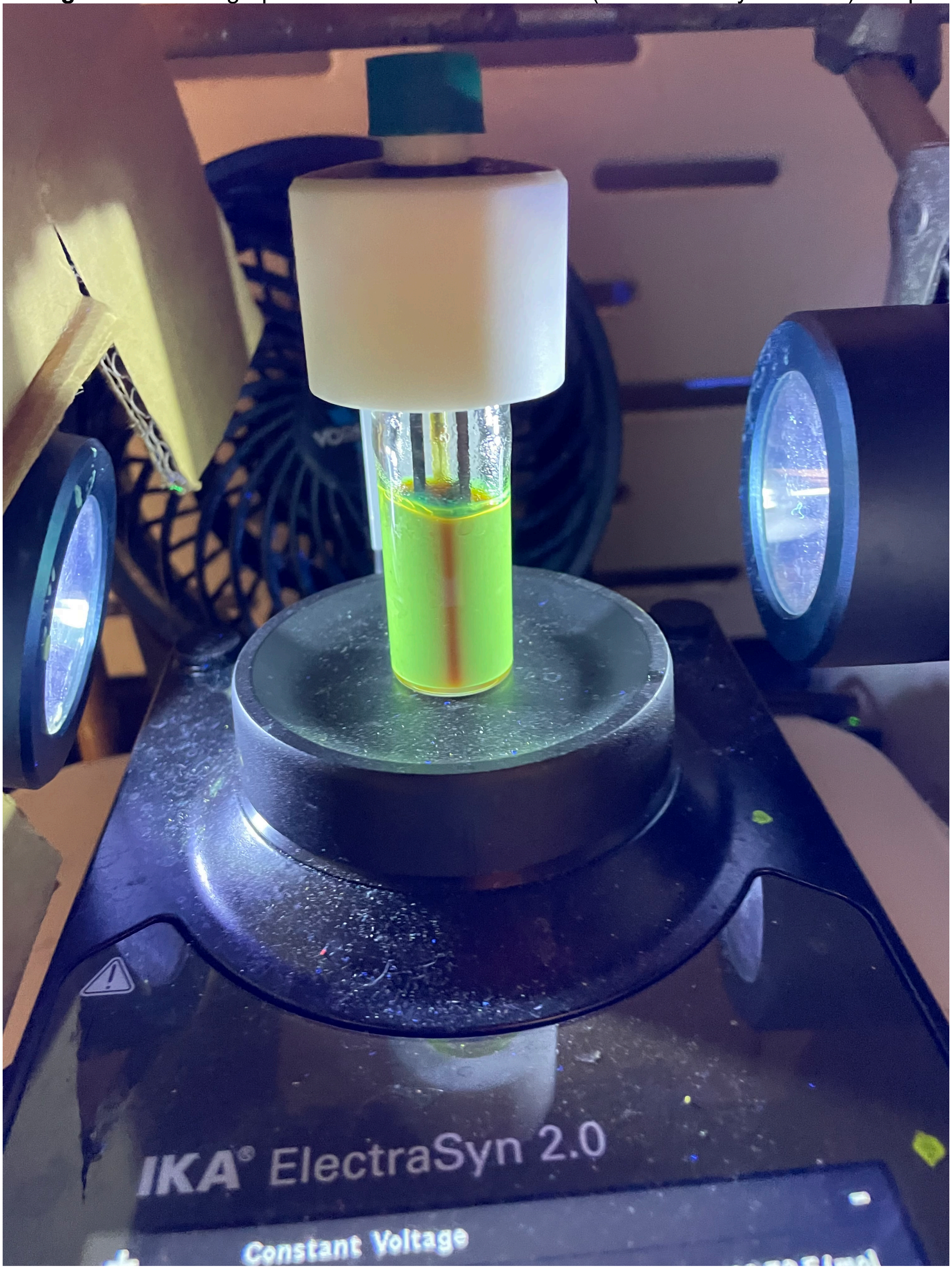

3. UV/Vis, Fluorescence, and Fluorescence Quenching Studies

General Materials and Methods

Absorbance spectra were acquired on a Shimadzu UV-2600i spectrophotometer. For catalyst absorption spectra, a solution of 14:5 acetonitrile:trichloroacetonitrile was used as solvent. For spectra containing $Sc(OTf)_3$, 5.0 equivalents relative to RFTB was added. For spectra referring to RFTB+ perchlorate, 1 equivalent of $HClO_4$ relative to RFTB was added first. All spectra were obtained using 1-nm increments.

Fluorescence spectra were acquired on a QuantaMaster Luminescence Spectrometer (Photon Technologies, Inc.). For all Stern-Volmer quenching studies with $Sc(OTf)_3$, an excitation wavelength of 383 nm was used. For all Stern-Volmer quenching studies with HRFTB+ or 2Sc-RFTB, an excitation wavelength of 390 nm was used. Increasing concentrations (0-500 equivalents) of substrate were added to 2.0 mL of RFTB, from a stock solution of 0.99 μM of RFTB in acetonitrile. For quenching studies using $Sc(OTf)_3$, 4.95 μM $Sc(OTf)_3$ was added. Spectra were acquired at 1 nm/second from 420-700 nm. Spectra were plotted on a single scatter plot, and Stern-Volmer plots were obtained using the fluorescence intensity at 515 nm, which corresponds to the fluorescence maximum for RFTB.

Figure S5. Absorbance spectrum of RFTB in the presence and absence of $Sc(OTf)_3$.

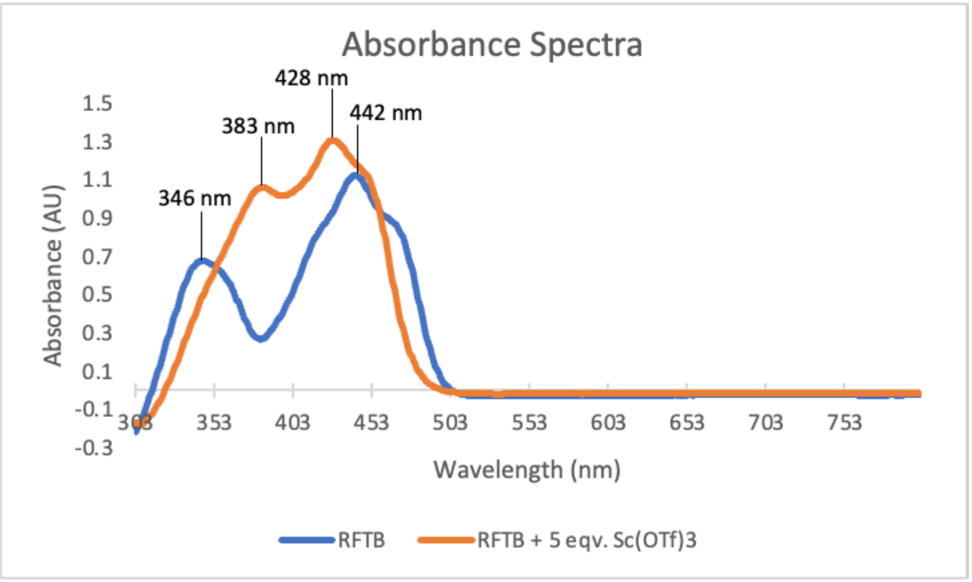

Figure S6. Absorbance spectrum of RFTB+ perchlorate.

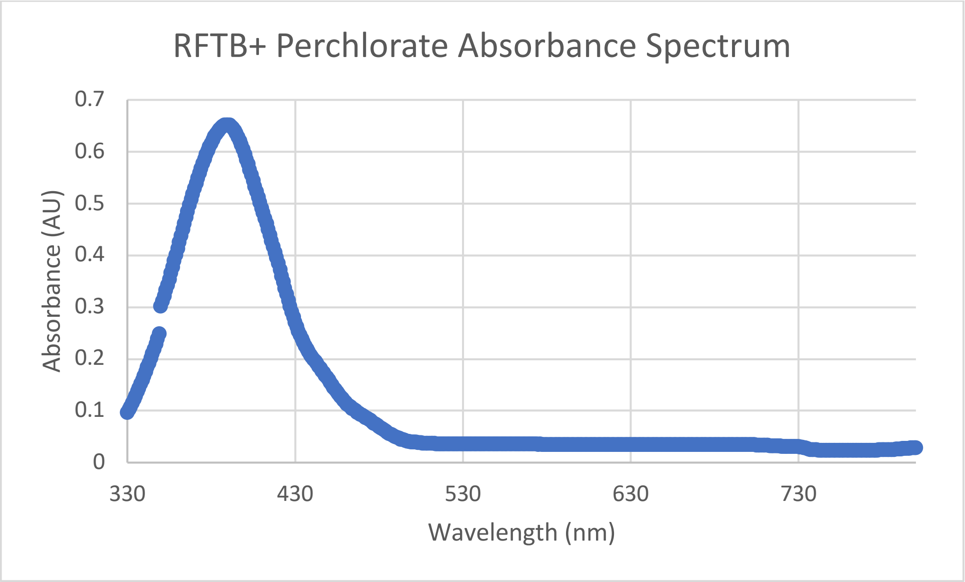

Figure S7. Absorbance spectrum of RFTB before and after oxidation (+1.7 V *vs* Ag/AgCl) for 10 minutes (MeCN, 0.1 M NBu_4PF_6).

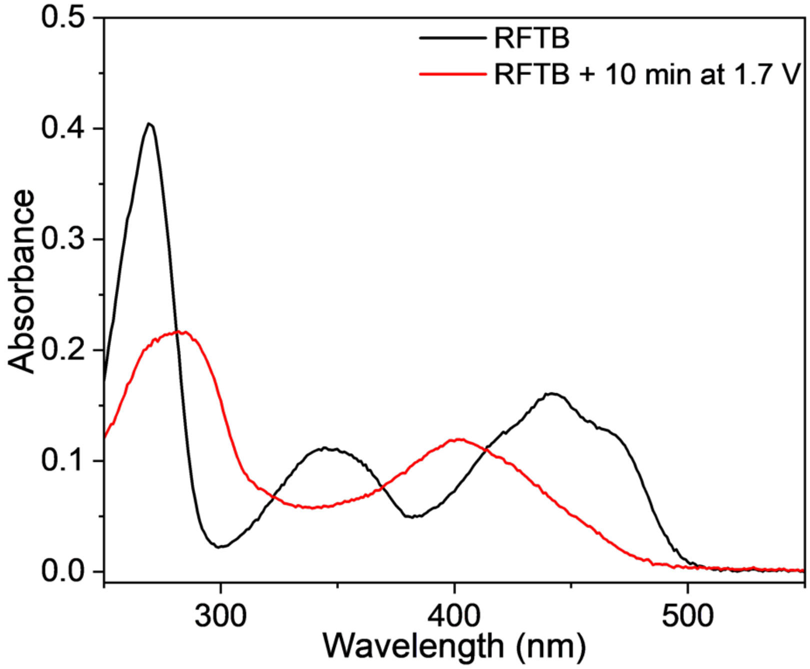

Figure S8. Fluorescence spectrum of 2Sc-RFTB with increasing concentrations of norbornane.

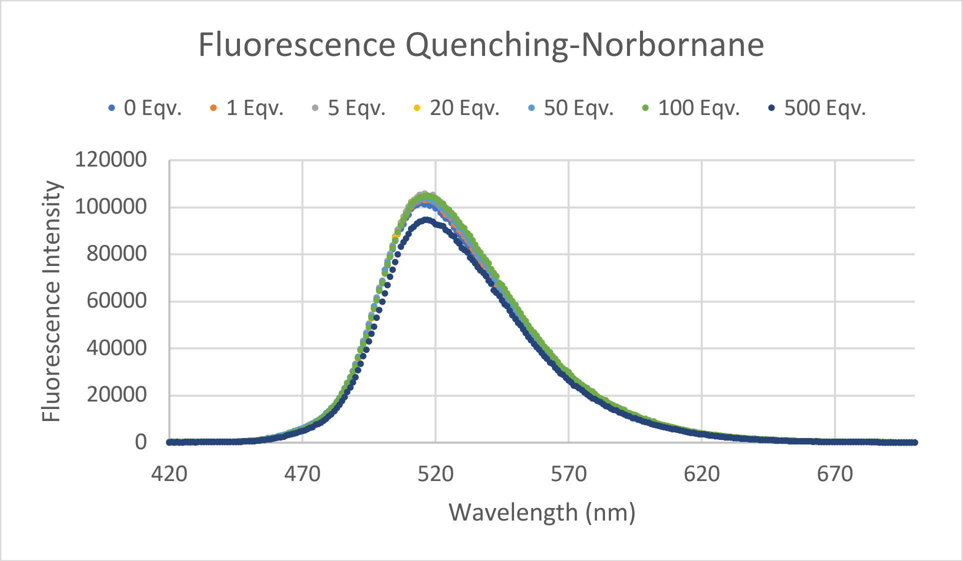

Figure S9. Stern-Volmer Plot with norbornane as quencher.

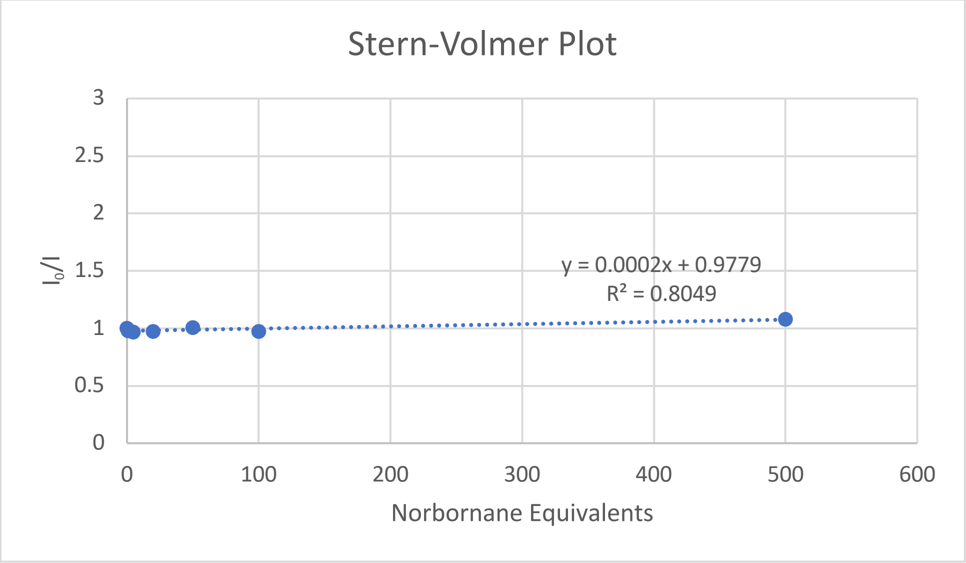

Figure S10. Fluorescence spectrum of 2Sc-RFTB with increasing concentrations of *n*-hexane.

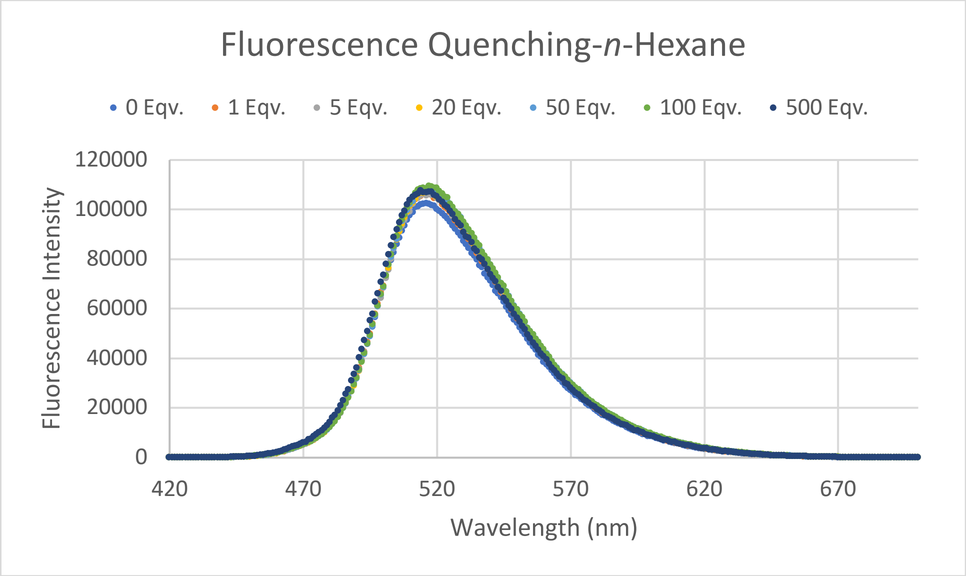

Figure S11. Stern-Volmer Plot with *n*-hexane as quencher.

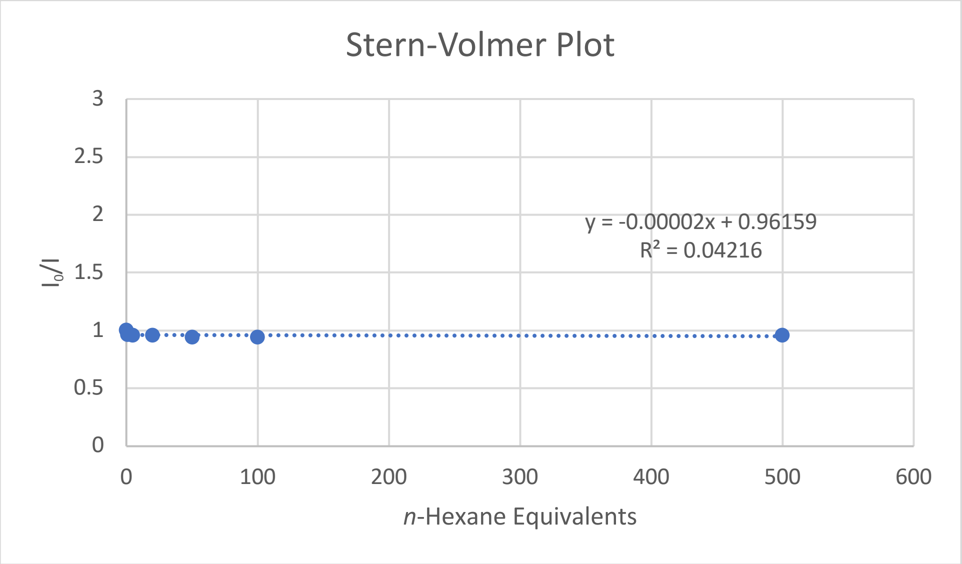

Figure S12. Fluorescence spectrum of 2Sc-RFTB with increasing concentrations of cyclohexane.

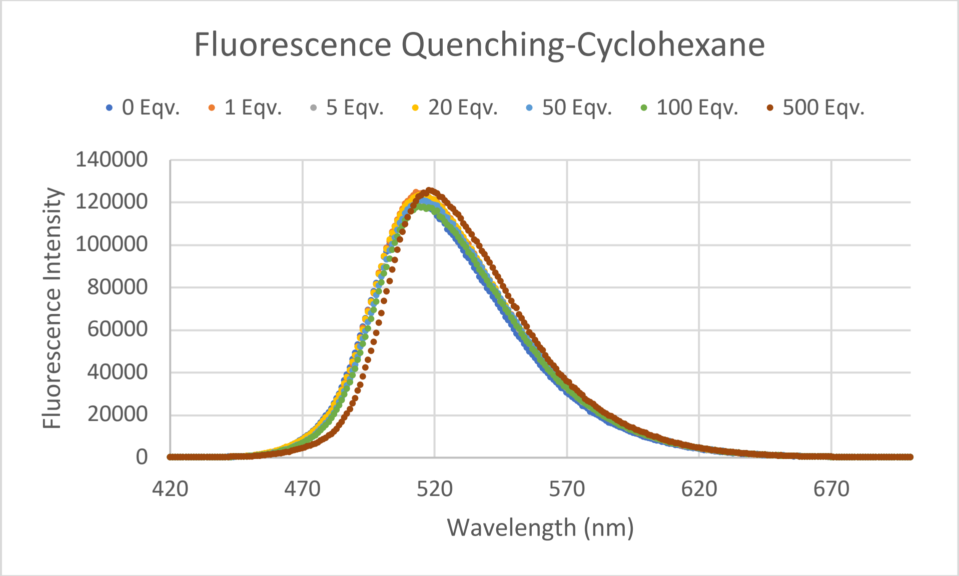

Figure S13. Stern-Volmer Plot with cyclohexane as quencher.

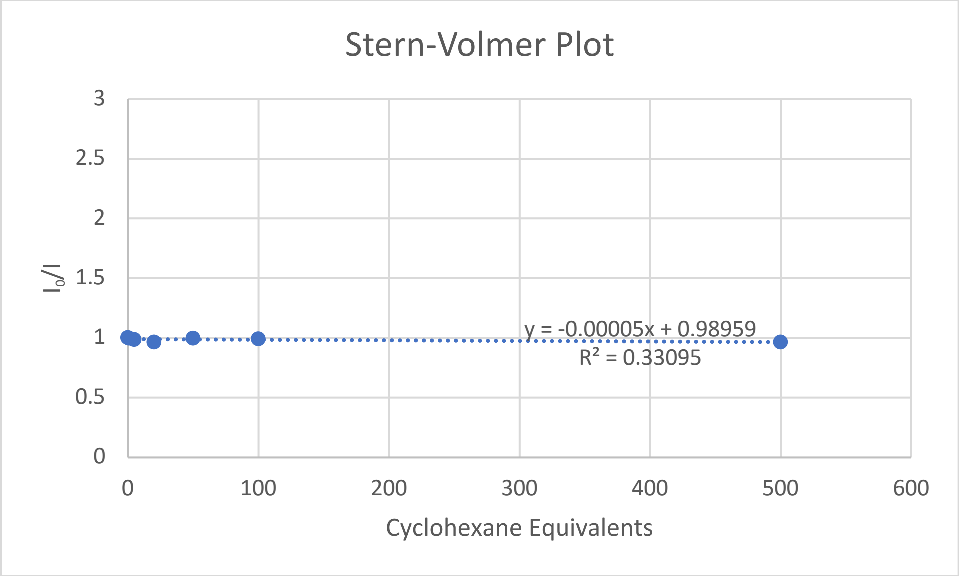

Figure S14. Fluorescence spectrum of RFTB+ perchlorate with increasing concentrations of cyclohexane.

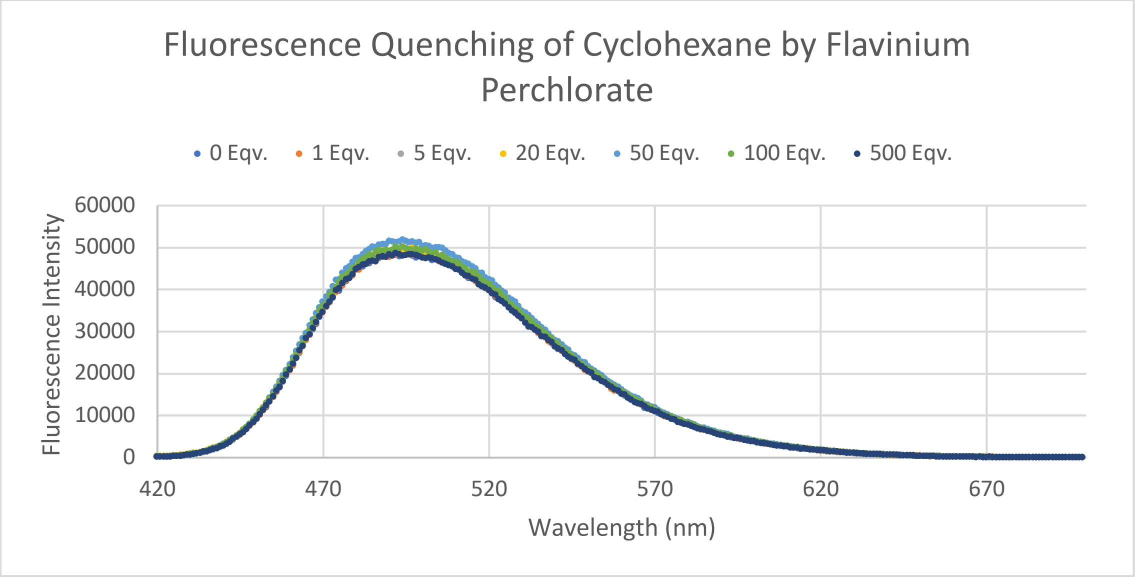

Figure S15. Stern-Volmer plot of RFTB+ perchlorate with cyclohexane as quencher.

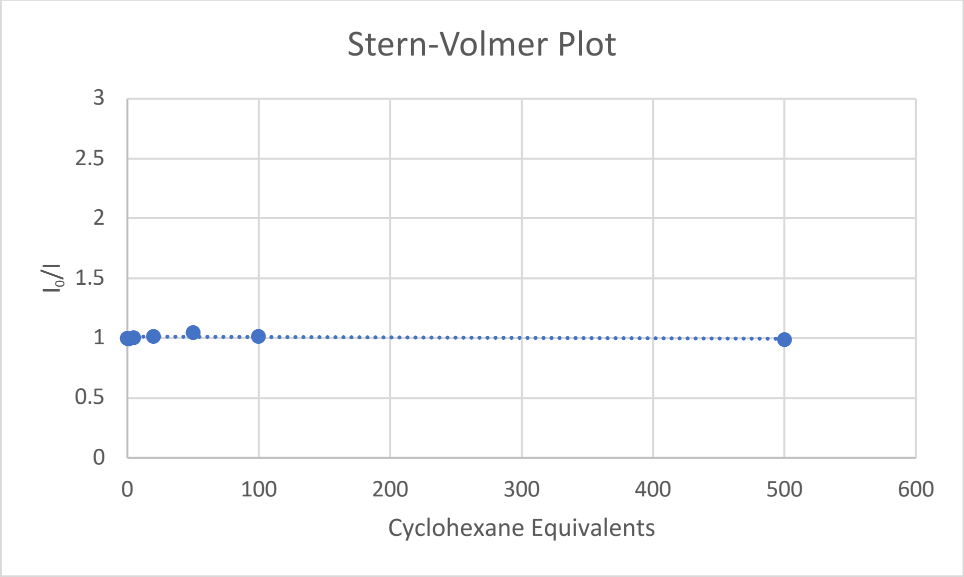

Figure S16. Fluorescence spectrum of RFTB+ perchlorate with increasing concentrations of sclareolide.

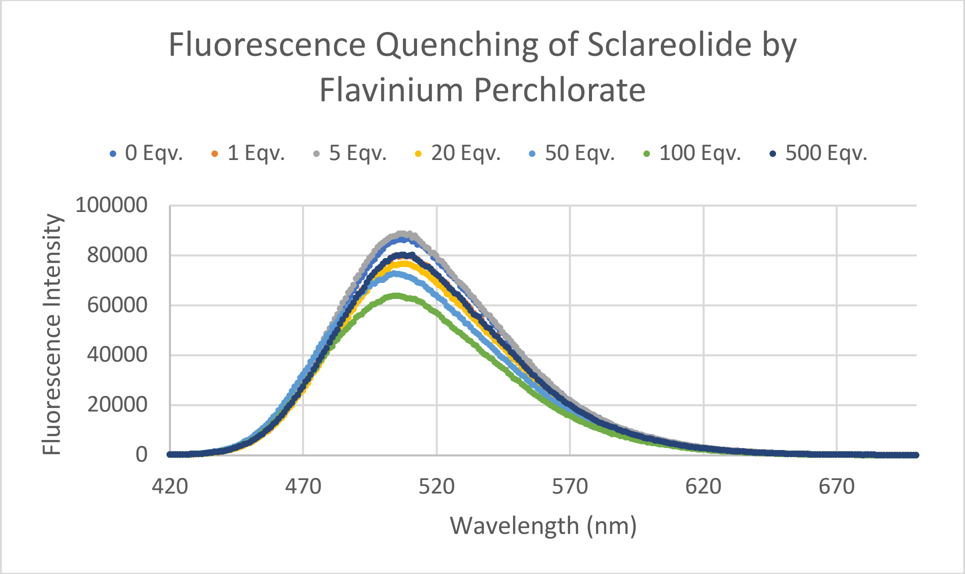

Figure S17. Stern-Volmer plot of RFTB+ perchlorate with sclareolide as quencher.

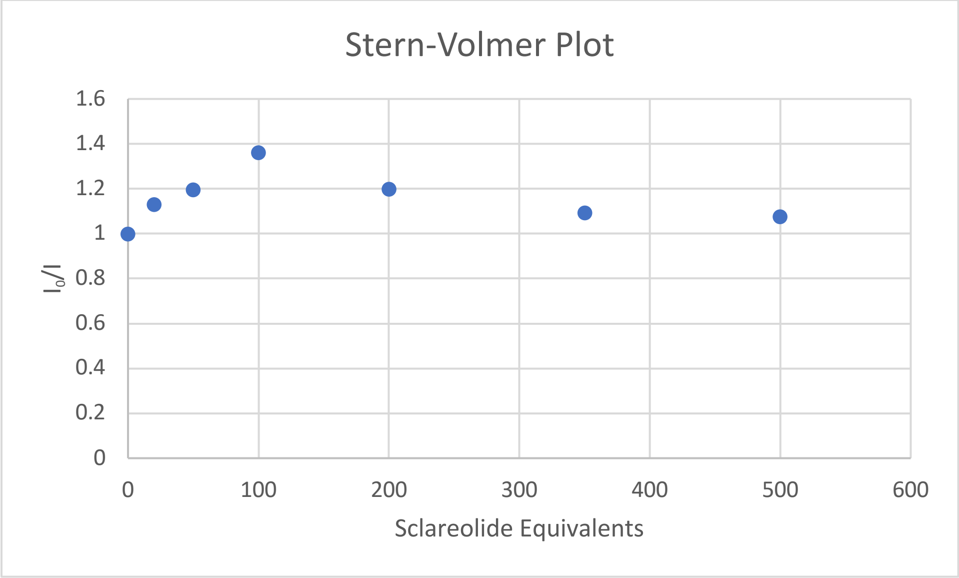

4. Cyclic Voltammetry

General Materials and Methods

Cyclic voltammetry for all substrates was acquired using an IKA ElectraSyn 2.0 in nitrogen-degassed 4:1 (v:v) MeCN:EtOAc containing 0.1M $LiClO_4$. A glassy carbon working electrode (8 x 52.5 x 2 mm), platinum counter electrode, and silver wire reference electrode (3M aq. KCl) were used to acquire CV spectra. CV spectra were obtained using the following parameters: initial voltage 0.0 V, upper voltage 3.0 V, final voltage 0.0 V, and a scan rate of 50 mV/sec. Substrate concentrations ranged from 100-400 mg in 10 mL of solvent. For RFTB, a scan rate of 100 mV/sec was used and 0.1M NBu_4PF_6 in MeCN was used as supporting electrolyte (reversibility was not observed using $LiClO_4$). For 2Sc-RFTB, 2.0 equivalents of $Sc(OTf)_3$ was added, a scan rate of 10 mV/sec was used, and 0.1M NBu_4PF_6 was used as supporting electrolyte. Oxidation potentials for substrates are reported as E^0_{ox}, i.e., the maximum of the oxidation wave peak. Values were obtained *vs*. Ag/AgCl and converted to SCE by subtracting 0.045 V. For substrates displaying multiple oxidation peaks, only the first peak is reported. For cyclohexane, the solvent used for CV acquisition was 4:1 (v:v) EtCN:EtOAc with 0.1M $LiClO_4$.

For the spectroelectrochemical experiments performed on 0.1 mM riboflavin tetrabutyrate (MeCN, 0.1 M NBu_4PF_6), a CV was obtained prior to each measurement using a bespoke non-aqueous setup based on previous published work[2]. The three-electrode system included a boron doped diamond mesh working electrode, an Ag/AgCl reference electrode, and a platinum wire counter electrode. The CVs were measured at a 0.1 V/sec scan rate, between −1.0 V and 2 V *vs*. Ag/AgCl.

Table S1. Measured oxidation potentials (*vs.* SCE) of substrates.

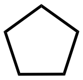	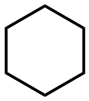	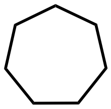	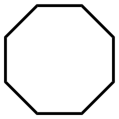	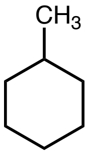
E^0_{ox} = 2.40V	E^0_{ox} = 2.62V	E^0_{ox} = 2.29V	E^0_{ox} = 2.50V	E^0_{ox} = 2.15V
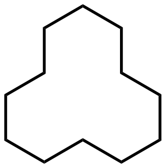	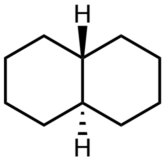	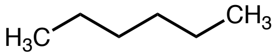	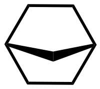	
E^0_{ox} = 2.14V	E^0_{ox} = 2.30V	E^0_{ox} = 2.44V	E^0_{ox} = 2.31V	
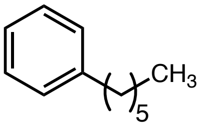	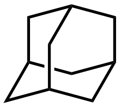	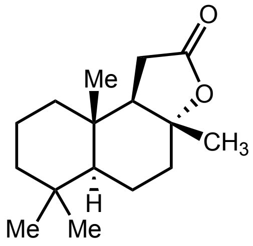	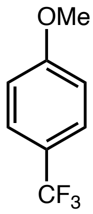	
E^0_{ox} = 2.19V	E^0_{ox} = 2.34V	E^0_{ox} = 2.58V	E^0_{ox} = 2.62V	

Figure S18. Cyclic voltammetry spectrum of cyclopentane.

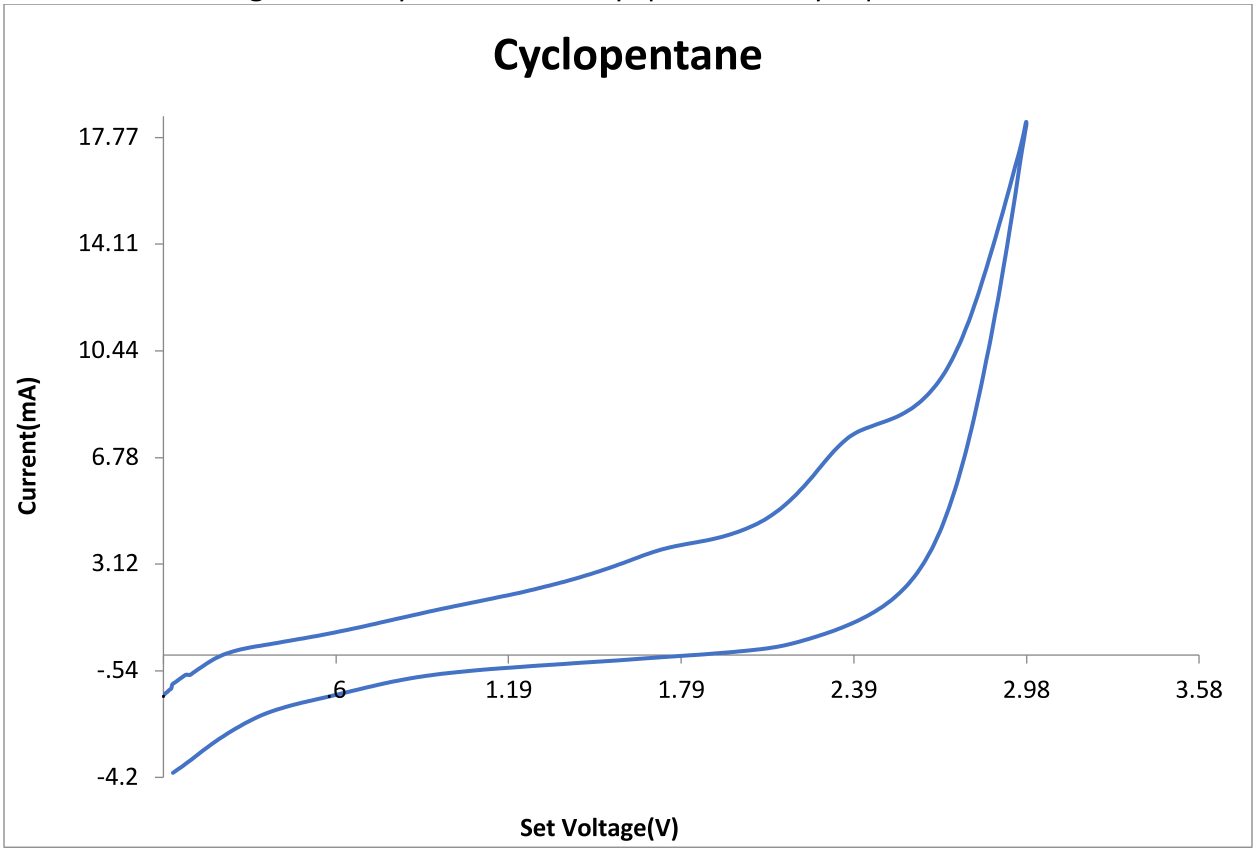

Figure S19. Cyclic voltammetry spectrum of cyclohexane.

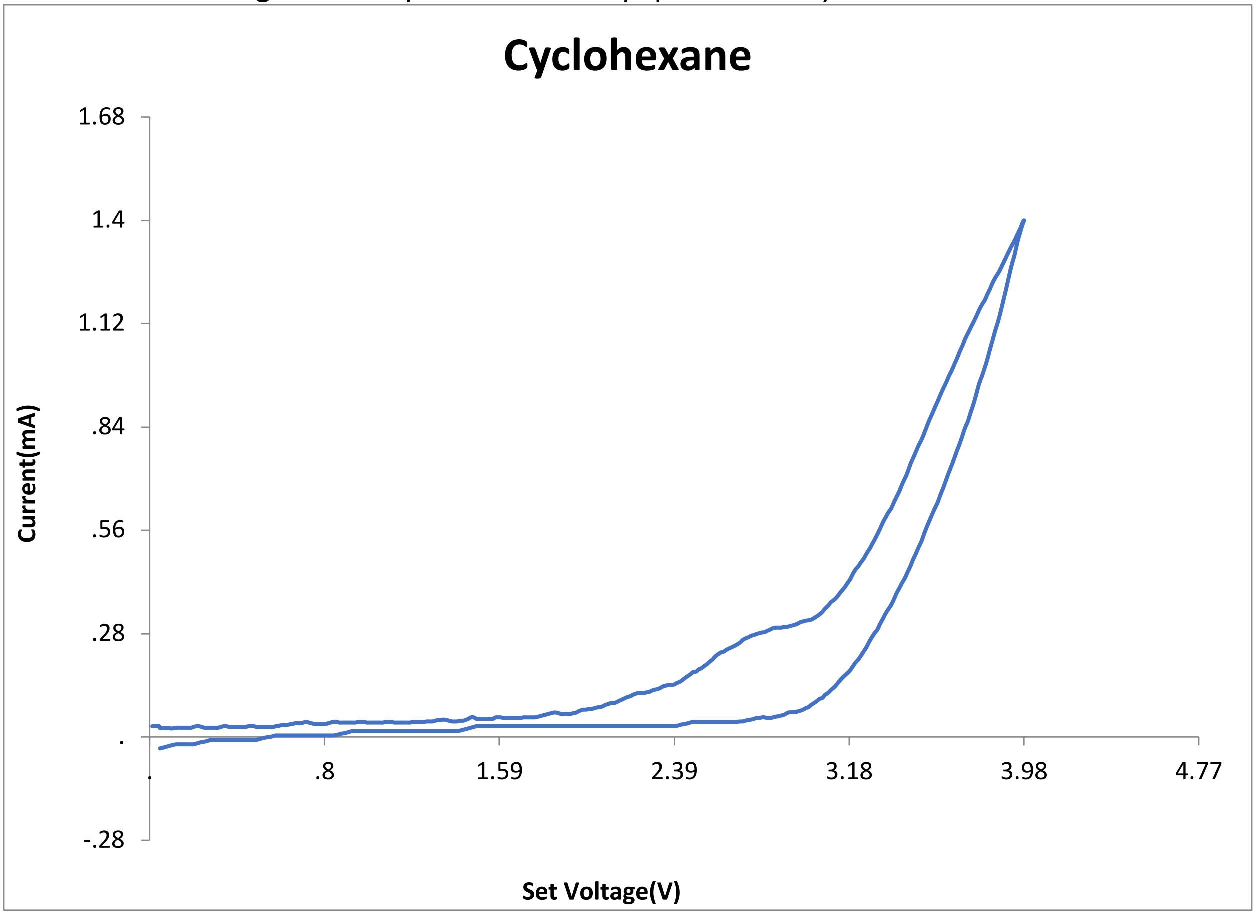

Figure S20. Cyclic voltammetry spectrum of cycloheptane.

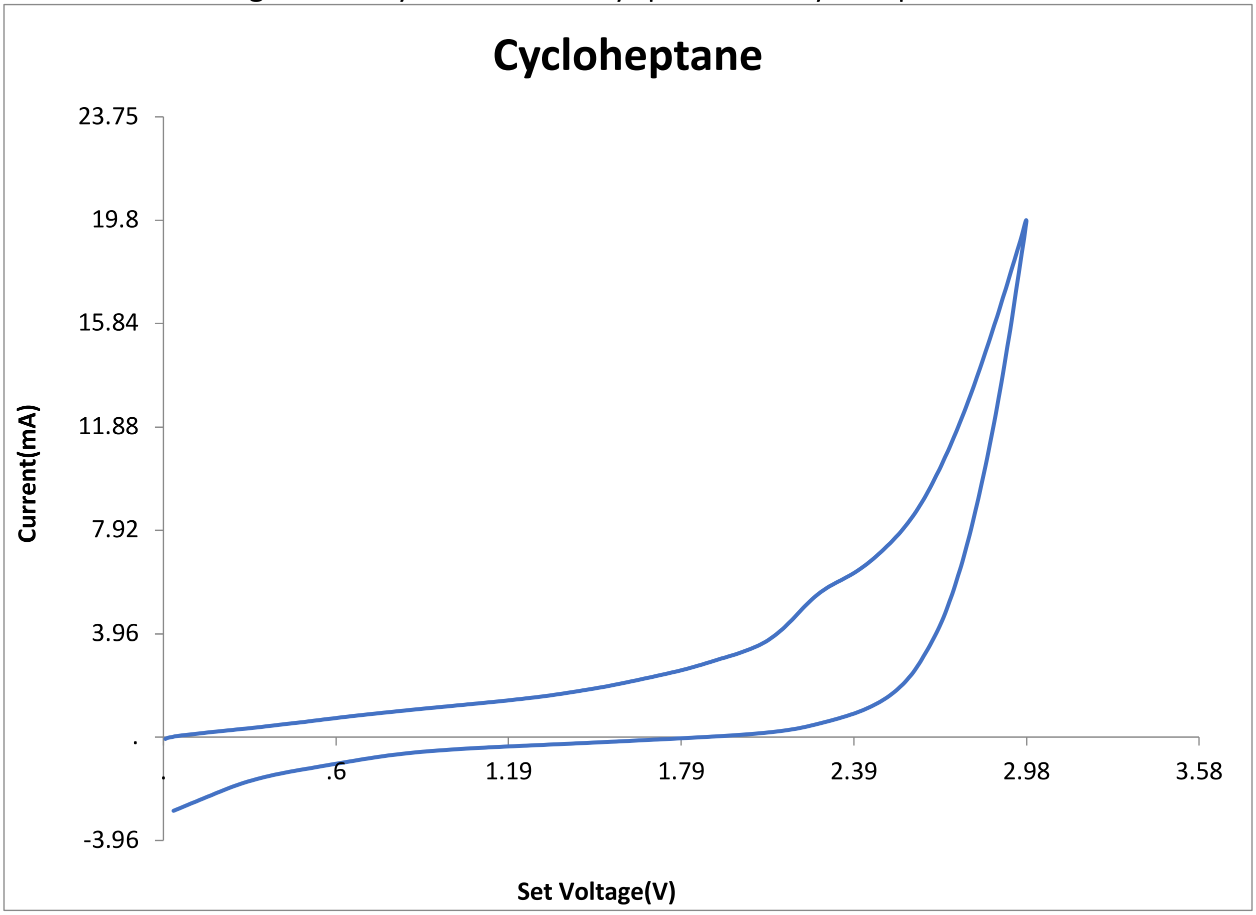

Figure S21. Cyclic voltammetry spectrum of cyclooctane.

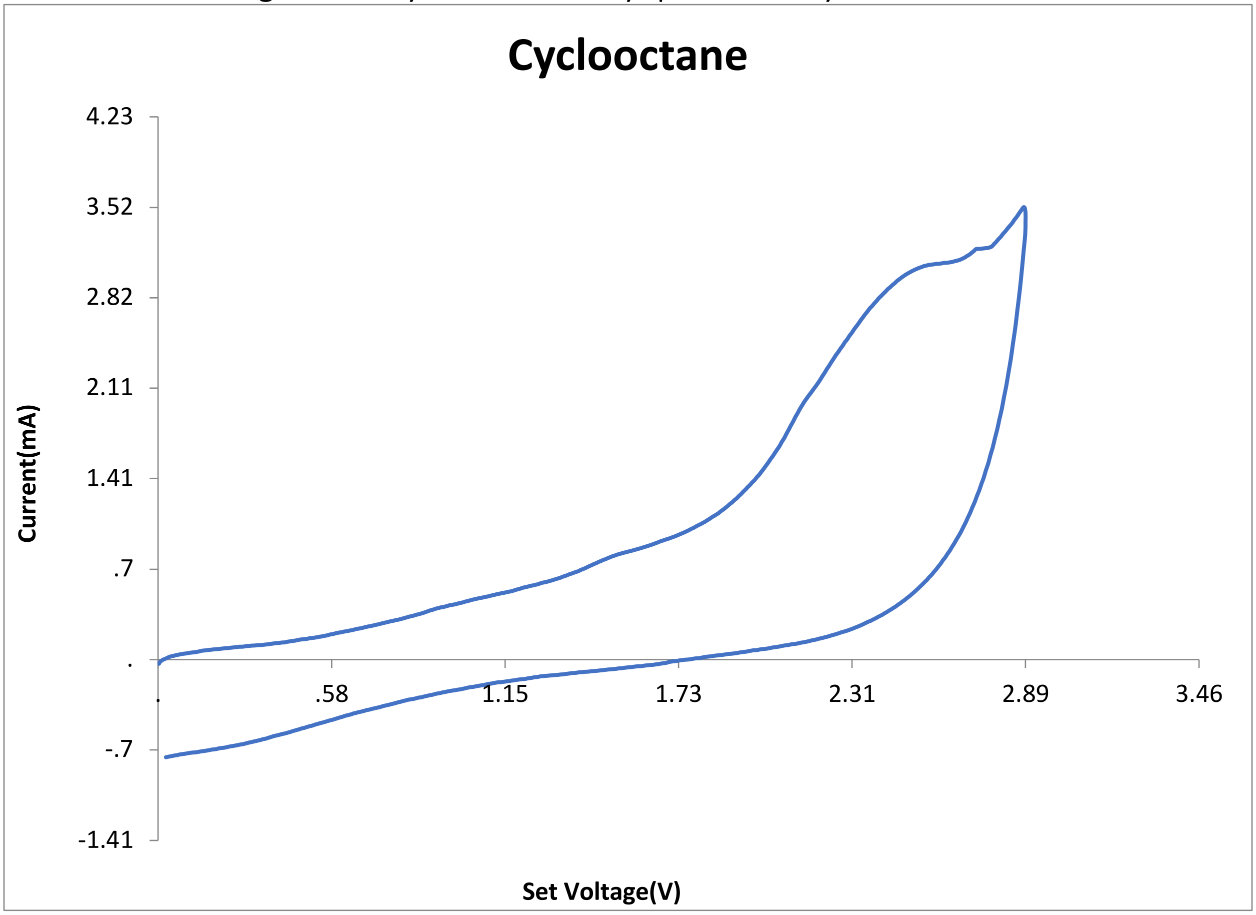

Figure S22. Cyclic voltammetry spectrum of cyclododecane.

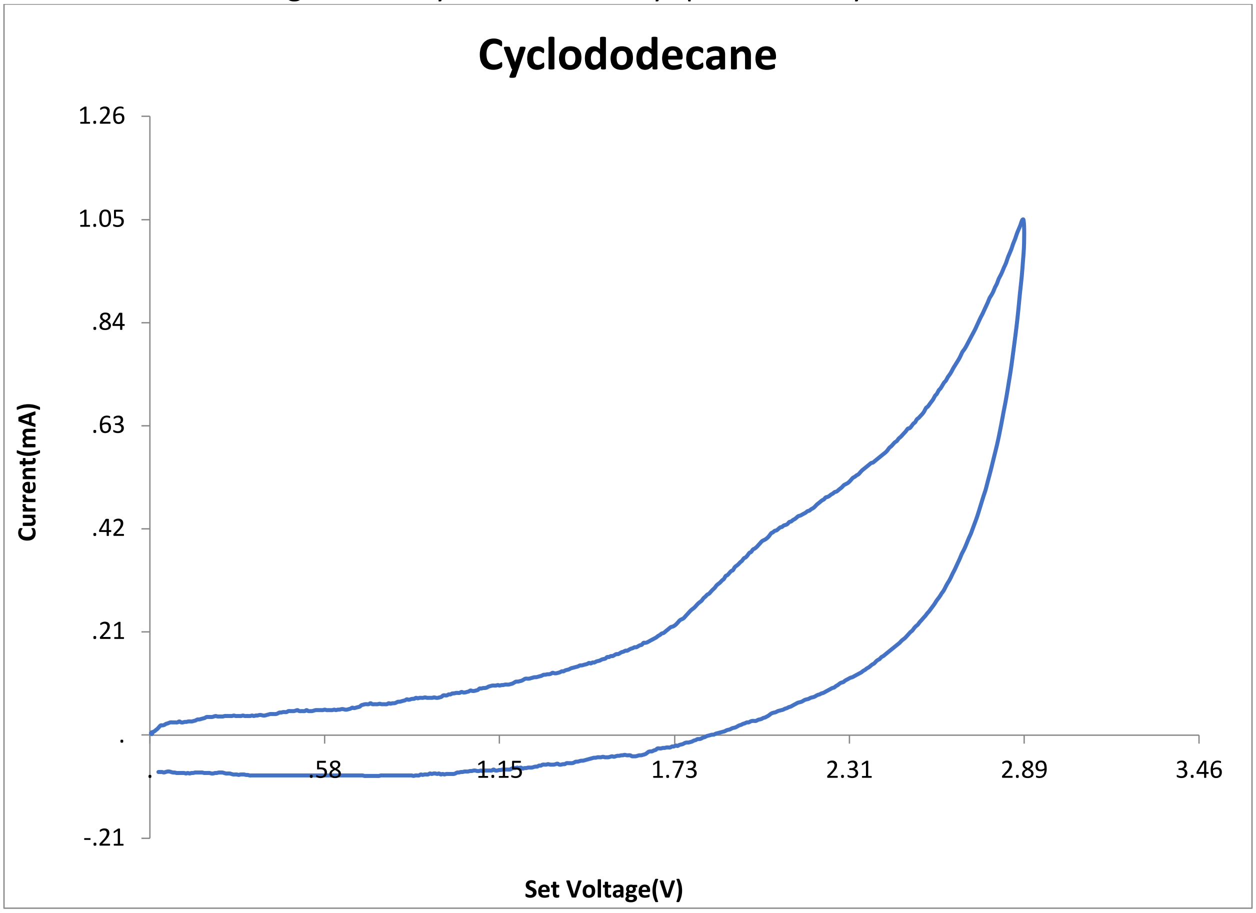

Figure S23. Cyclic voltammetry spectrum of *trans*-decalin.

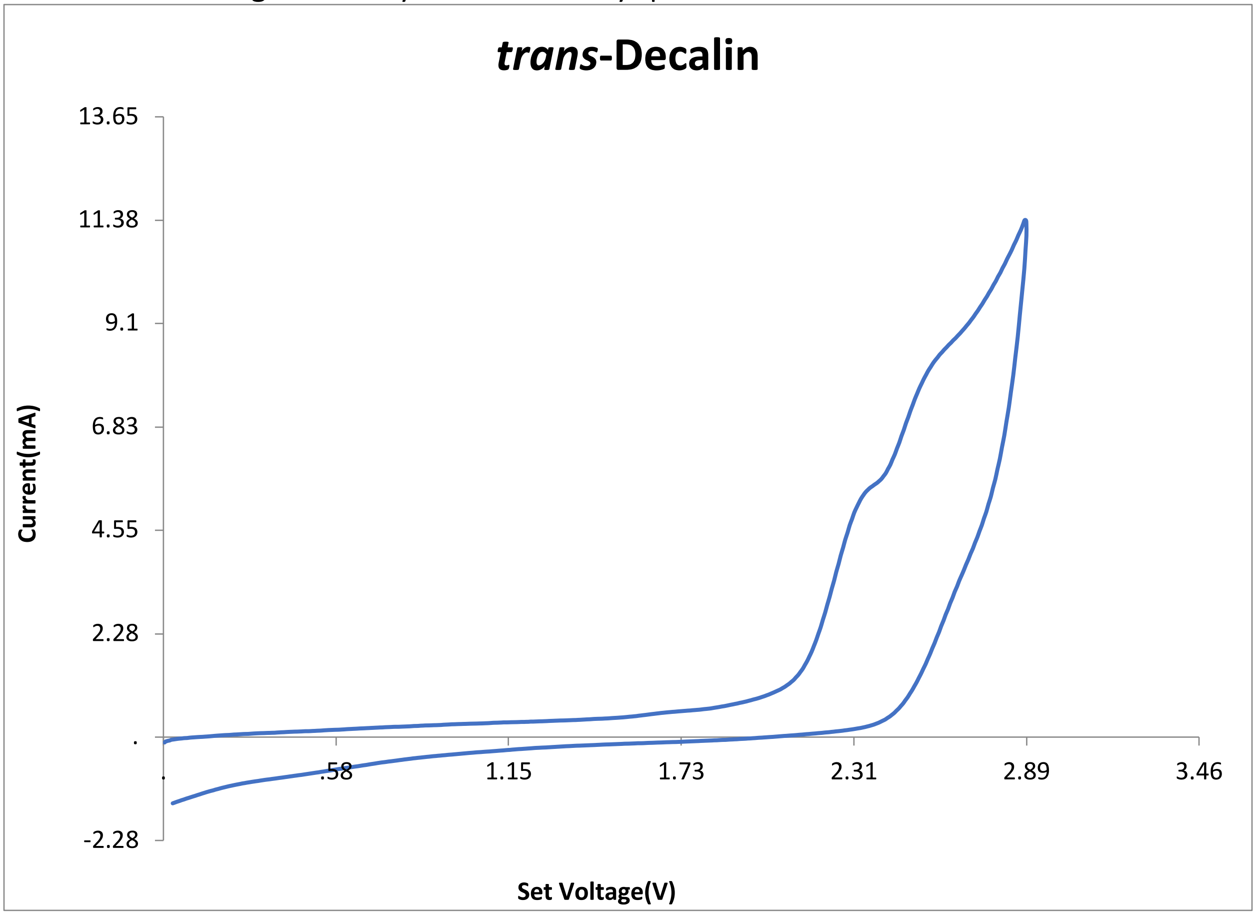

Figure S24. Cyclic voltammetry spectrum of norbornane.

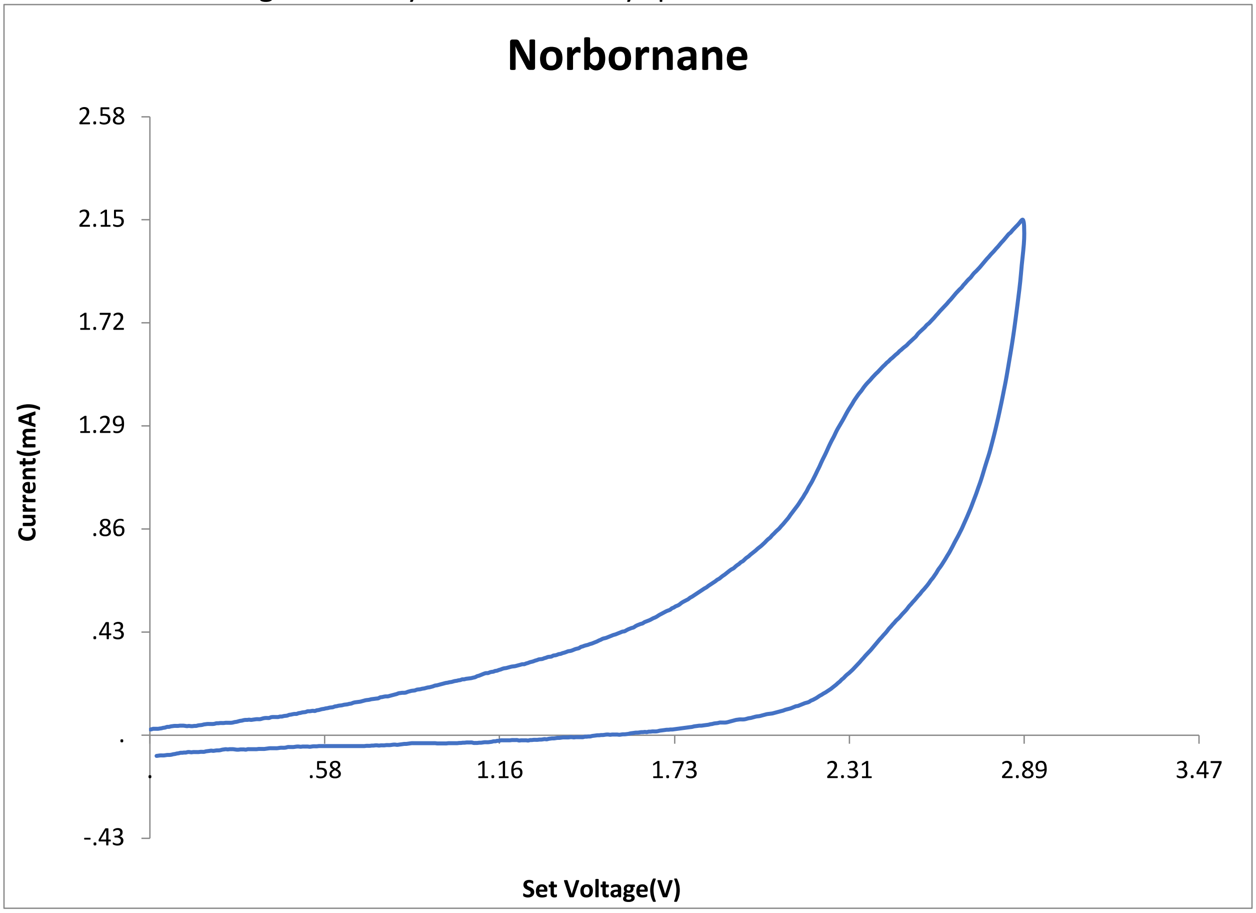

Figure S25. Cyclic voltammetry spectrum of methyl cyclohexane.

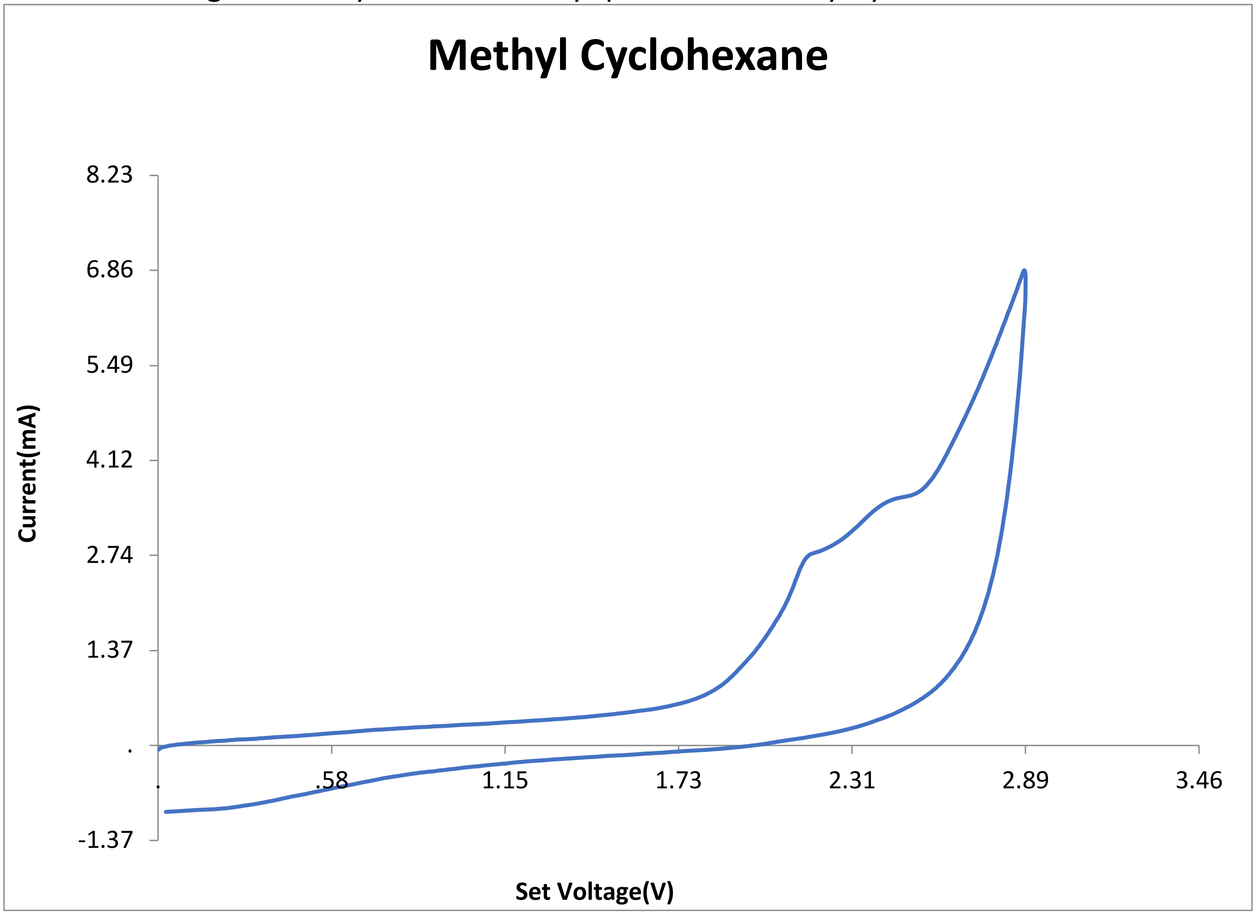

Figure S26. Cyclic voltammetry spectrum of *n*-hexane.

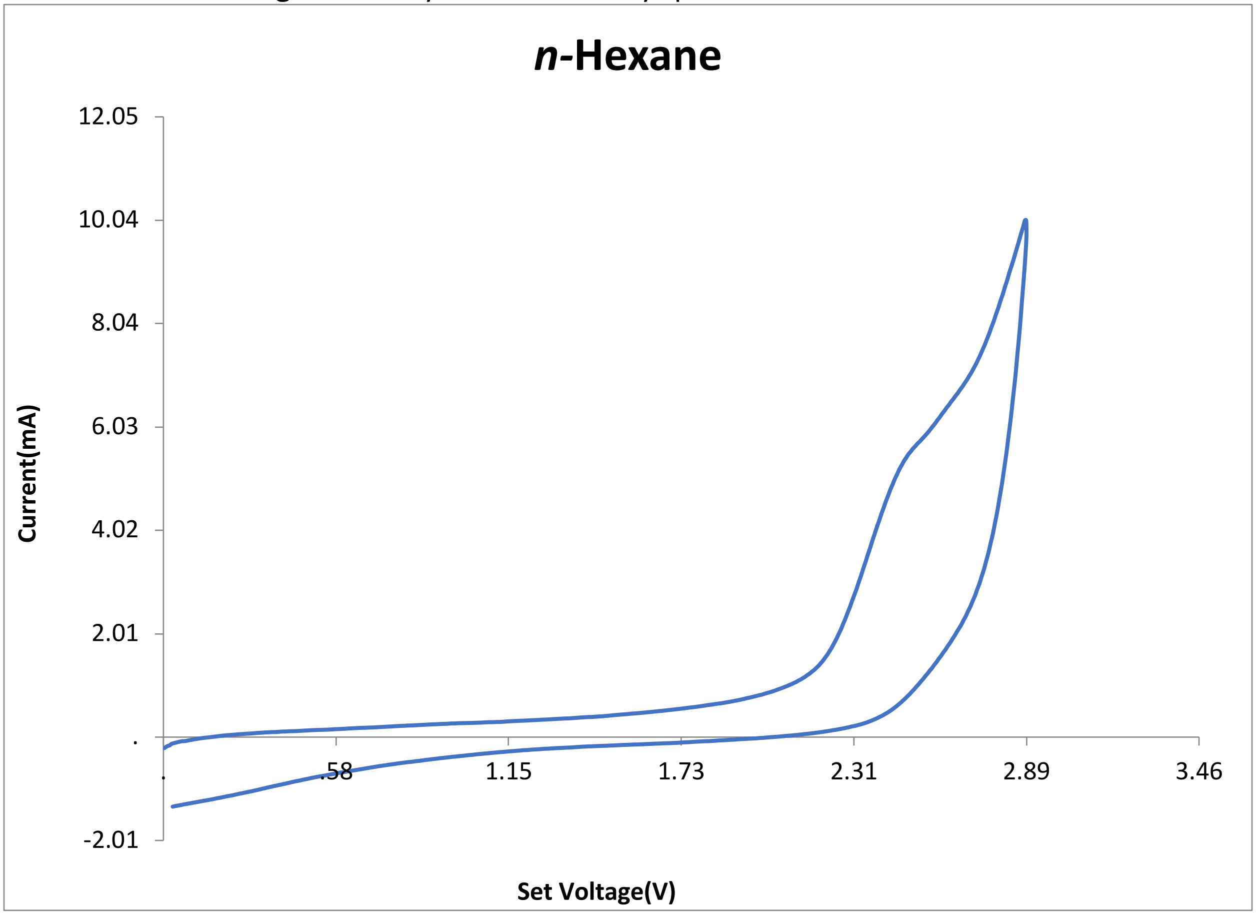

Figure S27. Cyclic voltammetry spectrum of 1-phenylhexane.

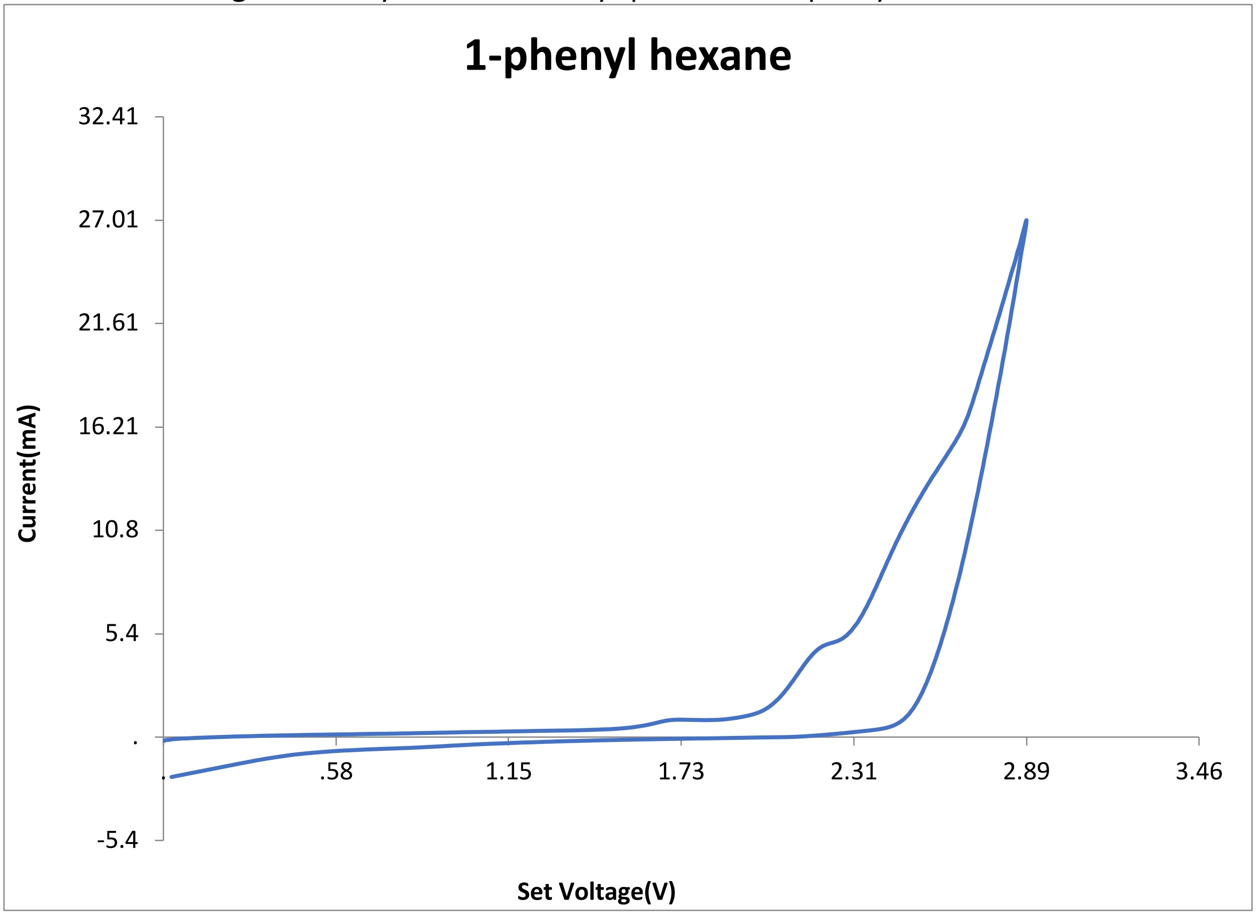

Figure S28. Cyclic voltammetry spectrum of sclareolide.

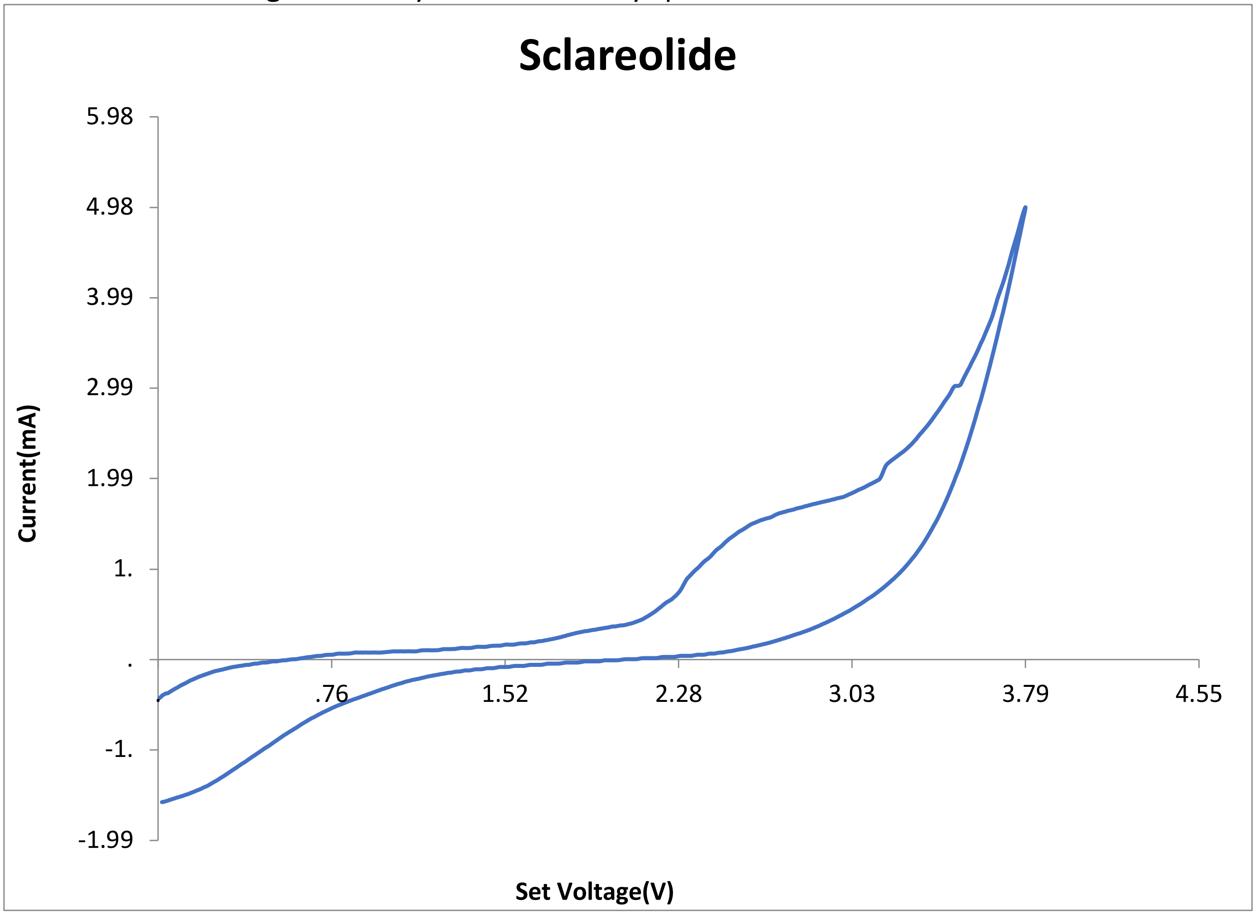

Figure S29. Cyclic voltammetry spectrum of adamantane.

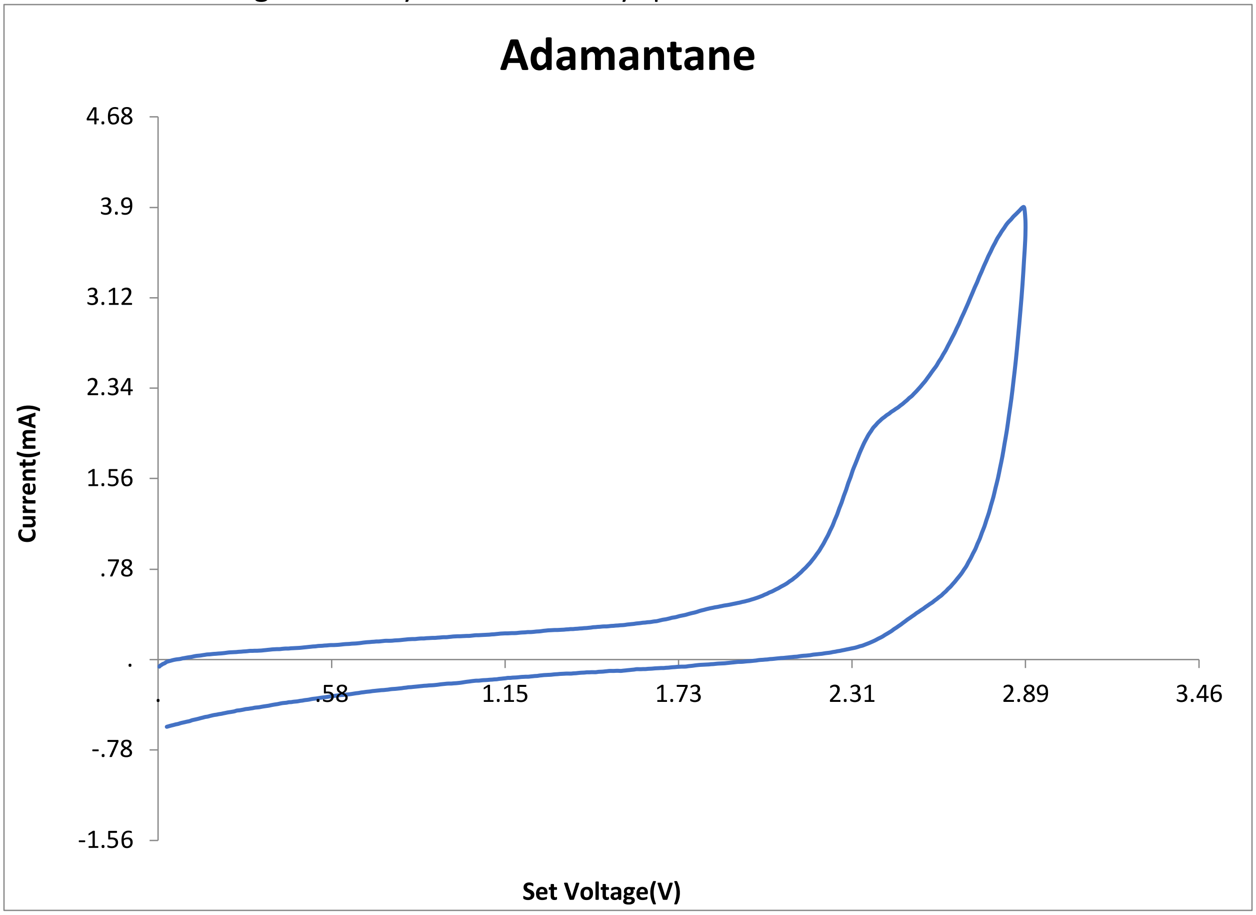

Figure S30. Cyclic voltammetry spectrum of *p*-(trifluoromethyl)anisole.

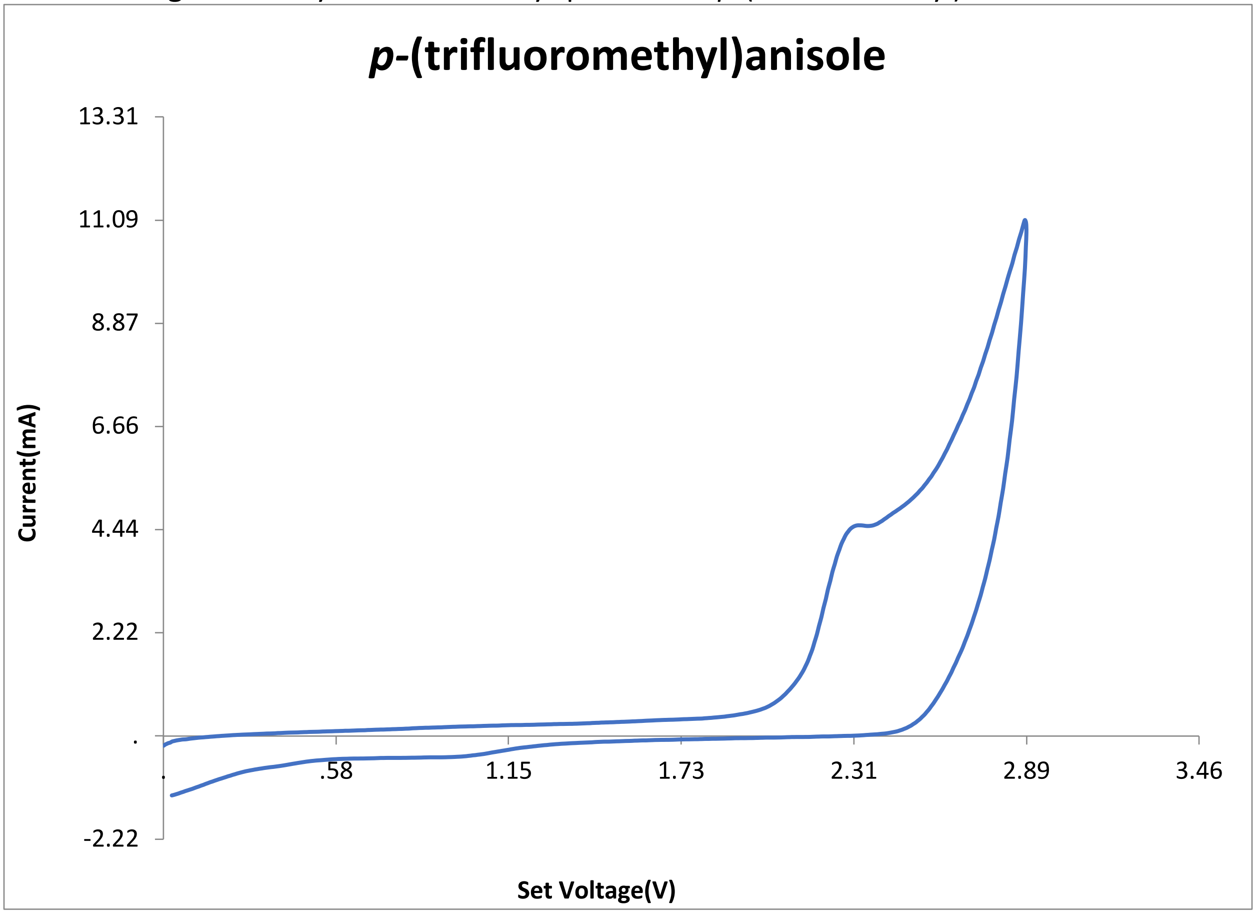

Figure S31. Cyclic voltammetry spectrum of riboflavin tetrabutyrate.

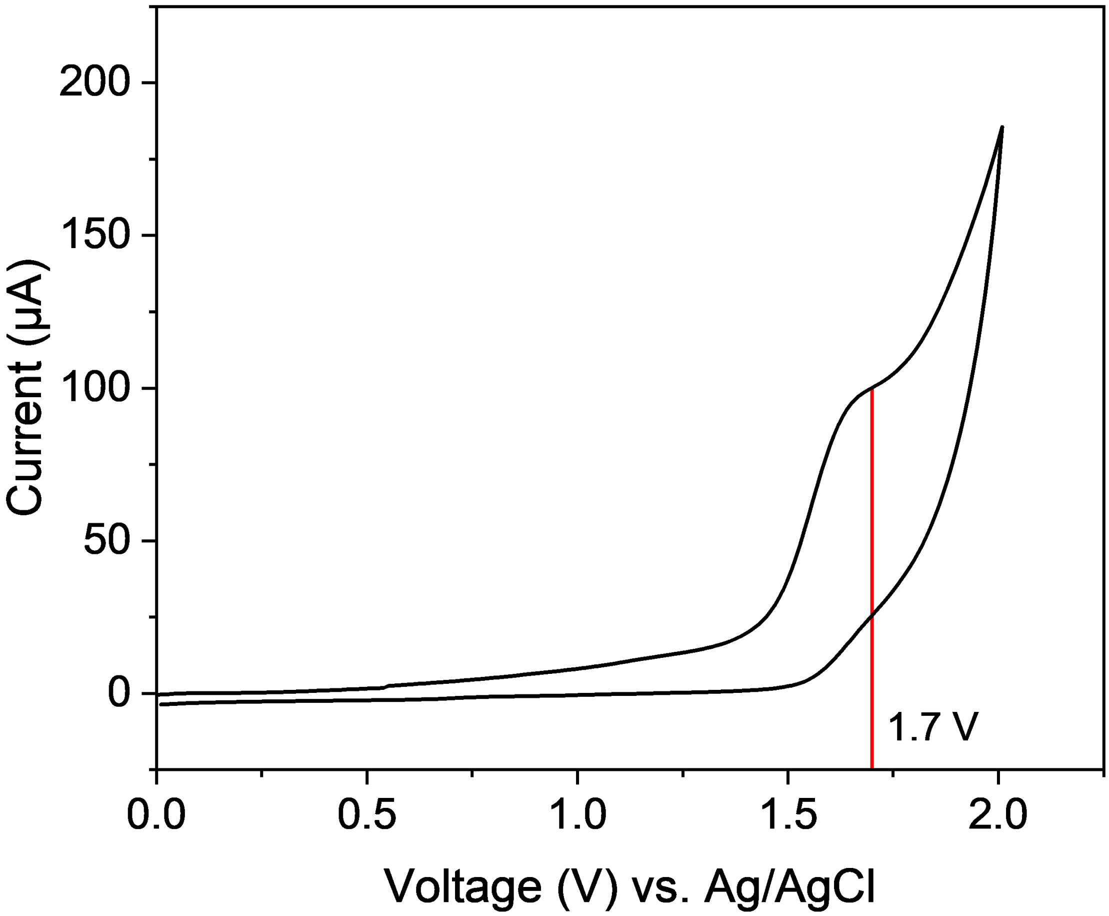

Figure S32. Cyclic voltammetry spectrum of riboflavin tetrabutyrate in the presence of $Sc(OTf)_3$.

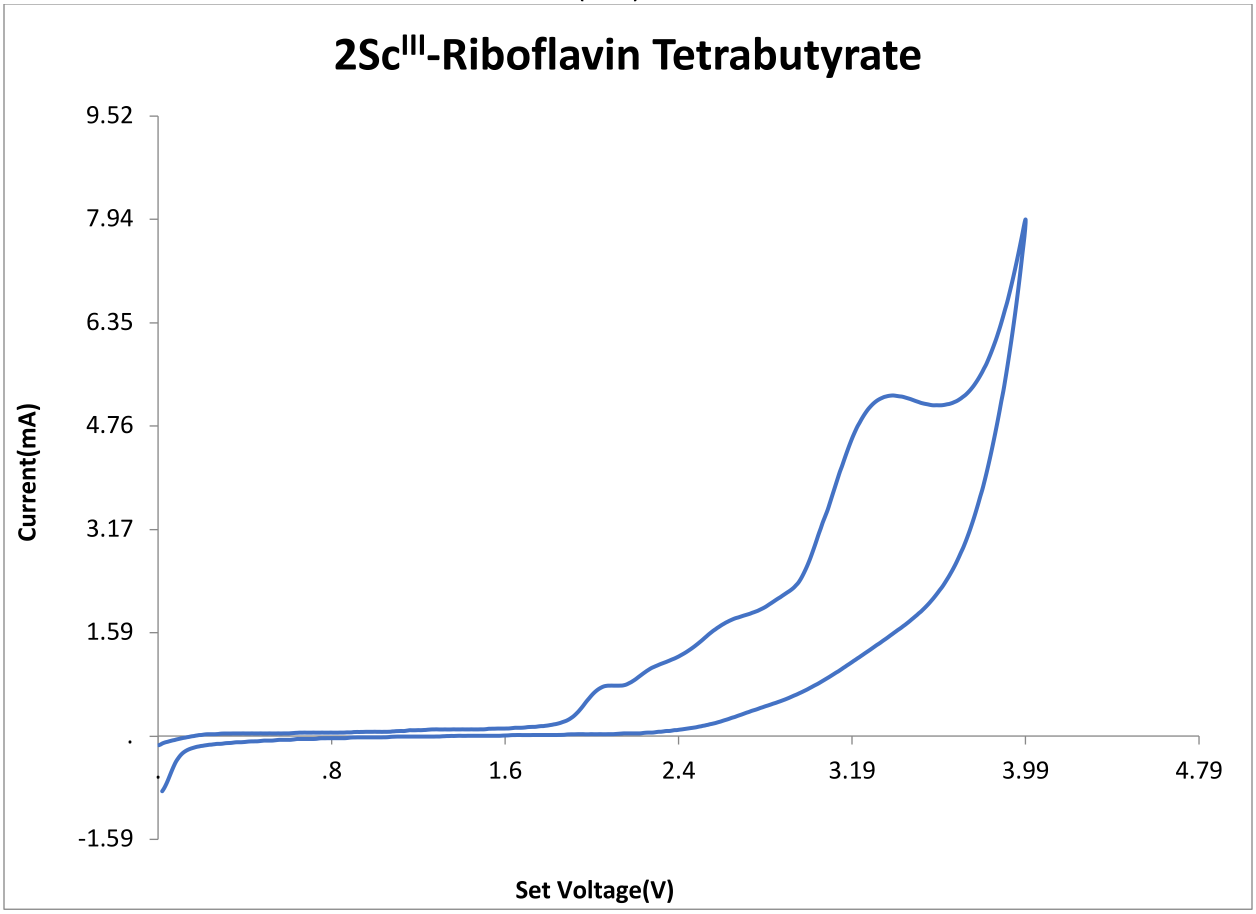

5. Calibration Curve Data and Control Reactions

Table S2. Generation of a standard calibration curve for *N*-cyclohexyl acetamide.

13.28	6912530	5801617	7300262	6671470	449098
6.64	4037056	3047341	5004761	4029719	565070
3.32	2005627	1906007	1954997	1955544	28759
1.66	1273622	796350	1485677	1185216	203842
0.83	879179	602255	972800	818078	111244
0.41	405460	222006	156262	261243	74564
0	0	0	0	0	0

Figure S33. Standard calibration curve generated for *N*-cyclohexylacetamide.

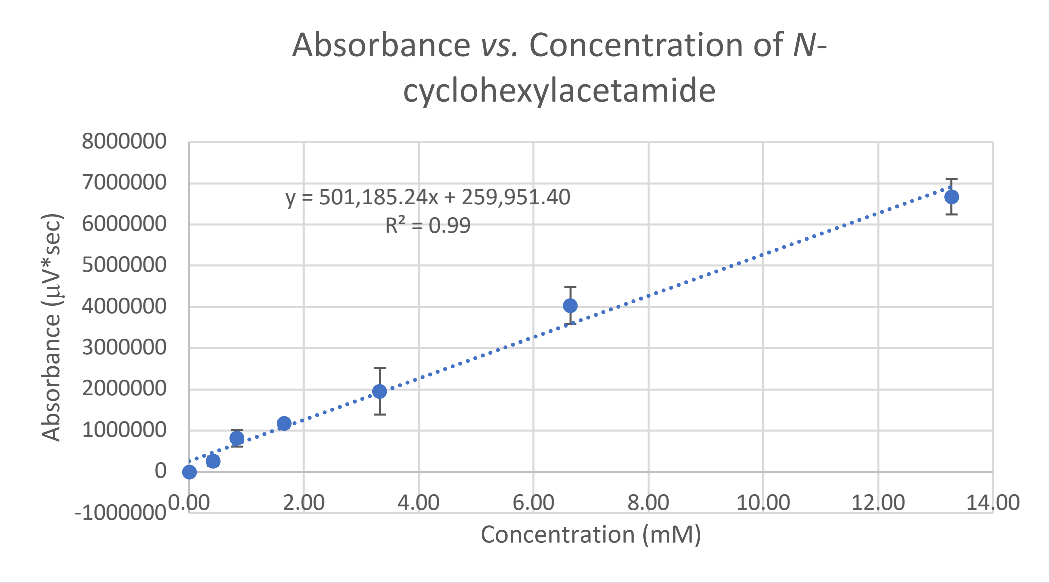

Figure S34. LC spectrum for Figure 2, Entry 1 (standard conditions).

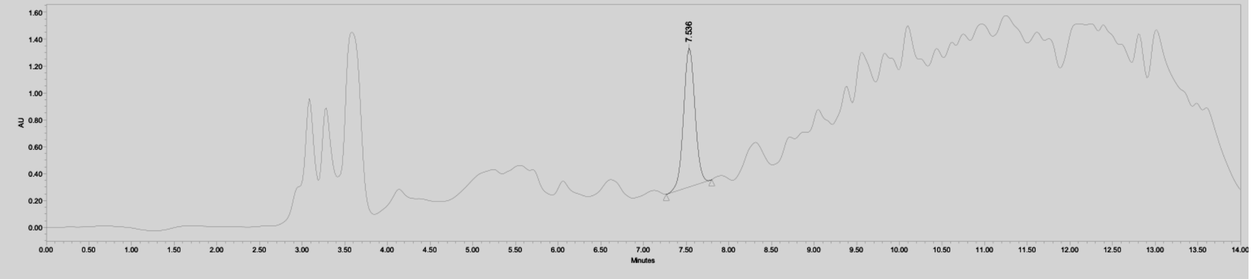

	Retention Time	Area
1	7.536	9593190

Figure S35. MS TIC for Table S3, Entry 1 (standard conditions).

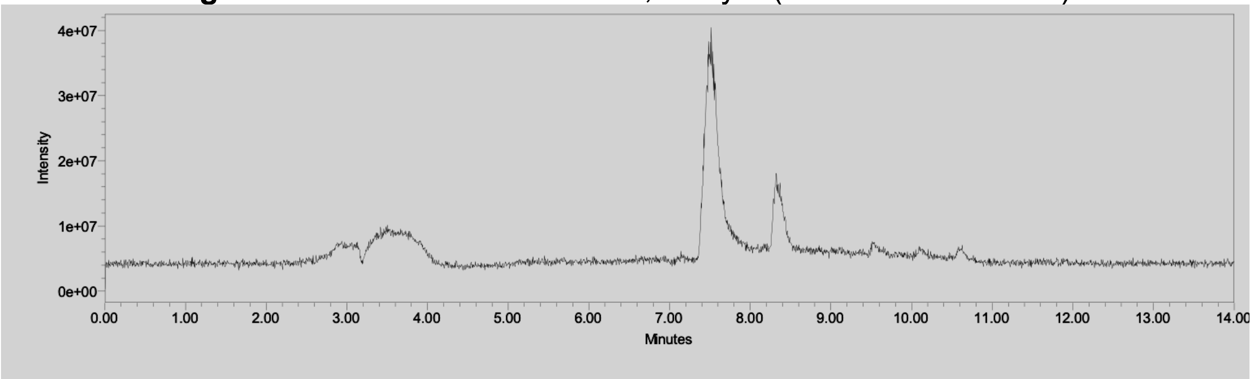

Figure S36. Extracted *m/z* for Table S3, Entry 1 (standard conditions).

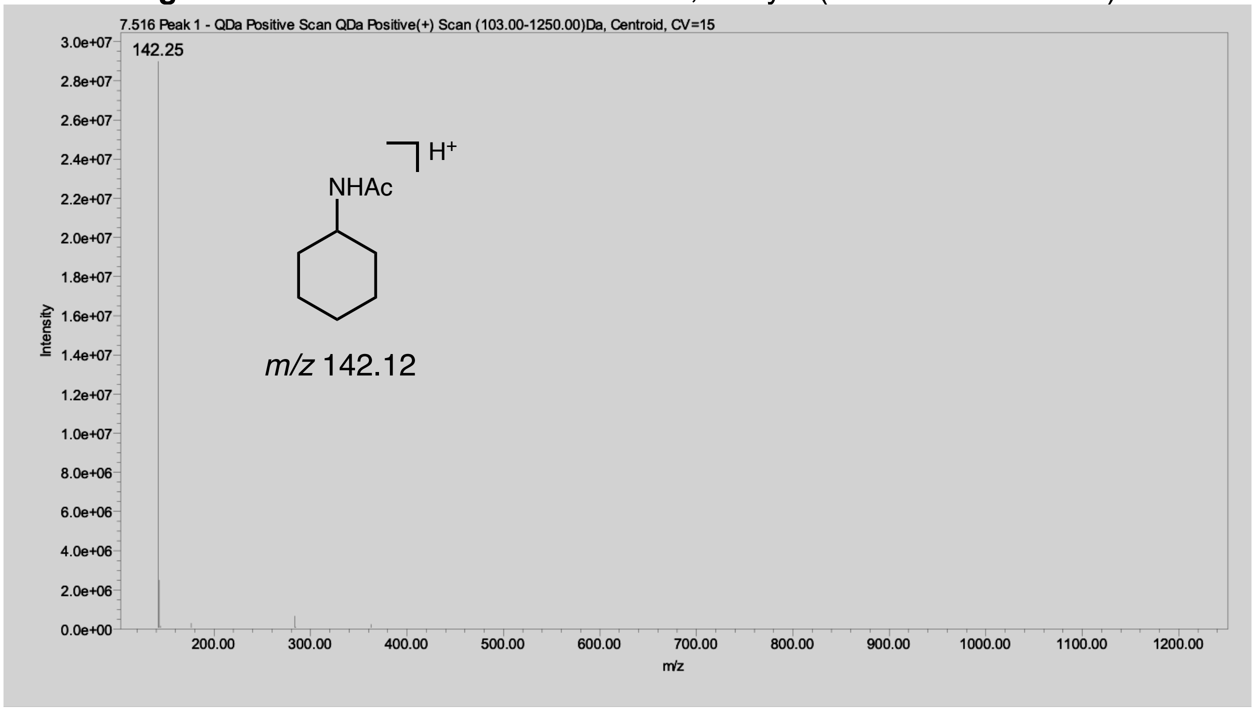

Figure S37. LC spectrum for Table S3, Entry 2 (without Cl_3CCN).

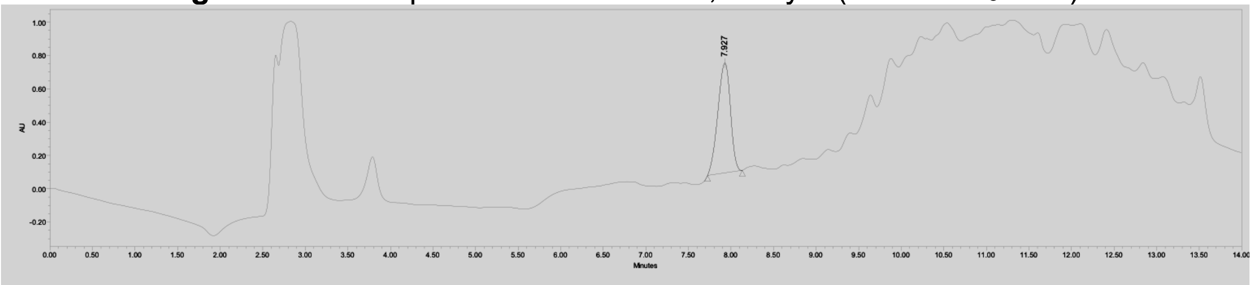

	Retention Time	Area
1	7.927	6778824

Figure S38. MS TIC for Table S3, Entry 2 (without Cl_3CCN).

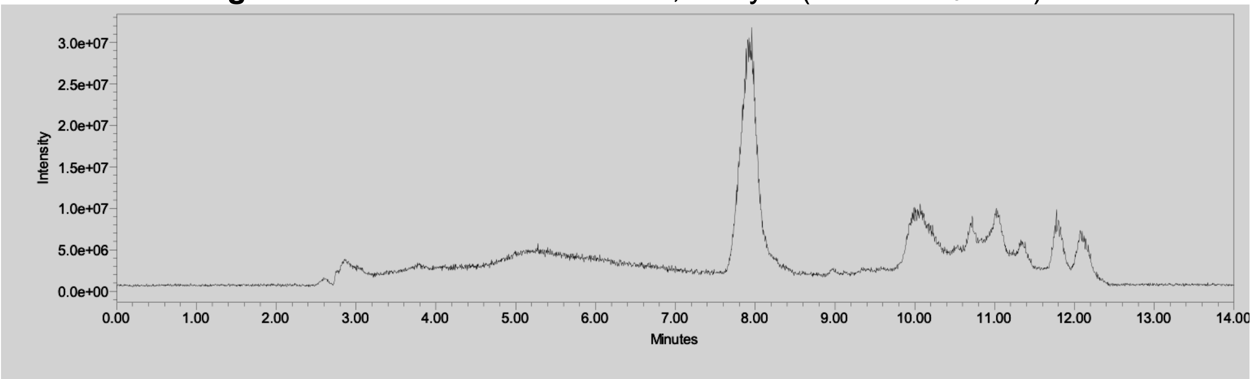

Figure S39. Extracted *m/z* for Table S3, Entry 2 (without Cl_3CCN).

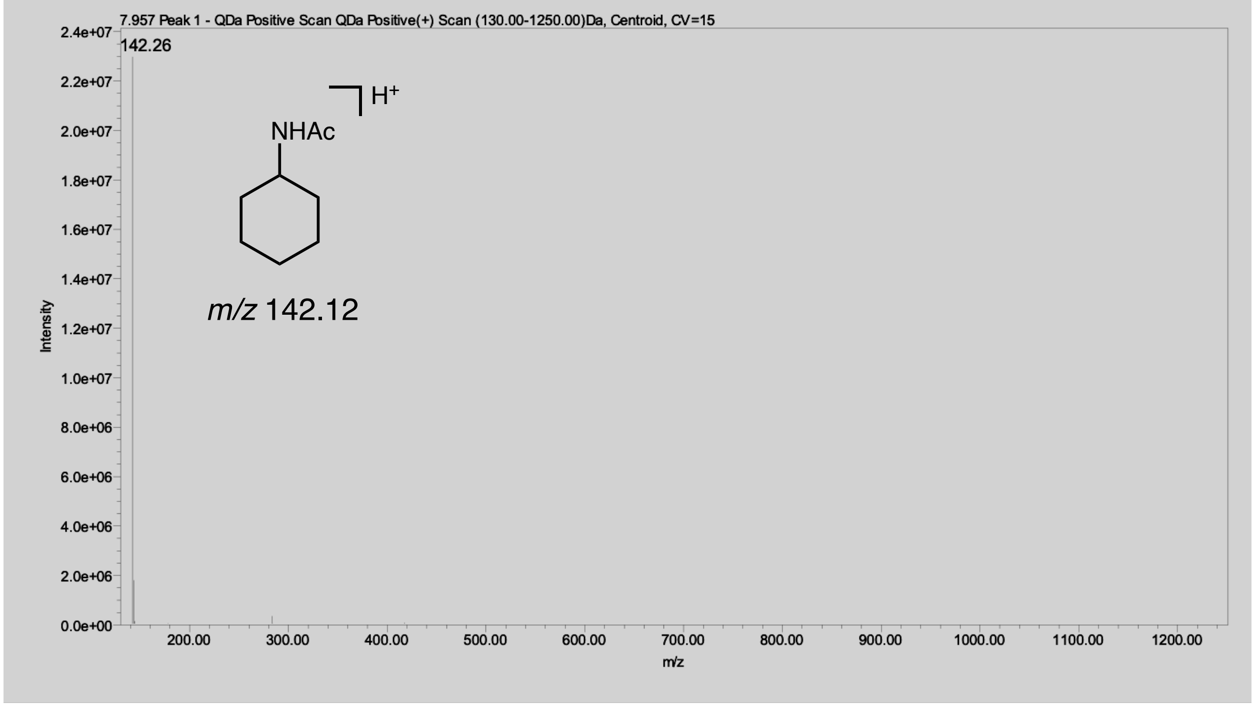

Figure S40. LC spectrum for Table S3, Entry 3 (without $Sc(OTf)_3$).

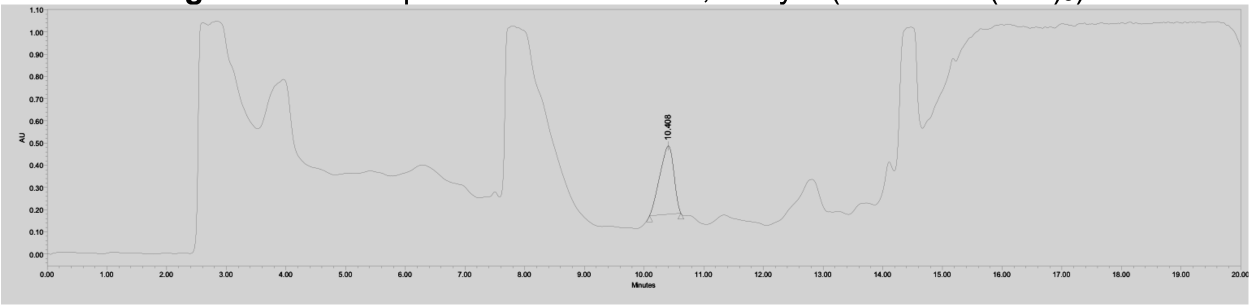

	Retention Time	Area
1	10.408	4982406

Figure S41. MS TIC for Table S3, Entry 3 (without $Sc(OTf)_3$).

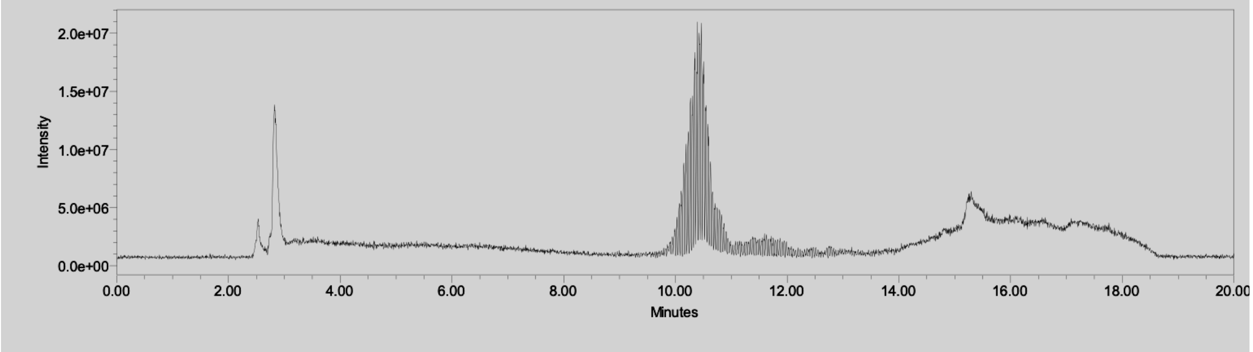

Figure S42. Extracted *m/z* for Table S3, Entry 3 (without $Sc(OTf)_3$).

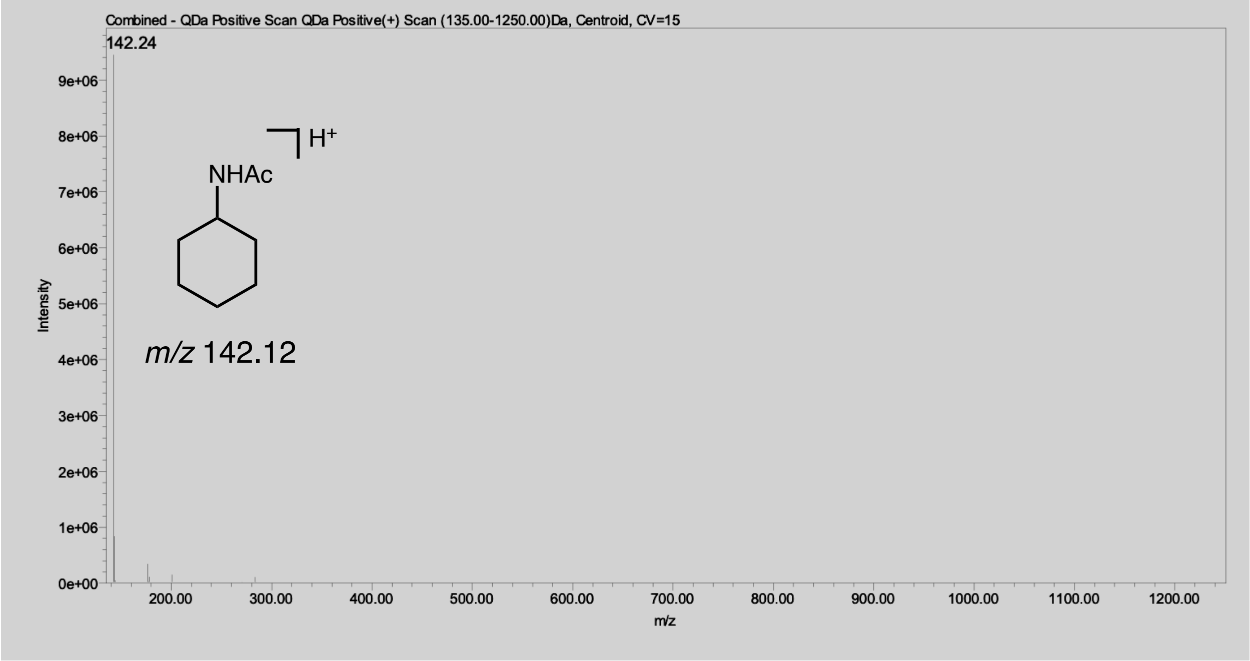

Table S3. Control Reactions.

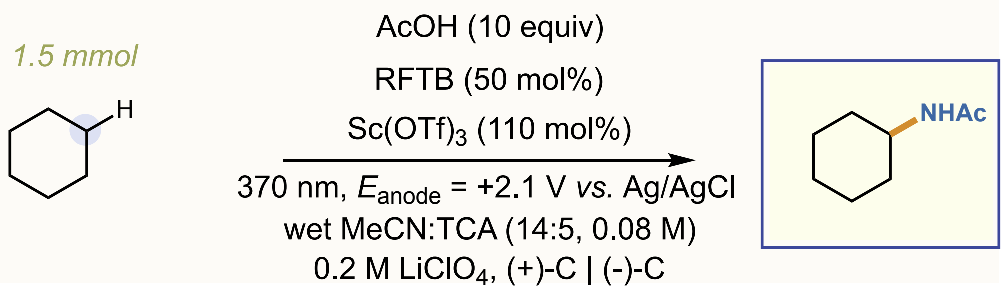

Entry	Deviation from standard conditions	Yield %
1	***None***	**33%**
2	No TCA	22%
3	No $Sc(OTf)_3$	16%
4	No RFTB	0
5	No constant anodic potential	0
6	No 370 nm light	0

Kinetic Isotope Experiment

A competitive kinetic isotope experiment was carried out under standard alkane reaction conditions (**S3**) using 0.75 mmol of cyclohexane and 0.75 mmol d_{12}-cyclohexane in the same reaction vial. The KIE was determined by comparing the total ion count of the two products by LCMS analysis of the crude reaction mixture. K_H=128303467 and K_D=103456329 giving a KIE of K_H/K_D=1.24.

Figure S43. Extracted m/z for the kinetic isotope experiment reaction.

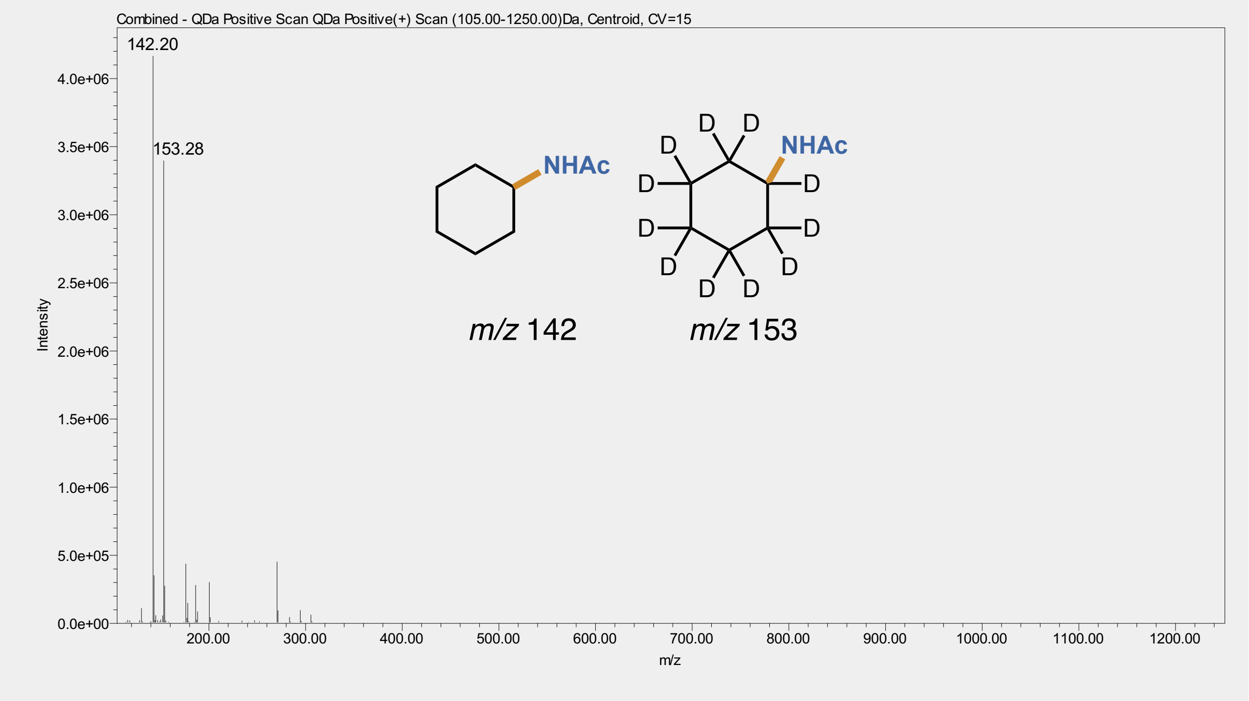

Figure S44. TIC spectrum obtained after extracting m/z 142 (**16b**).

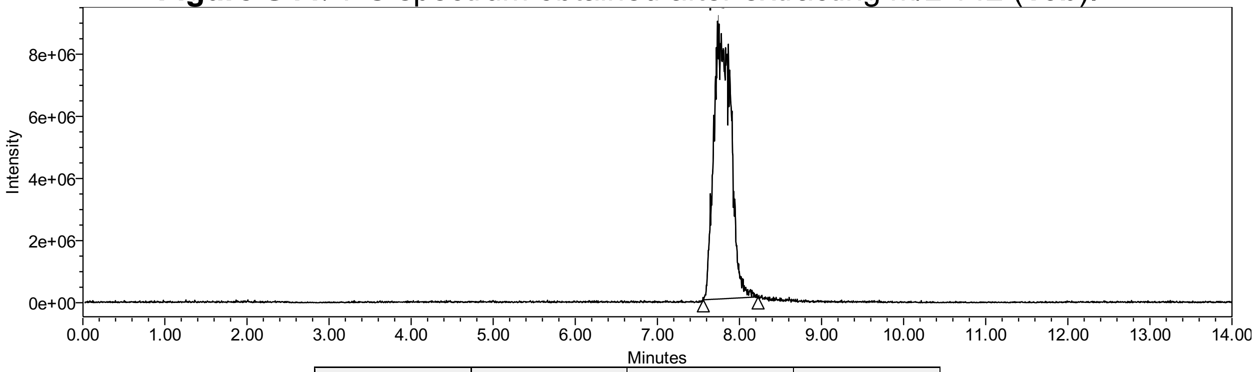

	Name	Retention Time	Area
1	*m/z* 142	7.734	128303467

Figure S45. TIC spectrum obtained after extracting m/z 153 (d_{11}-**16b**).

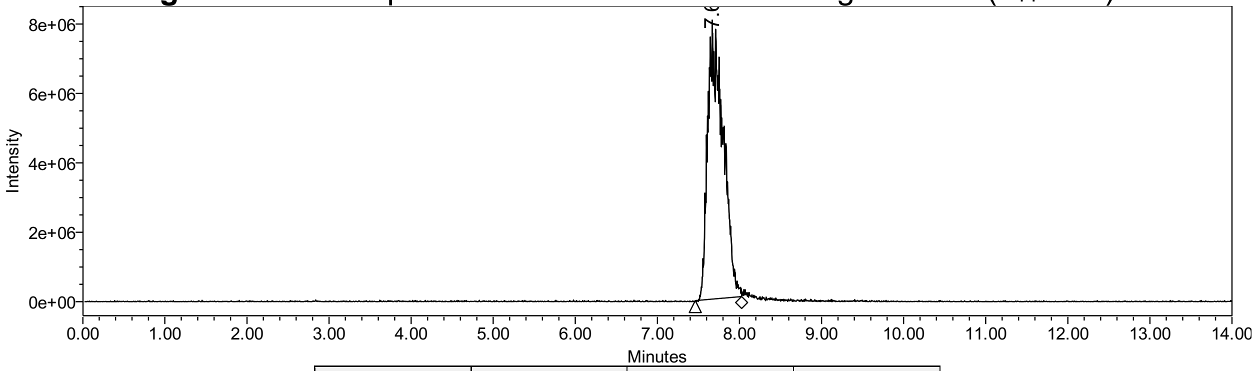

	Name	Retention Time	Area
1	*m/z* 153	7.671	103456329

Figure S46. Catalyst-derived m/z observed in a crude reaction mixture and tentative assignments of their identity.

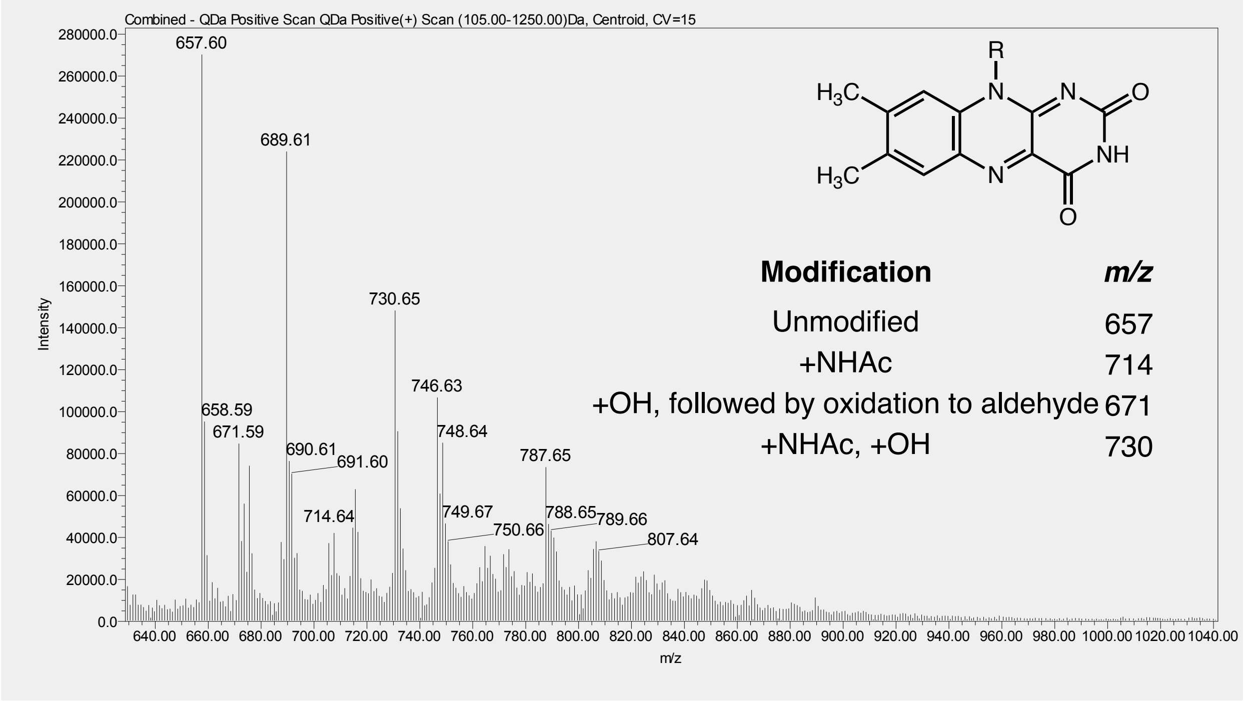

Methylene Blue Quenching for [•OH] determination

Methylene blue (λ_{max} = 656 nm) undergoes quenching in the presence of hydroxyl radicals to yield a colorless species.[4,5] We first prepared a standard curve of methylene blue in MeCN, which was then used to quantify hydroxyl radical concentration under a number of reaction conditions. For hydroxyl radical concentration determination, a 1.0 mL aliquot of the indicated reaction mixture was diluted with 0.975 mL of MeCN and 25 μL of methylene blue stock solution (1.0 mM in MeCN; diluted to 25 μM). This solution was vortexed briefly and the absorbance at 656 nm was immediately measured. The concentration of unquenched methylene blue was subtracted from the initial concentration and multiplied by two to account for dilution to provide the concentration of hydroxyl radicals.

Figure S47. Methylene blue absorbance spectra from 0-25 μM.

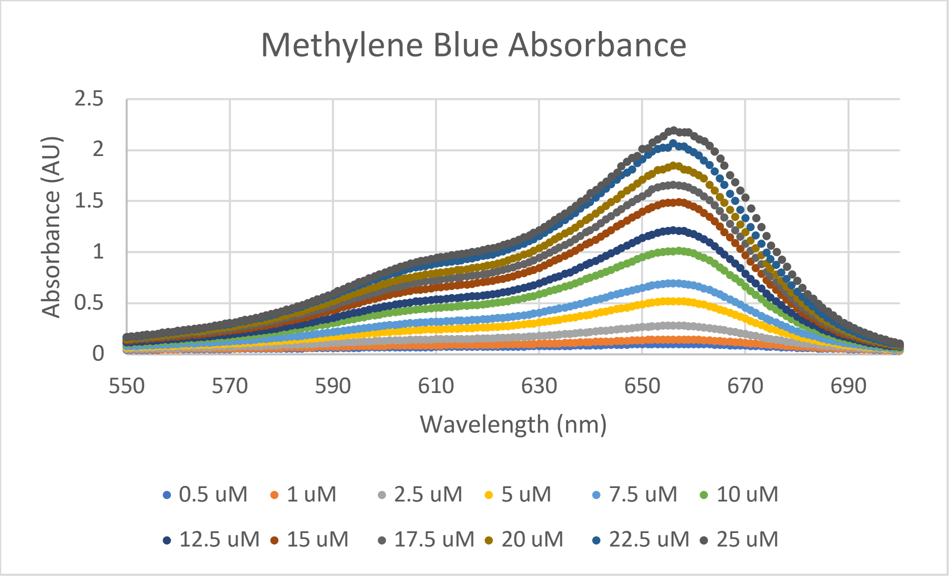

Figure S48. Standard curve for methylene blue concentration.

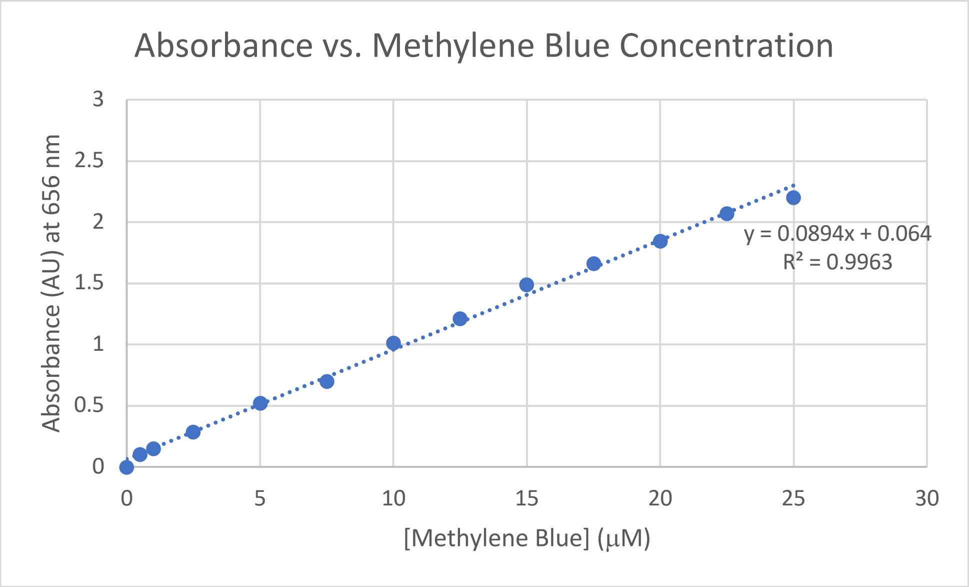

Table S4. Determination of hydroxyl radical concentration under various reaction conditions.

Conditions	Absorbance	[MB] (μM)	[•OH] (μM)
RFTB, 2.1 V	2.245	24.40	1.21
$2Sc^{III}$-RFTB, 2.1 V	2.232	24.25	1.50
$2Sc^{III}$-RFTB, 2.1 V, C_6H_{12}	1.403	14.98	20.04
$2Sc^{III}$-RFTB, 2.1 V, C_6H_{12}, 370 nm	1.686	18.14	13.71

6. Transient Absorption Spectroelectrochemistry

General Materials and Methods

Solutions of RFTB and 2Sc:RFTB were prepared in acetonitrile (VWR) at a concentration of 0.1 mM of RFTB; for spectroelectrochemical measurements 0.1 M NBu_4PF_6 was also added to solutions to act as a supporting electrolyte. Steady state spectroelectrochemical measurements were performed using Cary 60 UV/Vis spectrophotometer (Agilent technologies) and the three electrode bespoke cell mentioned in Section 4 above. From the cyclic voltammetry measurements (see Section 4 above), an oxidation potential of 1.7 V was chosen to drive the oxidation of RFTB and was applied for 10 minutes before taking a spectrum. Electrochemical measurements were performed using a PalmSens EmStat3 potentiostat.

Transient Absorption spectroscopy measurements were performed on a system described previously,[3] with the parameters used for these measurements given below. Samples were placed in a 1 mm thick cuvette and photoexcited at their absorption maximum (450 nm for RFTB and 390 nm for RFTB•+ and 2Sc:RFTB). Samples were translated in the plane of the probe focus to ensure that a fresh sample was interrogated for each spectra. The spectroelectrochemical setup used for the steady-state measurements was also utilized for the ultrafast transient absorption spectroelectrochemistry measurements. The transient absorption spectra were fit with a global model using the Glotaran software.[6,7] The lifetimes were extracted using a sequential model ($A \overset{\tau_1}{\rightarrow} B \overset{\tau_2}{\rightarrow} C$) with two lifetimes. The fitting of the transient absorption spectra also accounted for the dispersion, with a third order polynomial correction, and the instrument response of the system measured to be 100 fs. The resultant evolutionary associated difference spectra are shown in **Figure S50**, with the lifetimes given in **Table S5**. The residuals which were used as a measure of the quality of the fit, are also shown in **Figure S50**.

Table S5. Lifetimes extracted from the global fitting of the transient absorption spectra. Errors are shown to twice the standard error.

Sample	τ_1 (ps)	τ_2 (ns)
RFTB	76.07 ± 2.12	>> 3
RFTB•+	123.24 ± 61.39	>> 3
2Sc:RFTB	25.05 ± 15.12	>> 3

Figure S49. Transient absorption spectroscopy of RFTB (λ = 450 nm).

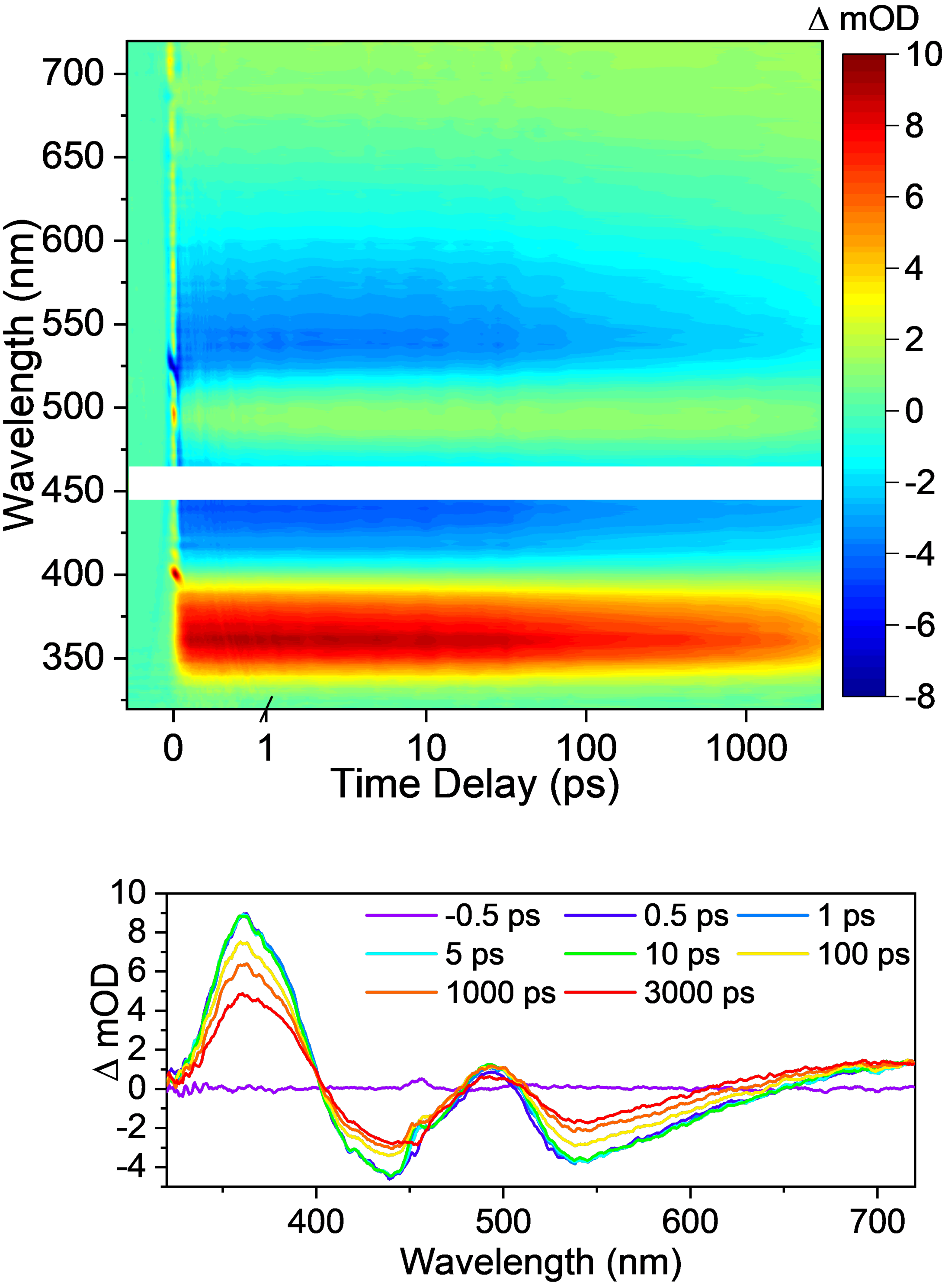

Figure S50. Transient absorption spectroelectrochemistry of RFTB•+ (+1.7 V *vs* Ag/AgCl, λ = 390 nm).

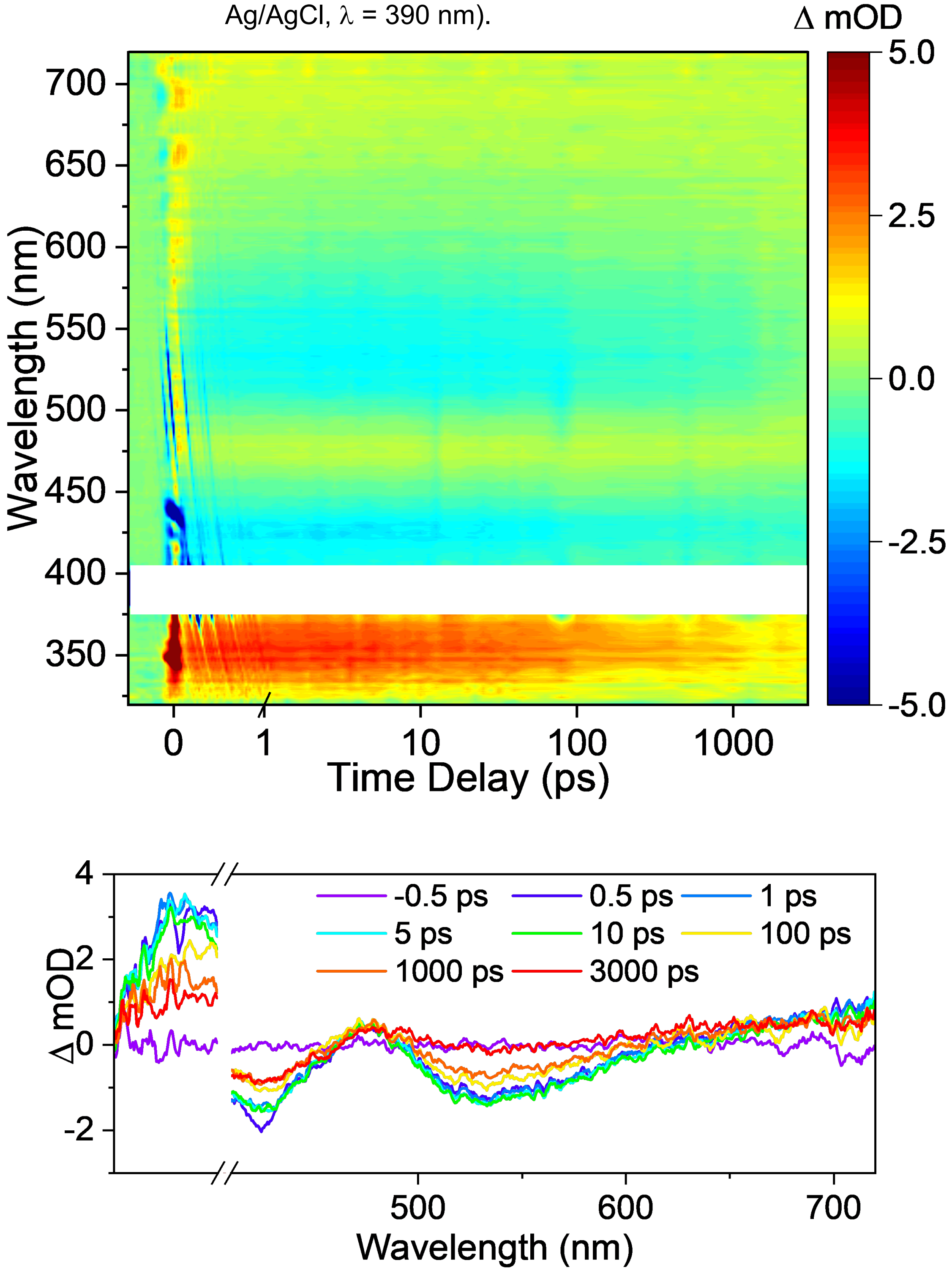

Figure S51. Transient absorption spectroscopy of 2Sc-RFTB (λ = 390 nm).

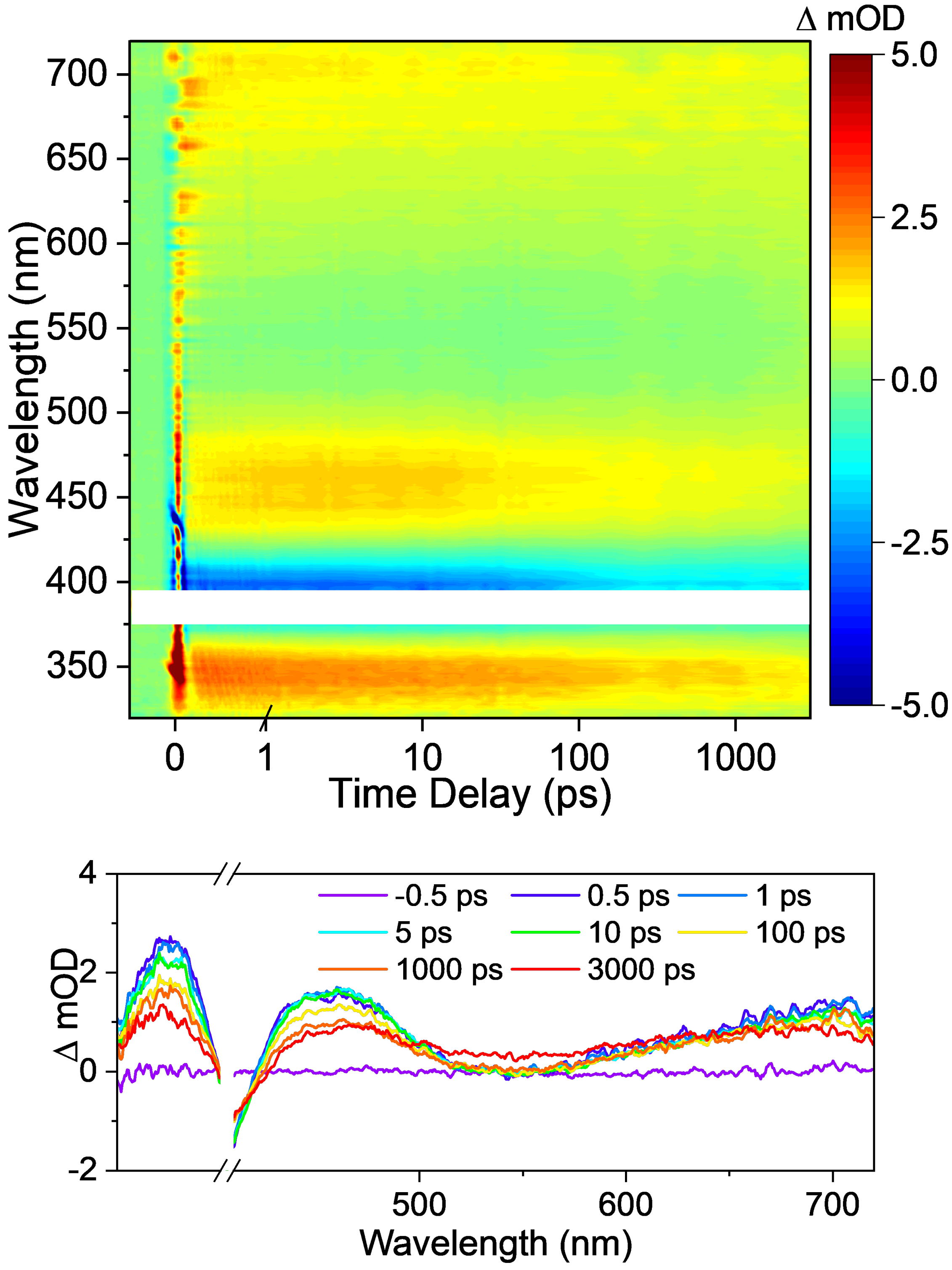

Figure S52. False color heat maps showing the residuals of the global fitting of a) RFTB•+ photoexcited at 390 nm, b) 2Sc:RFTB photoexcited at 390 nm c) RFTB photoexcited at 450 nm. Evolutionary associated difference spectra extracted from the global fitting of d) RFTB•+ photoexcited at 390 nm, e) 2Sc:RFTB photoexcited at 390 nm f) RFTB photoexcited at 450 nm.

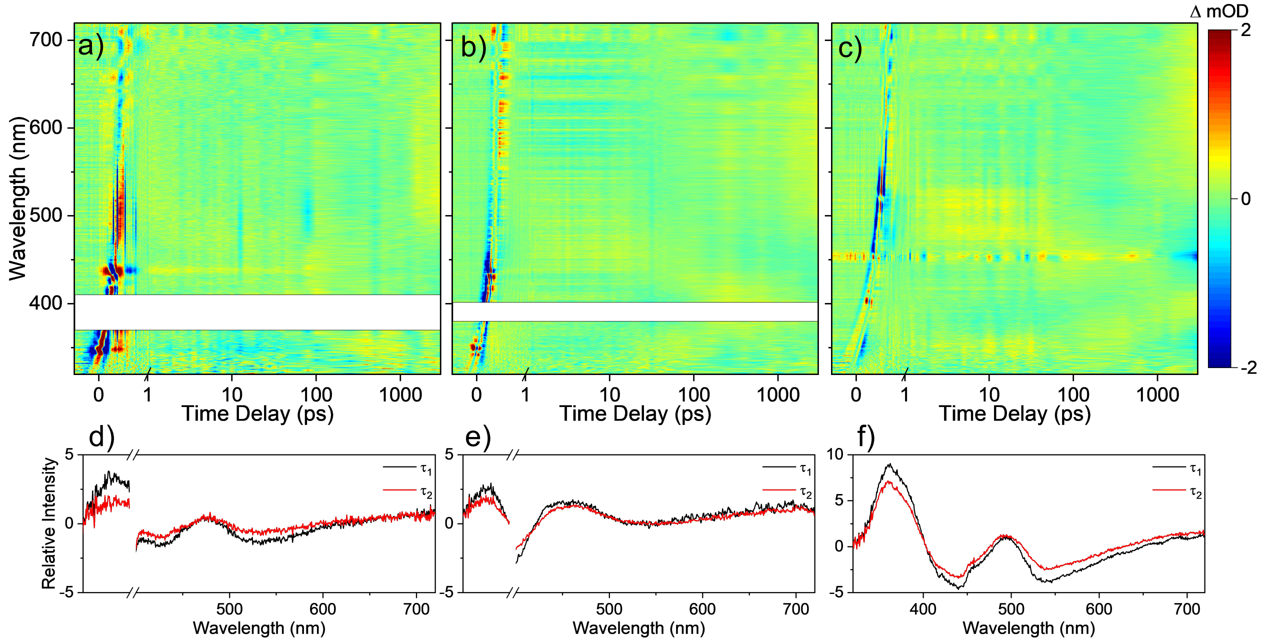

7. Product Characterization

(3a*R*,5a*S*,9a*S*,9b*R*)-3-acetyl-3a,6,6,9a-tetramethyldodecahydro-2*H*-benzo[*e*]indol-2-one (2)

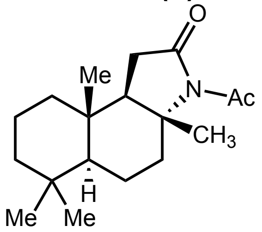

Yield: 78 mg, 17%

Purification Method: Normal phase chromatography (silica; hexanes/Et_2O)

^{1}H NMR ($CDCl_3$, 400 MHz): δ 2.76 (1H, dt, *J*=12.4, 3.3 Hz), 2.38 (3H, s), 2.35-2.25 (1H, m), 1.80-1.69 (2H, m), 1.73-1.56 (1H, m), 1.51 (1H, td, *J*=12.7, 3.8 Hz), 1.48-1.34 (2H, m), 1.32 (3H, s), 1.26-1.09 (2H, m), 1.09-0.90 (2H, m), 0.90 (3H, s), 0.84 (3H, s), 0.81 (3H, s).

^{13}C NMR ($CDCl_3$, 100 MHz): δ 176.77, 171.67, 77.48, 77.16, 76.84, 66.48, 56.93, 56.80, 42.29, 39.57, 38.87, 35.87, 33.18, 33.06, 31.27, 27.03, 21.00, 20.07, 18.71, 18.13, 15.53

HRMS: Calculated for $C_{18}H_{30}NO_2^+$ $(M+H)^+$: 292.2271. Found: 292.2281.

4-methoxybenzoic acid (5)

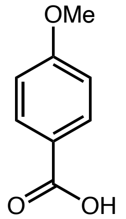

Yield: 38 mg, 17%

Purification Method: Reverse phase chromatography (C18; H_2O/MeCN)

^{1}H NMR ($(CD_3)_2CO$, 500 MHz): δ 7.99 (2H, d, *J*=8.8 Hz), 7.02 (2H, d, *J*=8.8 Hz), 3.88 (3H, s)

^{13}C NMR ($(CD_3)_2CO$, 125 MHz): δ 167.50, 164.42, 132.50, 123.72, 114.53, 55.88

HRMS: Calculated for : $C_8H_9O_3^+$ $(M+H)^+$: 153.0547. Found: 153.0551.

Reference: Spectra are consistent with ref. *8*

_N_-(1-phenylethyl)acetamide (7)

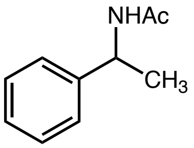

Yield: 64 mg, 26%

Purification Method: Reverse phase chromatography (C18; H_2O/2-propanol)

^{1}H NMR ((CD_3)$_2$CO, 400 MHz): δ 7.98 (1H, br. s), 7.34-7.25 (4H, m), 7.20 (1H, t, *J*=7.5 Hz), 5.05 (1H, quint., *J*=7.3 Hz), 1.94 (3H, s), 1.40 (3H, d, *J*=7.0 Hz)

^{13}C NMR ((CD_3)$_2$CO, 100 MHz): δ 170.81, 145.01, 129.19, 127.70, 126.96, 49.66, 22.54, 22.47

Reference: Spectra are consistent with ref. *9*

Pinacolone (9)

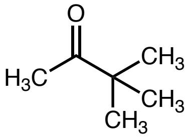

Yield: 54 mg, 36%

Purification Method: Normal phase chromatography (silica; hexanes/DCM)

^{1}H NMR ($CDCl_3$, 400 MHz): δ

^{13}C NMR ($CDCl_3$, 100 MHz): δ

Reference: Spectra are consistent with authentic material.

N-($tert$-butyl)acetamide (11)

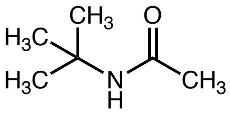

Yield: 102 mg, 59%

Purification Method: Reverse phase chromatography (C18; H_2O/2-propanol)

^{1}H NMR ($CDCl_3$, 400 MHz): δ 7.07 (1H, br. s), 1.86 (3H, s), 1.19 (9H, s)

^{13}C NMR ($CDCl_3$, 100 MHz): δ 171.27, 51.56, 28.33, 23.17

Reference: Spectra are consistent with ref. *10*

Acetophenone (13)

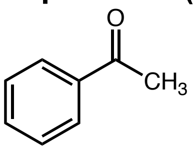

Yield: 34 mg, 19%

Purification Method: Normal phase chromatography (silica; hexanes/DCM)

^{1}H NMR ($CDCl_3$, 400 MHz): δ 7.94 (2H, dt, J=8.1, 1.2 Hz), 7.54 (1H, dd, J=7.2, 1.5 Hz), 7.49-7.39 (2H, m), 2.59 (3H, s)

^{13}C NMR ($CDCl_3$, 100 MHz): δ 198.16, 137.12, 133.12, 128.58, 128.31, 26.62

Reference: Spectra are consistent with authentic material.

Phthalide (15)

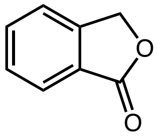

Yield: 28 mg, 14%

Purification Method: Normal phase chromatography (silica; hexanes/DCM)

^{1}H NMR ($CDCl_3$, 400 MHz): δ 7.93 (1H, dt, *J*=7.7, 1.1 Hz), 7.68 (1H, dd, *J*=7.5, 1.1 Hz), 7.58-7.48 (2H, m), 5.33 (2H, s)

^{13}C NMR ($CDCl_3$, 100 MHz): δ 171.24, 146.66, 134.15, 129.18, 125.93,

Reference: Spectra are consistent with authentic material.

***N*-cyclopentylacetamide (16a)**

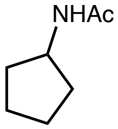

Yield: 69 mg, 36%

Purification Method: Reverse phase chromatography (C18; H_2O/Acetone)

^{1}H NMR ($CDCl_3$, 500 MHz): δ 5.41-5.30 (1H, br. s), 4.20 (1H, sept, *J*= 7.0 Hz), 1.96 (3H, s), 1.74-1.59 (6H, m), 1.41-1.31 (2H, m)

^{13}C NMR ($CDCl_3$, 125 MHz): δ 169.91, 51.40, 33.29, 23.81

HRMS: Calculated for $C_7H_{14}NO^+$ $(M+H)^+$: 128.1070. Found: 128.1074.

Reference: Spectra are consistent with ref. *11*

_N_-cyclohexylacetamide (16b)

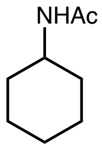

Yield: 38 mg, 18%

Purification Method: Reverse phase chromatography (C18; H_2O/Acetone)

^{1}H NMR ($CDCl_3$, 500 MHz): δ 5.34 (1H, br. s), 3.67 (1H, tdt, *J*=10.8, 8.1, 4.0 Hz), 1.87 (3H, s), 1.86-1.80 (2H, m), 1.62 (2H, dddd, *J*=15.2, 7.9, 4.0, 1.3 Hz), 1.57-1.48 (1H, m), 1.33-1.21 (2H, m), 1.12-0.96 (3H, m)

^{13}C NMR ($CDCl_3$, 125 MHz): δ 169.07, 48.23, 33.23, 25.53, 24.88, 23.61

HRMS: Calculated for $C_8H_{16}NO^+$ $(M+H)^+$: 142.1227. Found: 142.1231.

Reference: Spectra are consistent with ref. *12*

_N_-cycloheptylacetamide (16c)

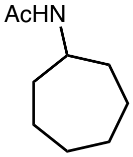

Yield: 60 mg, 26%

Purification Method: Reverse phase chromatography (C18; H_2O/Acetone)

^{1}H NMR ($CDCl_3$, 500 MHz): δ 5.50 (1H, br. s), 3.92 (1H, tt, *J*=8.9, 4.4 Hz), 1.93 (3H, s), 1.92-1.87 (1H, m), 1.64-1.54 (4H, m), 1.53-1.34 (6H, m)

^{13}C NMR ($CDCl_3$, 125 MHz): δ 169.84, 50.60, 35.31, 28.18, 24.16, 23.76

HRMS: Calculated for$C_9H_{18}NO^+$ $(M+H)^+$: 156.1383. Found: 156.1391.

Reference: Spectra are consistent with ref. *11*

N-cyclooctylacetamide (16d)

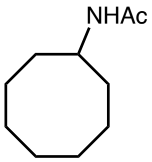

Yield: 75 mg, 29%

Purification Method: Reverse phase chromatography (C18; H_2O/MeCN)

^{1}H NMR ($CDCl_3$, 500 MHz): δ 5.44 (1H, br. s), 3.97 (1H, tt, *J*=8.4, 4.3 Hz), 1.94 (3H, s), 1.81 (2H, ddd, *J*=13.5, 6.5, 3.2 Hz), 1.67-1.45 (12H, m)

^{13}C NMR ($CDCl_3$, 125 MHz): δ 168.85, 49.52, 32.30, 27.37, 25.49, 23.78, 23.74

HRMS: Calculated for $C_{10}H_{20}NO^+$ $(M+H)^+$: 170.1539. Found: 170.1544.

Reference: Spectra are consistent with ref. *11*

N-cyclododecylacetamide (16e)

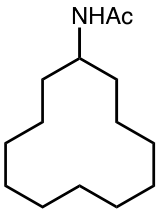

Yield: 106 mg, 37%

Purification Method: Reverse phase chromatography (C18; H_2O/2-propanol)

^{1}H NMR ($CDCl_3$, 500 MHz): δ 5.42 (1H, br. s), 4.03-4.00 (1H, m), 1.93 (3H, s), 1.59-1.25 (22H, m)

^{13}C NMR ($CDCl_3$, 125 MHz): δ 169.47, 46.31, 30.21, 24.11, 23.87, 23.63, 23.52, 23.41, 21.44

HRMS: Calculated for $C_{14}H_{28}NO^+$ $(M+H)^+$: 226.2165. Found: 226.2175.

Reference: Spectra are consistent with ref. *11*

N-((2*S*)-norbornyl)acetamide (17)

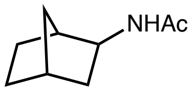

Yield: 74 mg, 32%

Purification Method: Reverse phase chromatography (C18; H_2O/Acetone)

^{1}H NMR ($CDCl_3$, 500 MHz): δ 5.20 (1H, s), 3.68-3.60 (1H, m), 2.19 (1H, d, *J*=4.5 Hz), 2.11 (1H, d, *J*=14.1 Hz), 1.86 (3H, s), 1.73 (1H, ddd, *J*=13.2, 8.0, 2.4 Hz), 1.46-1.33 (2H, m), 1.27-1.16 (2H, m), 1.16-1.10 (1H, m), 1.05 (2H, tdd, *J*=13.3, 7.3, 3.7 Hz)

^{13}C NMR ($CDCl_3$, 125 MHz): δ 169.26, 52.95, 35.83, 35.72, 28.25, 26.61, 23.68

HRMS: Calculated for $C_9H_{16}NO^+$ $(M+H)^+$: 154.1226. Found: 154.1240.

Reference: Spectra are consistent with ref. *13*

N-(1-adamantyl)acetamide (18)

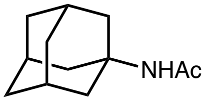

Yield: 118 mg, 41%

Purification Method: Reverse phase chromatography (C18; H_2O/Acetone) followed by normal phase chromatography (silica; hexanes/$CHCl_3$)

^{1}H NMR ($CDCl_3$, 500 MHz): δ 5.10 (1H, br. s), 2.09-2.04 (3H, br. s), 1.98 (6H, d, *J*= 2.9 Hz), 1.90 (3H, s), 1.69-1.66 (6H, br. s)

^{13}C NMR ($CDCl_3$, 125 MHz): δ 169.4, 52.2, 41.8, 36.6, 29.6

HRMS: Calculated for $C_{12}H_{20}NO^+$ $(M+H)^+$: 194.1539. Found: 194.1541.

Reference: Spectra are consistent with ref. *12*

Methylcyclohexane Acetamide (19)

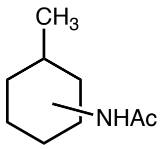

Yield: 55 mg, 24%

Purification Method: Reverse phase chromatography (C18; H_2O/acetone then C18; H_2O/2-propanol)

^{1}H NMR ($CDCl_3$, 500 MHz), *characteristic peaks only:* δ 5.59 (3.11 H, br. s), 5.52 (1.46 H, br. s), 5.44 (0.70 H, br. s), 5.31 (1.35 H, br. s), 5.24 (1.05 H, br. s), 4.21-4.05 (11.49 H, m), 3.98-4.05 (1.54 H, m), 3.95 (1.11 H, tt, *J*=8.7, 4.6 Hz), 3.75 (2.34 H, dtt, *J*=11.9, 7.9, 3.9 Hz), 3.63-3.71 (1.00 H, m), 3.48 (1.94 H, qd, *J*=10.4, 4.0 Hz)

HRMS: Calculated for $C_9H_{18}NO^+$ $(M+H)^+$: 156.1383. Found: 156.1390.

Reference: Spectra are consistent with ref. *14*

trans-decalin Acetamide (20)

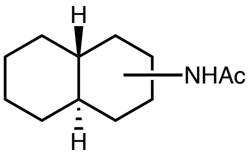

Yield: 27 mg, 9%

Purification Method: Reverse phase chromatography (C18; H_2O/acetone then C18; H_2O/2-propanol)

^{1}H NMR ($CDCl_3$, 500 MHz), *characteristic peaks only:* δ 5.68-5.60 (1.03 H, br. s), 5.55-5.49 (0.63 H, br. s), 5.27-5.18 (1.03 H, br. s), 5.13-5.03 (1.49 H, br. s), 4.22-4.15 (1.15 H, m), 4.12-4.06 (0.65 H, m), 3.85-3.73 (1.17 H, m), 3.56 (1.80 H, ddd, *J*= 21.1, 10.9, 4.0 Hz)

HRMS: Calculated for $C_{12}H_{22}NO^+$ $(M+H)^+$: 196.1696. Found: 196.1700.

Reference: Spectra are consistent with ref. *11*

Hexyl Acetamide (21)

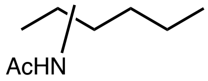

Yield: 70 mg, 32%. Isolated as a 66:34 ratio of C2:C3 amidated product. Assignment was based on the characteristic doublet at 1.10 for the C2 product and ratios determined by the α-NH protons.

Purification Method: Reverse phase chromatography (C18; H_2O/MeOH)

^{1}H NMR ($CDCl_3$, 400 MHz): δ 5.36 (1.42 H, br. s), 5.26 (0.70 H, br. s), 3.94 (1.95 H, dp, *J*= 8.7, 6.6 Hz), 3.89-3.79 (1.00 H, m), 1.96 (3.03 H, s), 1.94 (5.81 H, s), 1.56-1.46 (1.29 H, m), 1.40 (6.33 H, dddd, *J*=12.3, 7.4, 6.1, 3.2 Hz), 1.34-1.23 (11.66 H, m), 1.10 (5.92, d, *J*= 6.6 Hz), 0.88 (12.36 H, td, *J*= 7.2, 5.8 Hz)

^{13}C NMR ($CDCl_3$, 100 MHz): δ 169.77, 169.68, 169.39, 169.30, 77.42, 77.16, 76.90, 50.51, 50.40, 45.40, 45.28, 37.04, 37.01, 36.78, 36.75, 28.29, 28.15, 28.04, 28.02, 23.68, 23.65, 23.63, 23.59, 22.70, 21.10, 21.07, 19.22, 14.16, 14.12, 10.25

HRMS: Calculated for $C_8H_{18}NO^+$ $(M+H)^+$: 144.1383. Found: 144.1386.

Reference: Spectra are consistent with ref. *14*

***N*-(1-phenylhexyl)acetamide (22)**

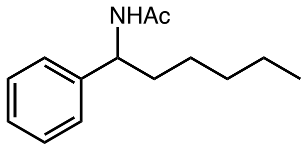

Yield: 69 mg, 21%

Purification Method: Reverse phase chromatography (C18; H_2O/Acetone)

^{1}H NMR ($CDCl_3$, 400 MHz): δ 7.31-7.14 (5H, m), 5.90 (1H, d, *J*=8.4 Hz), 4.88 (1H, q, *J*=7.7 Hz), 1.90 (3H, s), 1.78-1.65 (2H, m), 1.30-1.12 (6H, m), 0.85-0.76 (m, 3H)

^{13}C NMR ($CDCl_3$, 125 MHz): δ 169.34, 142.63, 128.70, 127.35, 126.69, 53.61, 36.28, 31.66, 26.01, 23.52, 22.58, 18.35, 14.09

HRMS: Calculated for : $C_{14}H_{22}NO^+$ $(M+H)^+$: 220.1696. Found: 220.1694.

Note: Most peaks in the ^{13}C spectrum appeared as doublets. This phenomenon was consistent with regards to various solvents (CD_3OD and CD_3CN).

Reference: Spectra are consistent with ref. *15*

N-((2S)-norbornyl)benzamide (23)

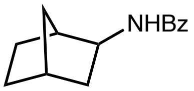

Yield: 145 mg, 67%

Purification Method: Silica gel (*ca.* 30 g) was added to the crude mixture, which was briefly stirred then filtered. The silica was washed with CH_2Cl_2 (150 mL) to remove benzonitrile. The reaction products were desorbed from silica by washing with 1:1 CH_2Cl_2 : acetone (3 x 50 mL), concentrated under reduced pressure, and purified by reverse phase chromatography (C18; H_2O/Acetone).

^{1}H NMR ($CDCl_3$, 400 MHz): δ 7.77-7.70 (2H, m), 7.50-7.46 (1H, m), 7.44-7.39 (2H, m), 5.94 (1H, br. s), 3.95-3.90 (1H, m), 2.33 (2H, dt, *J*=3.3, 1.6 Hz), 1.90 (1H, ddd, *J*=13.3, 8.0, 2.5 Hz), 1.61-1.44 (3H, m), 1.38 (1H, dp, *J*=10.2, 2.0 Hz), 1.36-1.23 (m, 4H), 1.18 (1H, dddd, *J*=11.7, 9.6, 3.8, 2.2 Hz)

^{13}C NMR ($CDCl_3$, 100 MHz): δ 166.91, 135.13, 131.40, 128.66, 126.93, 53.45, 42.58, 40.75, 35.91, 35.85, 28.28, 26.65

HRMS: Calculated for $C_9H_{16}NO^+$ $(M+H)^+$: 216.1383. Found: 216.1385.

Reference: Spectra are consistent with ref. *16*

N-((2S)-norbornyl)isobutyramide (24)

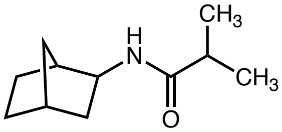

Yield: 103 mg, 38%

Purification Method: Reverse phase chromatography (C18; H_2O/EtOH)

^{1}H NMR (500 MHz, CD_3CN): δ 6.19 (1H, br. s), 3.55 (1H, q, *J*=6.0 Hz), 2.29 (1H, quint, *J*=6.7 Hz), 2.22 (1H, s), 2.06 (1H, d, *J*=3.9 Hz), 1.66 (1H, ddd, *J*=12.9, 8.1, 2.5 Hz), 1.46 (1H, q, *J*=6.8, 2.9 Hz), 1.39 (1H, dt, *J*=9.7, 1.9 Hz), 1.31-1.09 (3H, m), 1.03 (9H, dt, *J*=6.8, 2.9 Hz)

^{13}C NMR (125 MHz, CD_3CN): δ 175.26, 51.80, 41.87, 35.02, 34.86, 33.62, 28.02, 26.15, 19.67, 19.52

HRMS: Calculated for $C_{11}H_{20}NO^+$ $(M+H)^+$: 182.1539. Found: 182.1546.

Reference: Spectra are consistent with ref. *16*

***N*-((2*S*)-norbornyl)propionamide (25)**

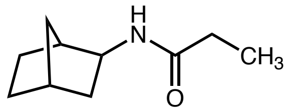

Yield: 88 mg, 35%

Purification Method: Reverse phase chromatography (C18; H_2O/2-propanol)

^{1}H NMR (CD_3OD, 400 MHz): δ 3.57 (1H, dp, *J*=4.8, 2.3 Hz), 2.25 (1H, d, *J*=4.2 Hz), 2.23-2.08 (3H, m), 1.69 (1H, ddd, *J*=13.0, 8.2, 2.4 Hz), 1.57-1.40 (3H, m), 1.38-1.13 (4H, m), 1.10 (3H, t, *J*=7.6 Hz)

^{13}C NMR ($CDCl_3$, 125 MHz): δ 172.96, 52.77, 42.53, 40.75, 35.84, 35.71, 30.00, 28.24, 26.61, 10.08

HRMS: Calculated for $C_{10}H_{18}NO^+$ $(M+H)^+$: 168.1383. Found: 168.1387.

Reference: Spectra are consistent with ref. *13*

8. NMR Spectra

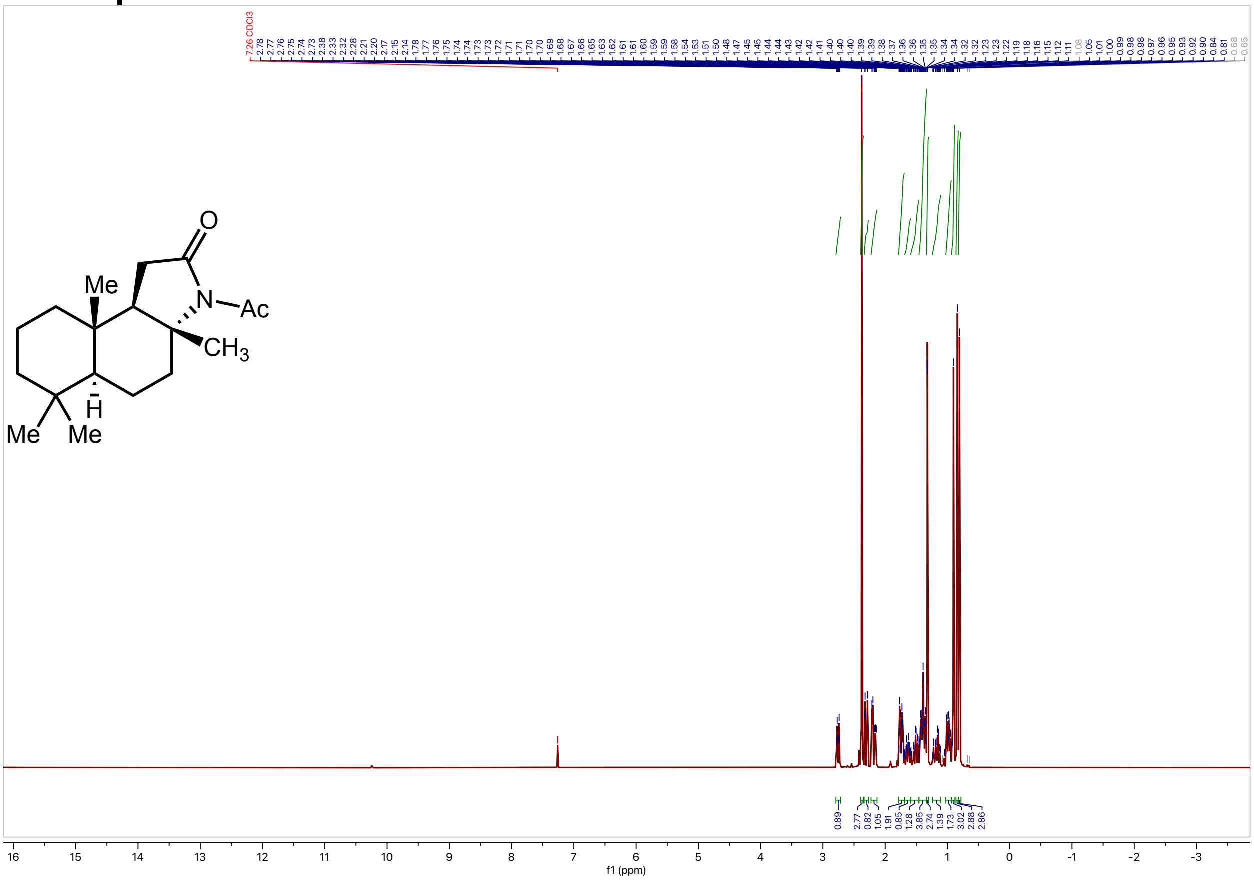

2; ^{1}H NMR, $CDCl_3$, 400 MHz

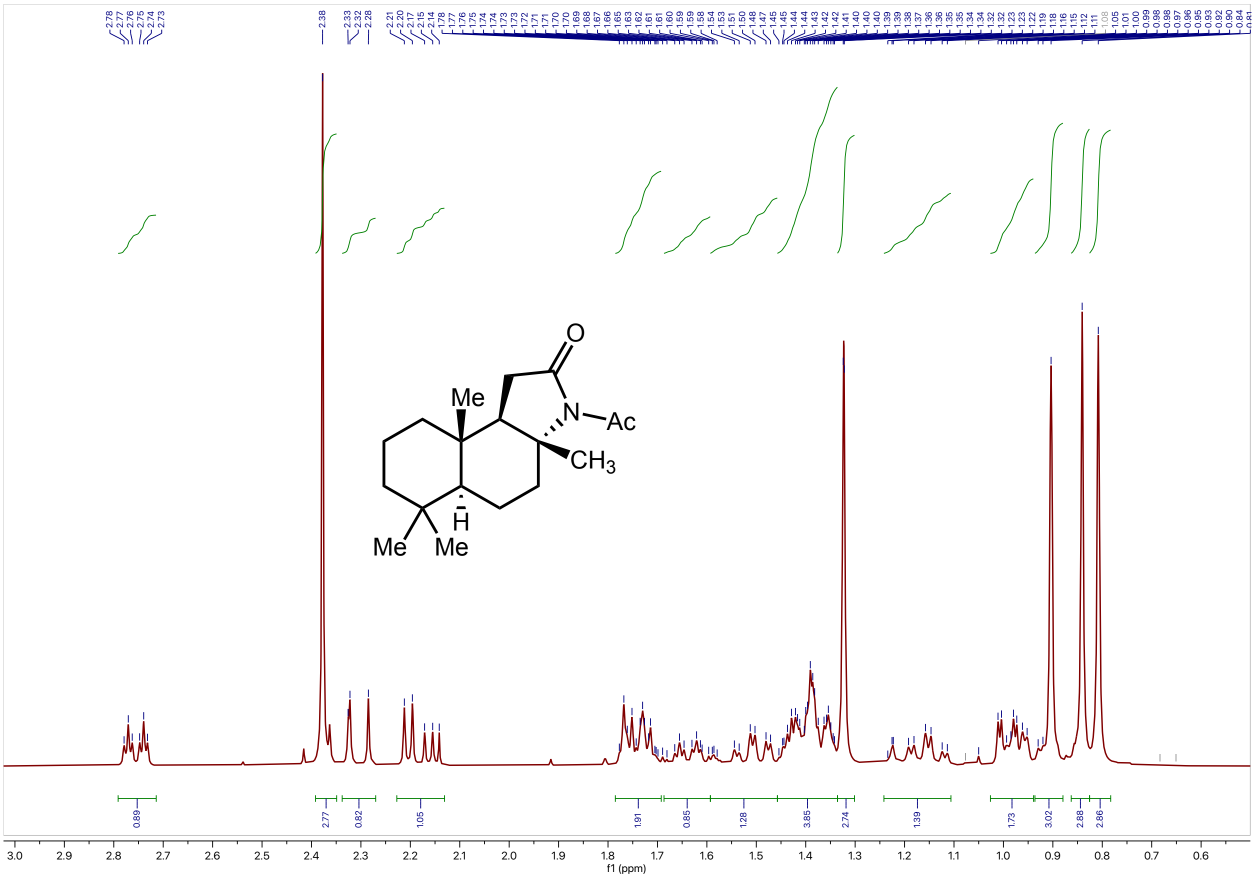

2 zoom; ^{1}H NMR, $CDCl_3$, 400 MHz

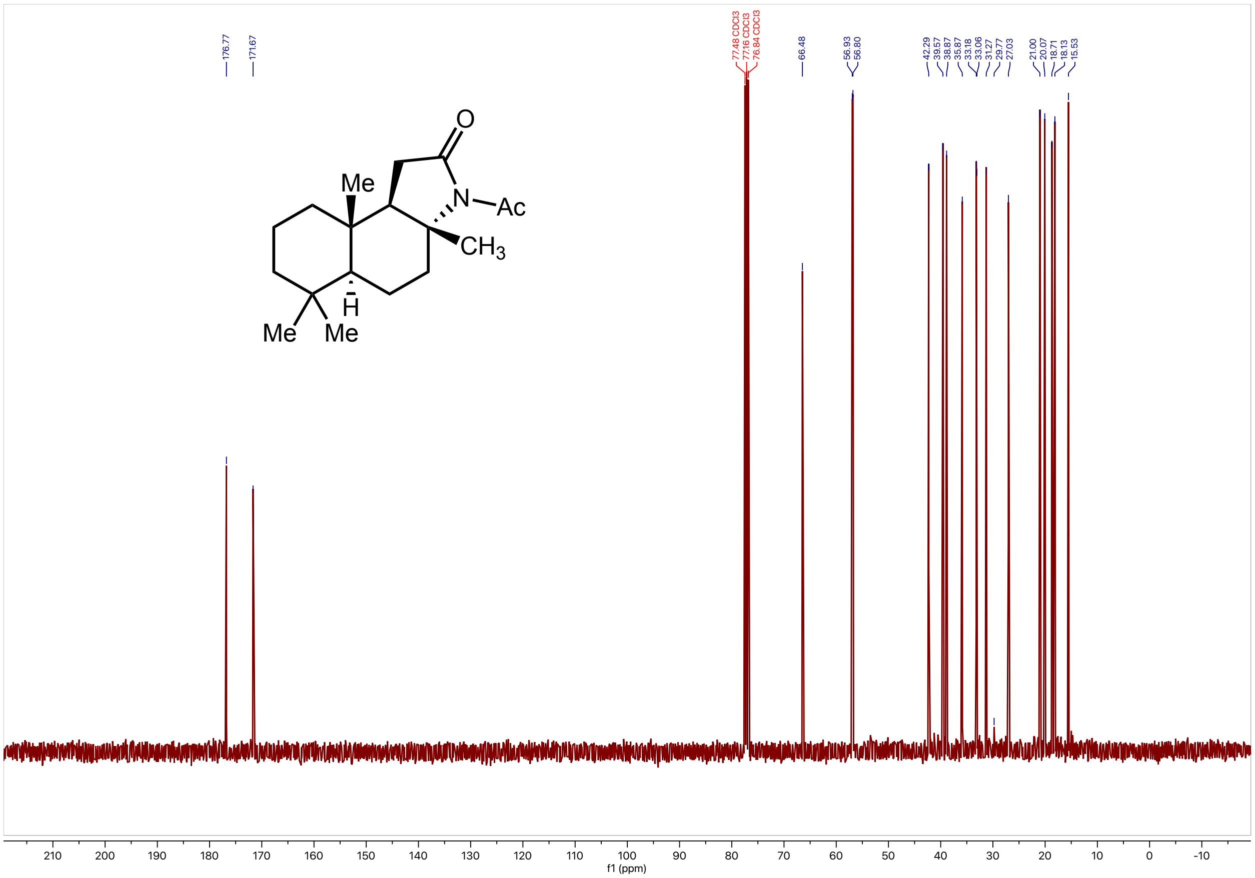

2; ^{13}C NMR, $CDCl_3$, 100 MHz

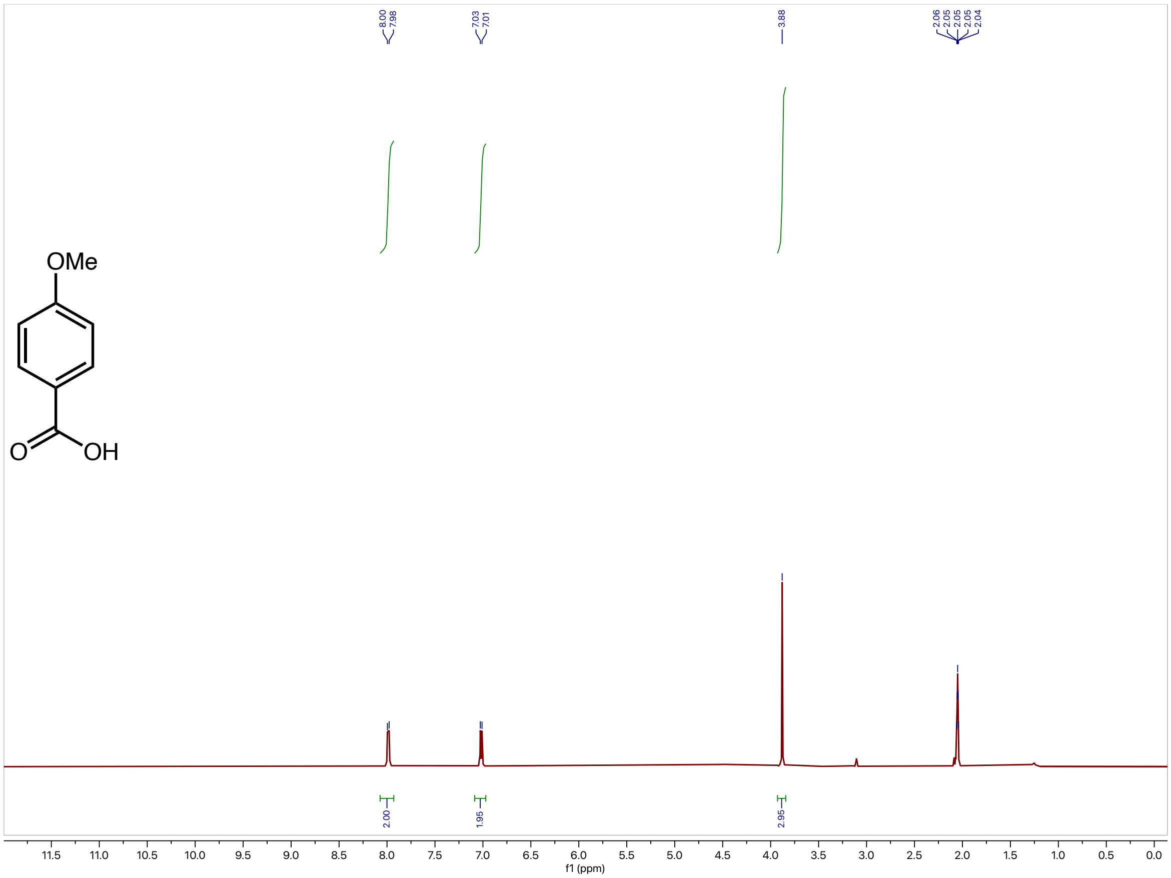

5; ^{1}H NMR, $(CD_3)_2CO$, 500 MHz

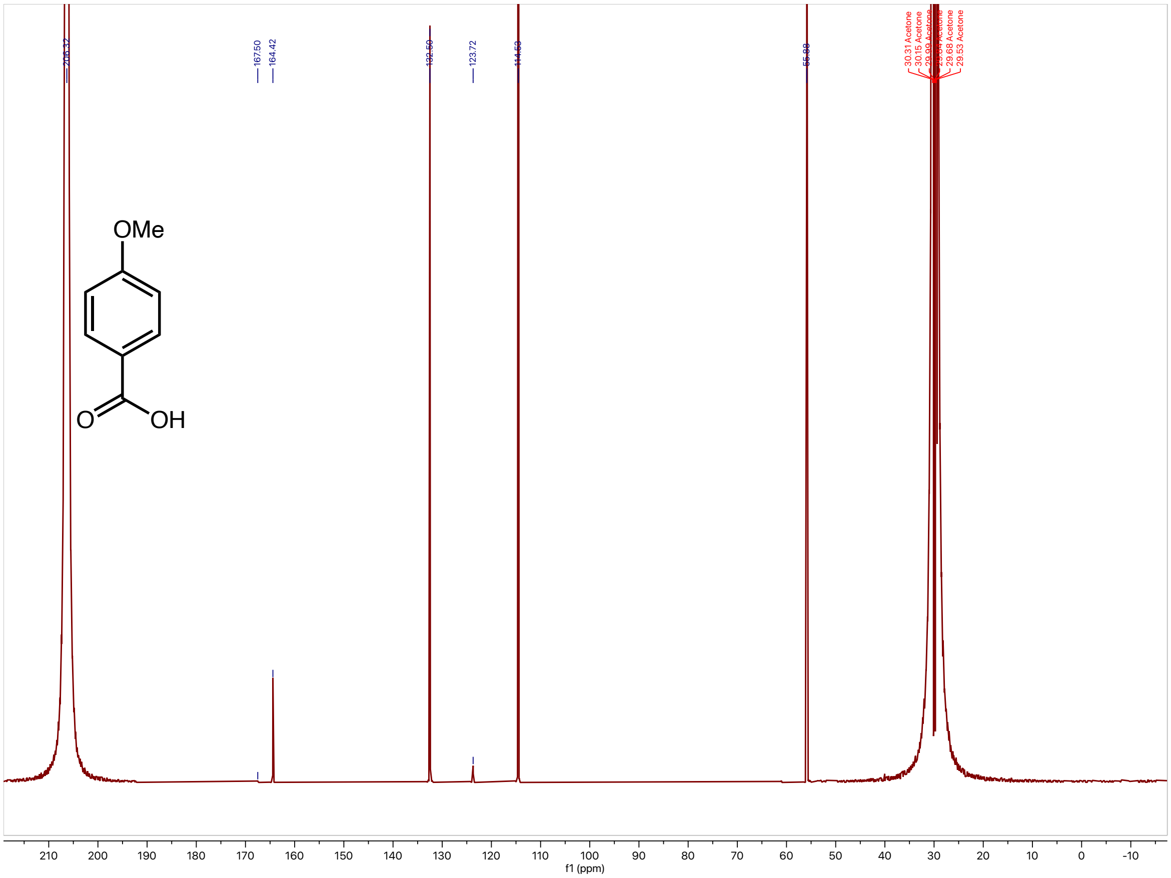

5; ^{13}C NMR, $(CD_3)_2CO$, 125 MHz

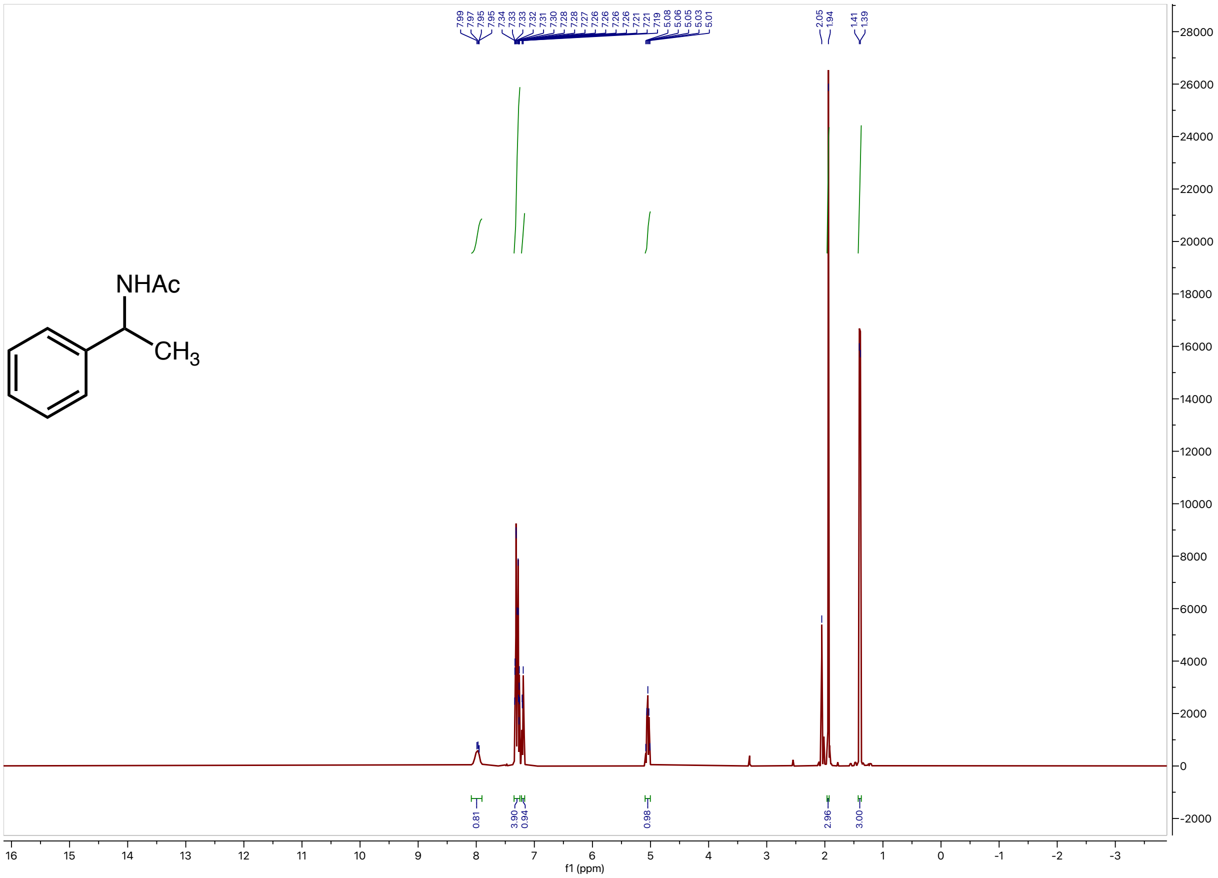

7; ^{1}H NMR, $(CD_3)_2CO$, 400 MHz

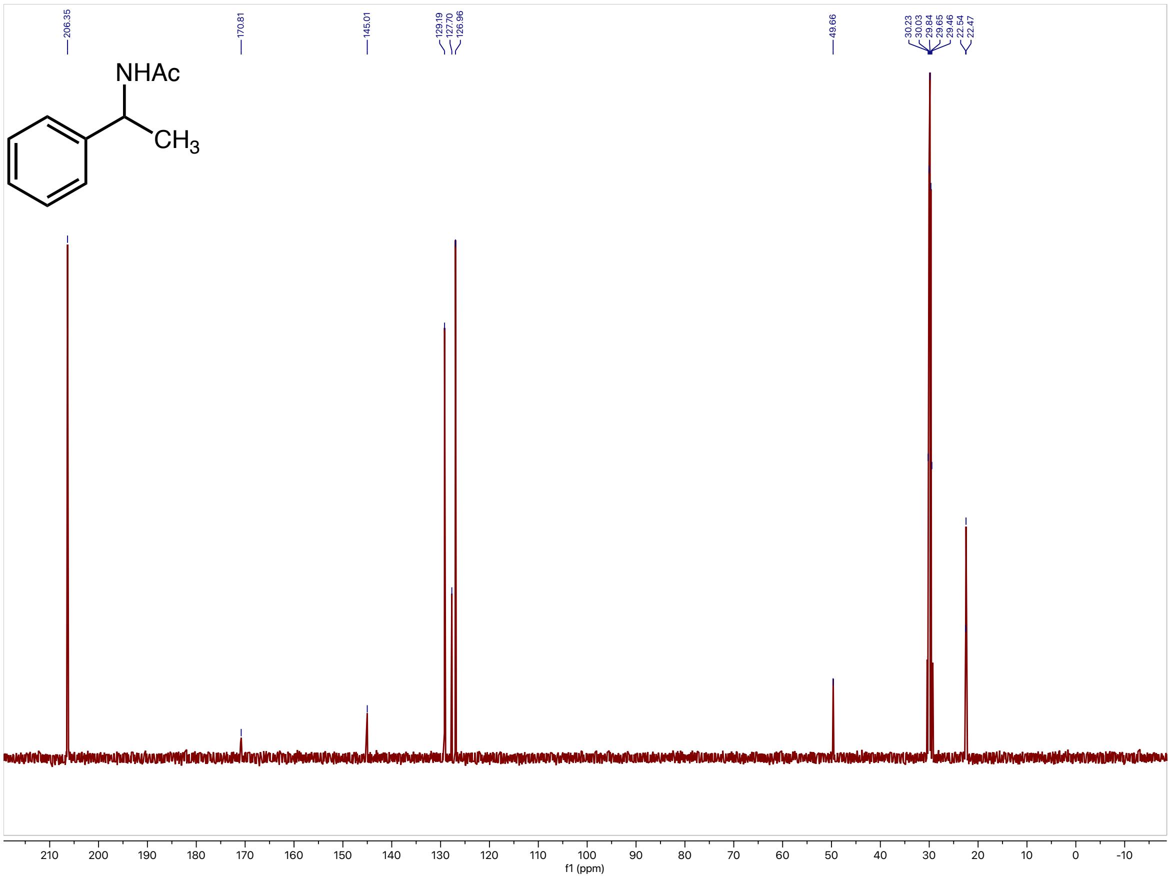

7; ^{13}C NMR, $(CD_3)_2CO$, 100 MHz

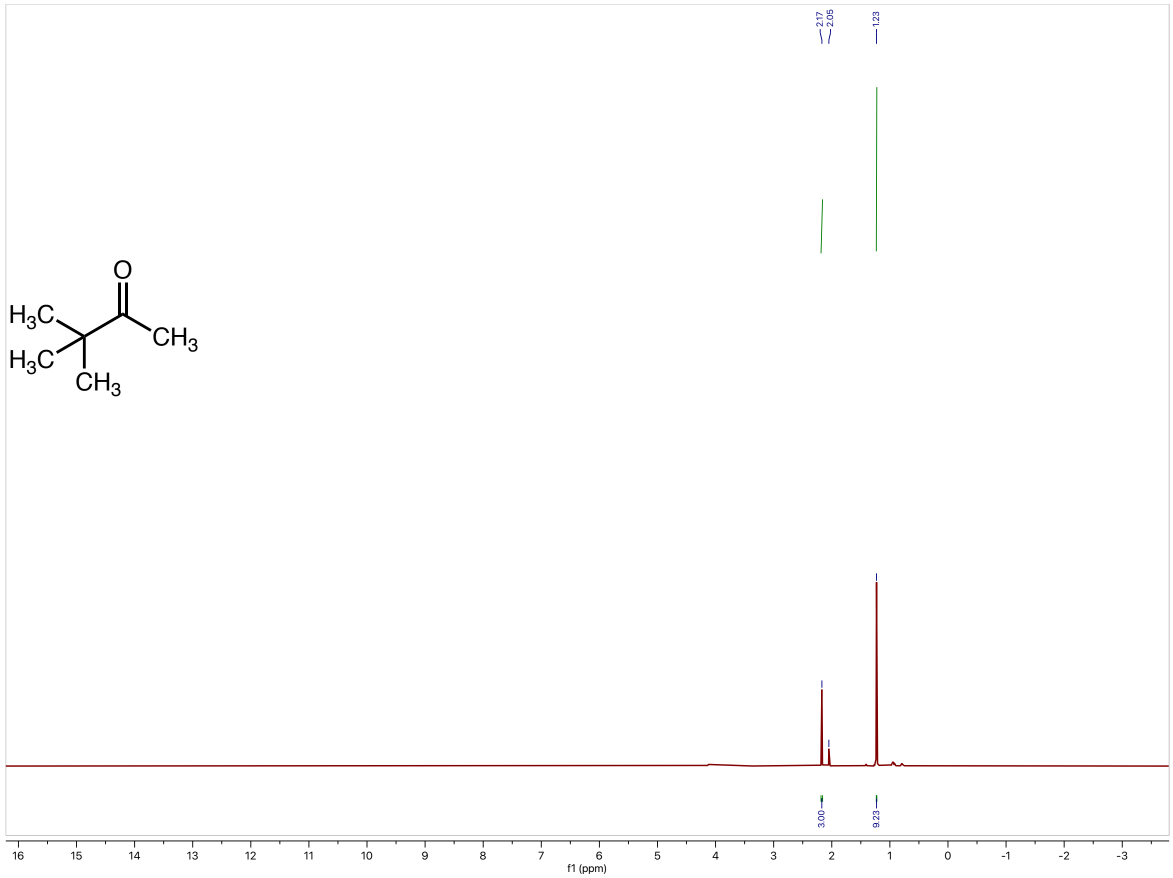

9; ^{1}H NMR, $(CD_3)_2CO$, 400 MHz

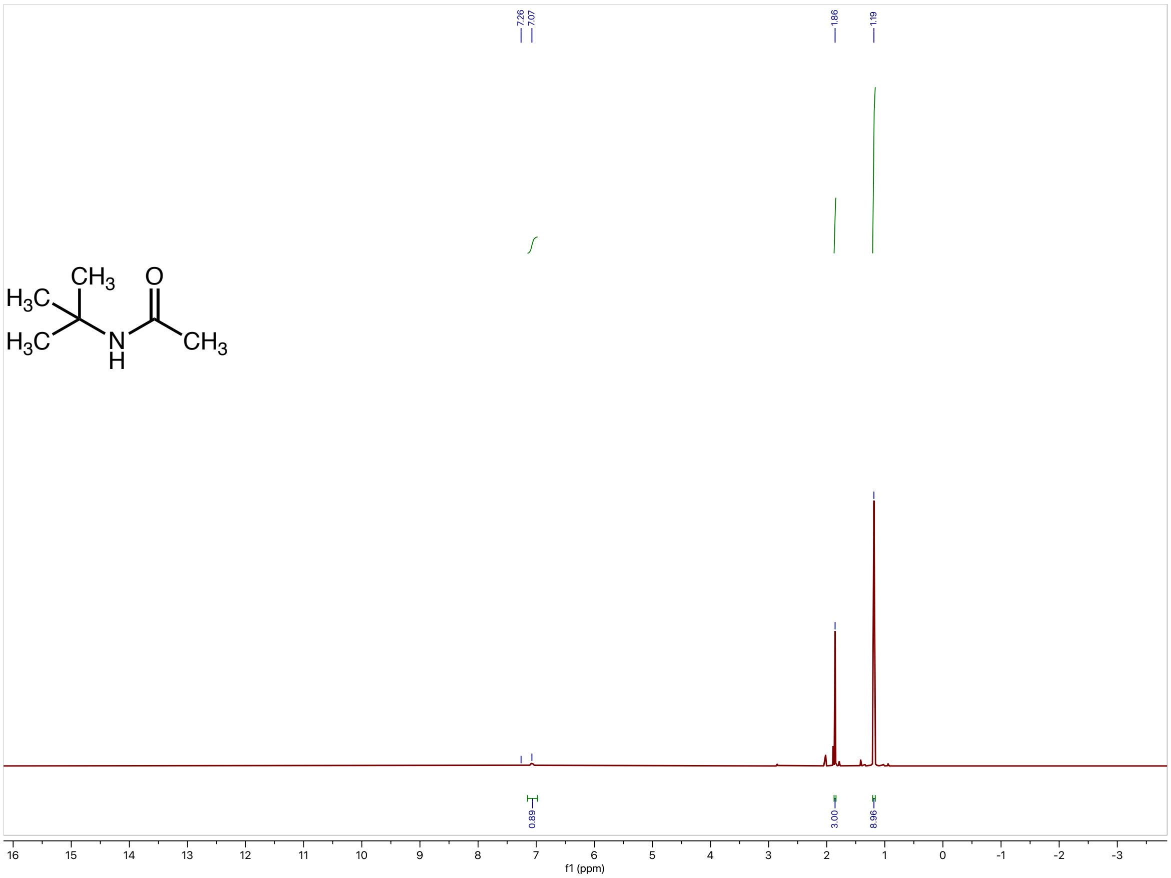

11; ^{1}H NMR, $CDCl_3$, 400 MHz

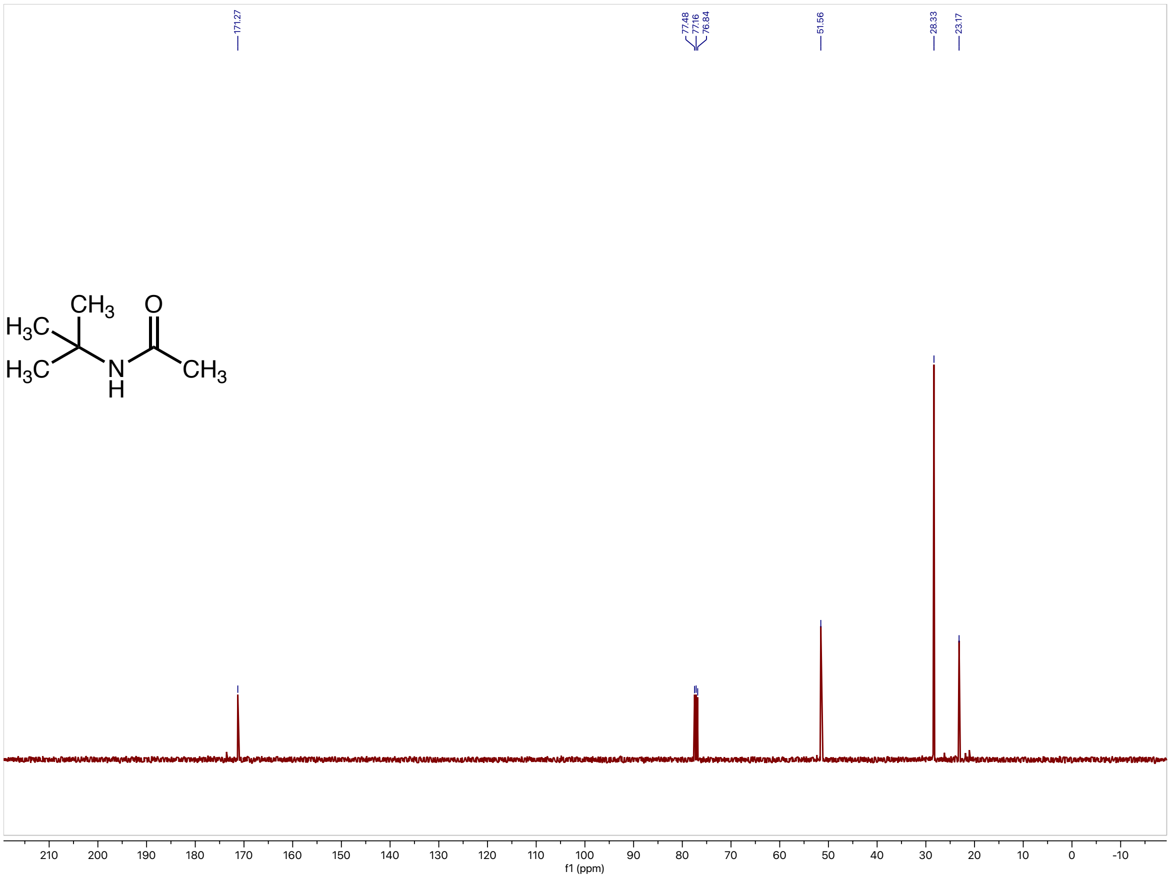

11; ^{13}C NMR, $CDCl_3$, 100 MHz

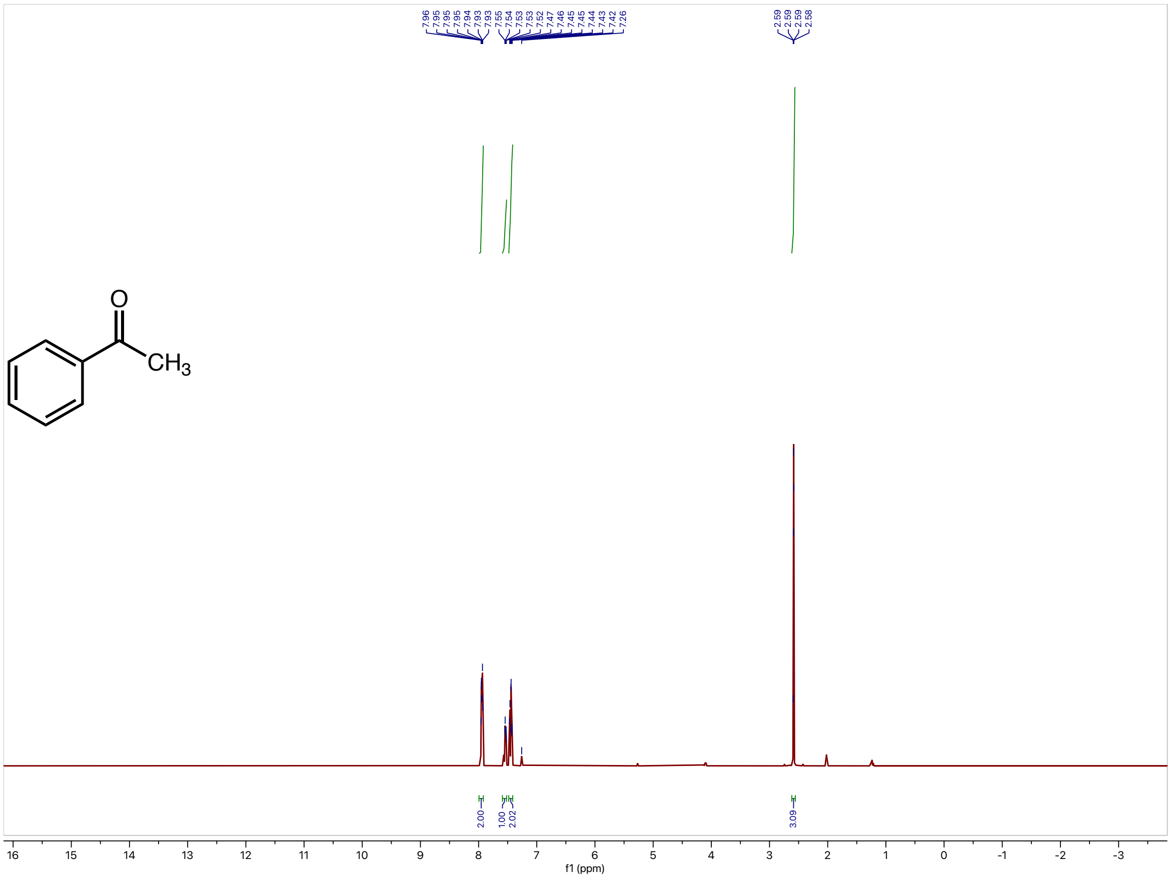

13; ^{1}H NMR, $CDCl_3$, 400 MHz

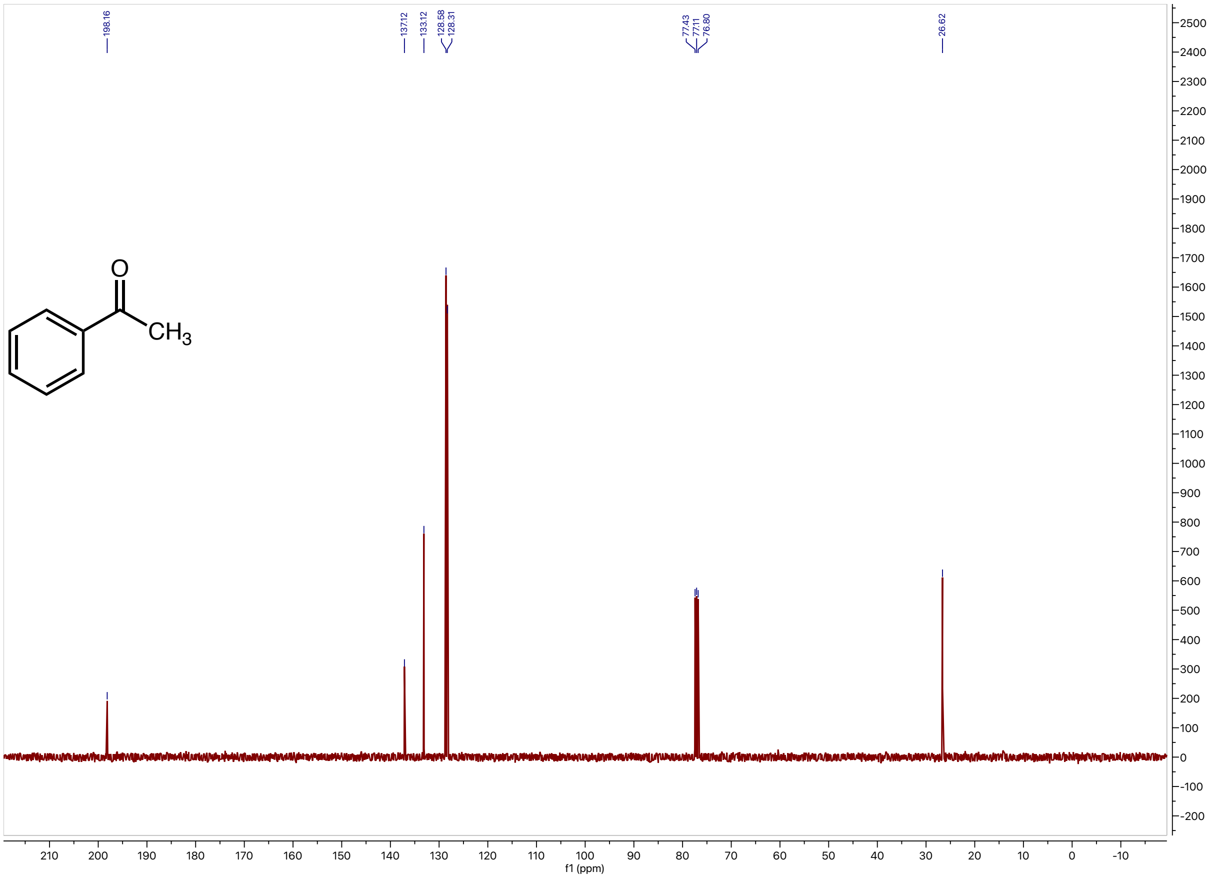

13; ^{13}C NMR, $CDCl_3$, 100 MHz

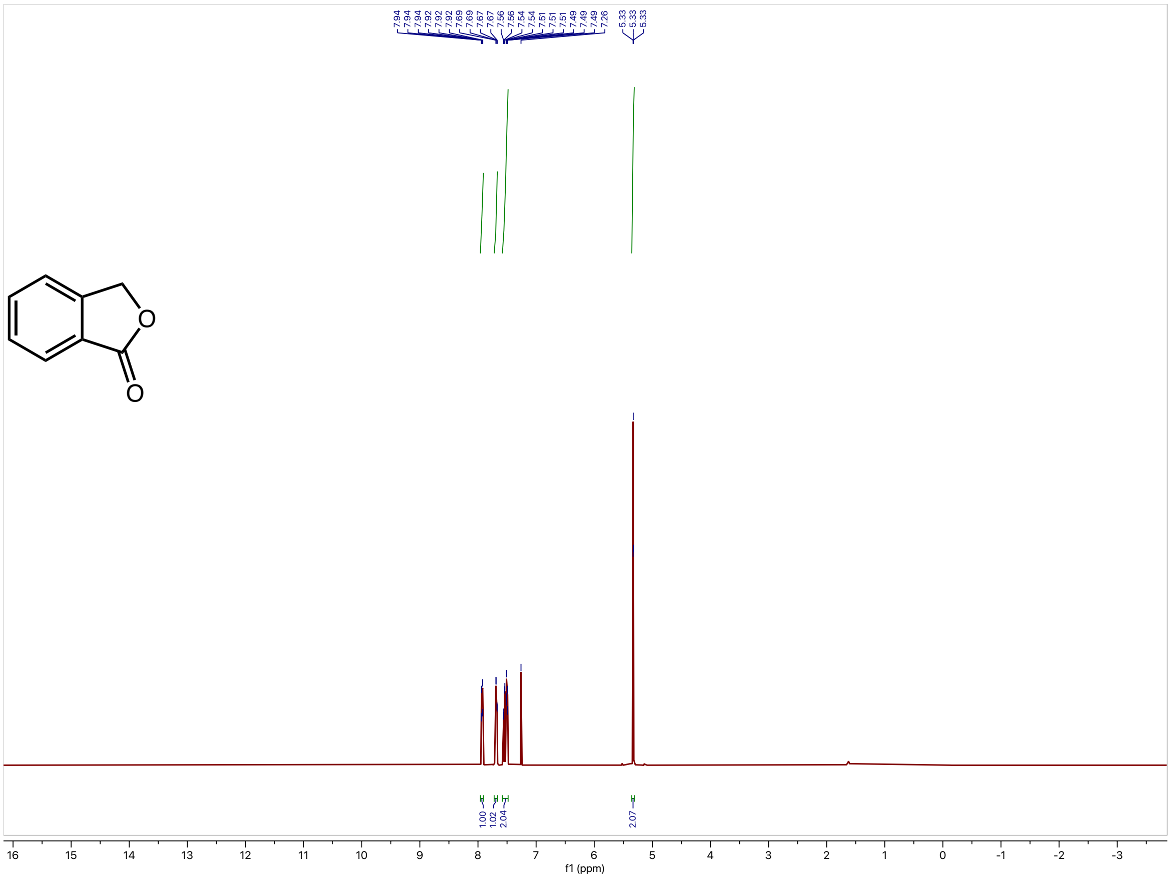

15; ^{1}H NMR, $CDCl_3$, 400 MHz

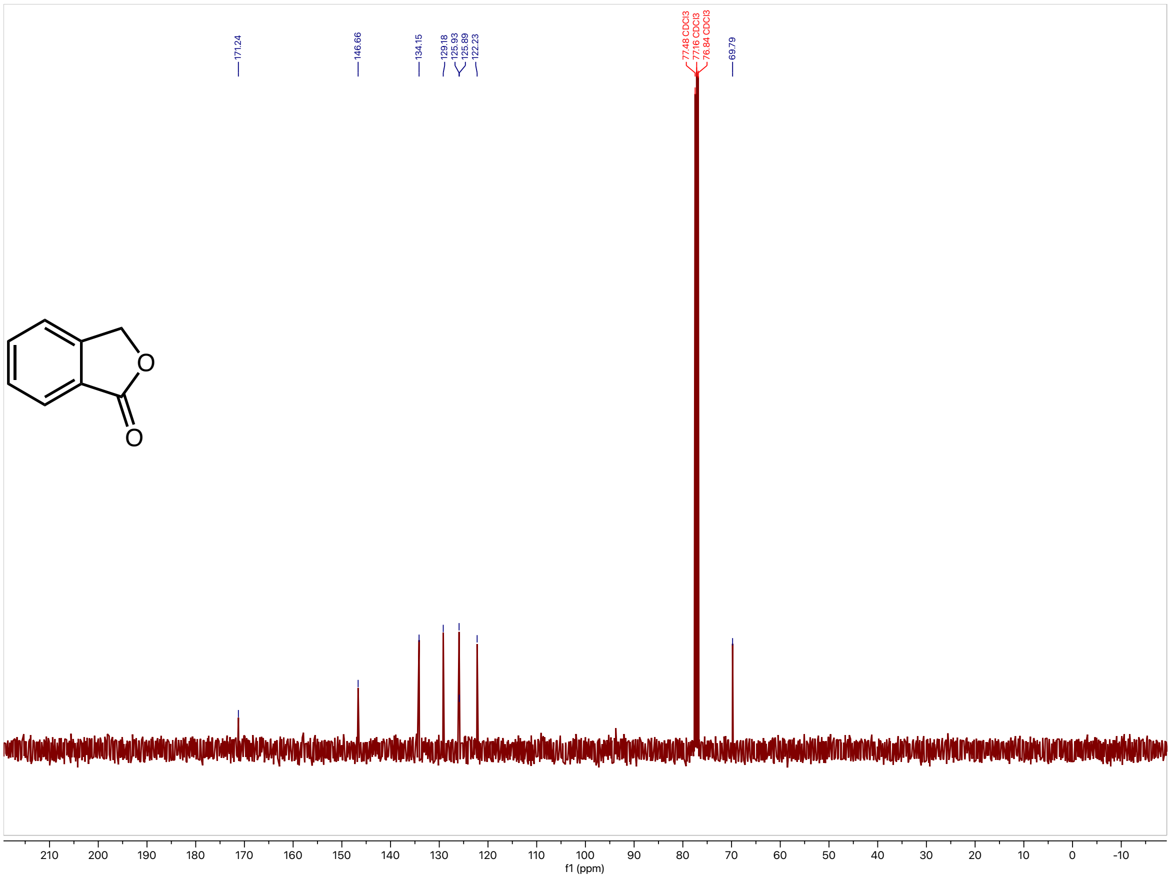

15; ^{13}C NMR, $CDCl_3$, 100 MHz

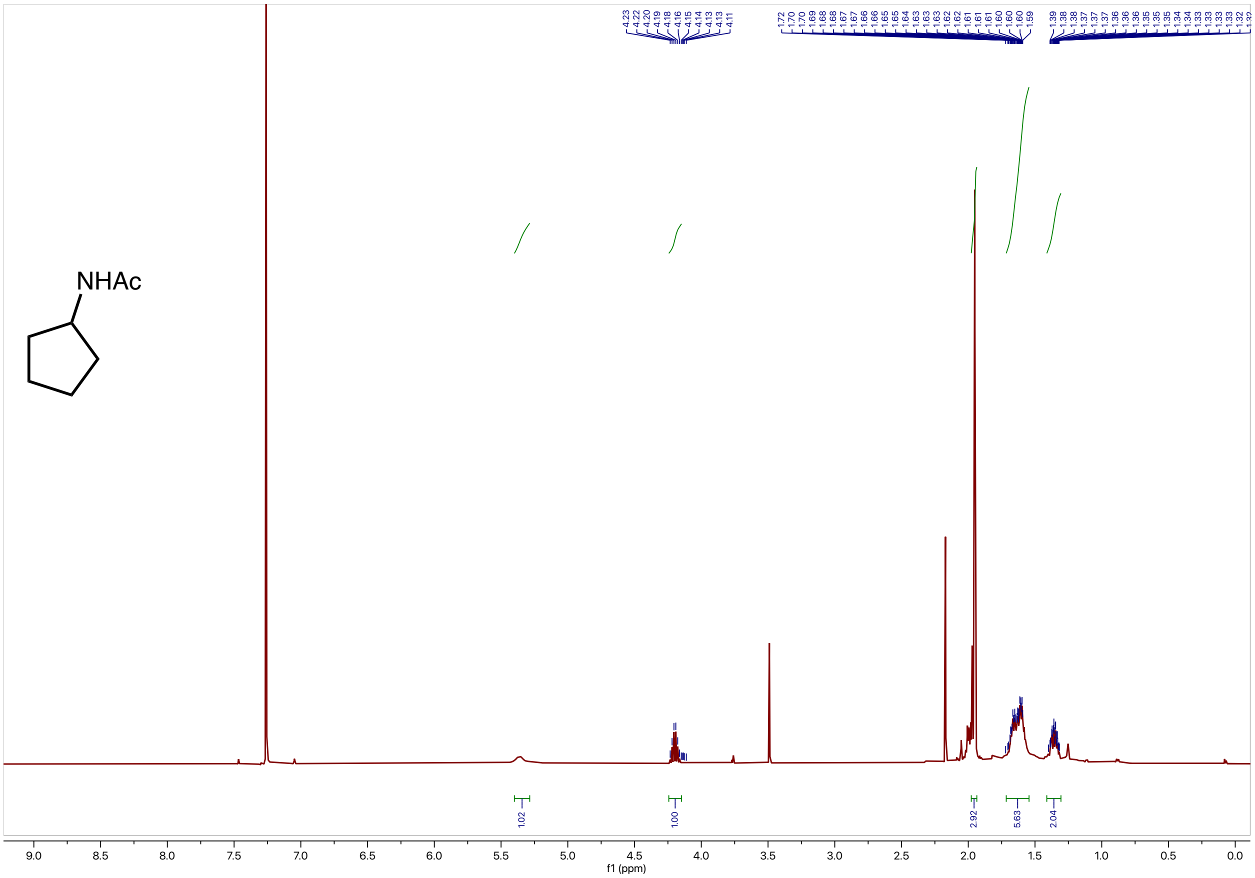

16a; ^{1}H NMR, $CDCl_3$, 500 MHz

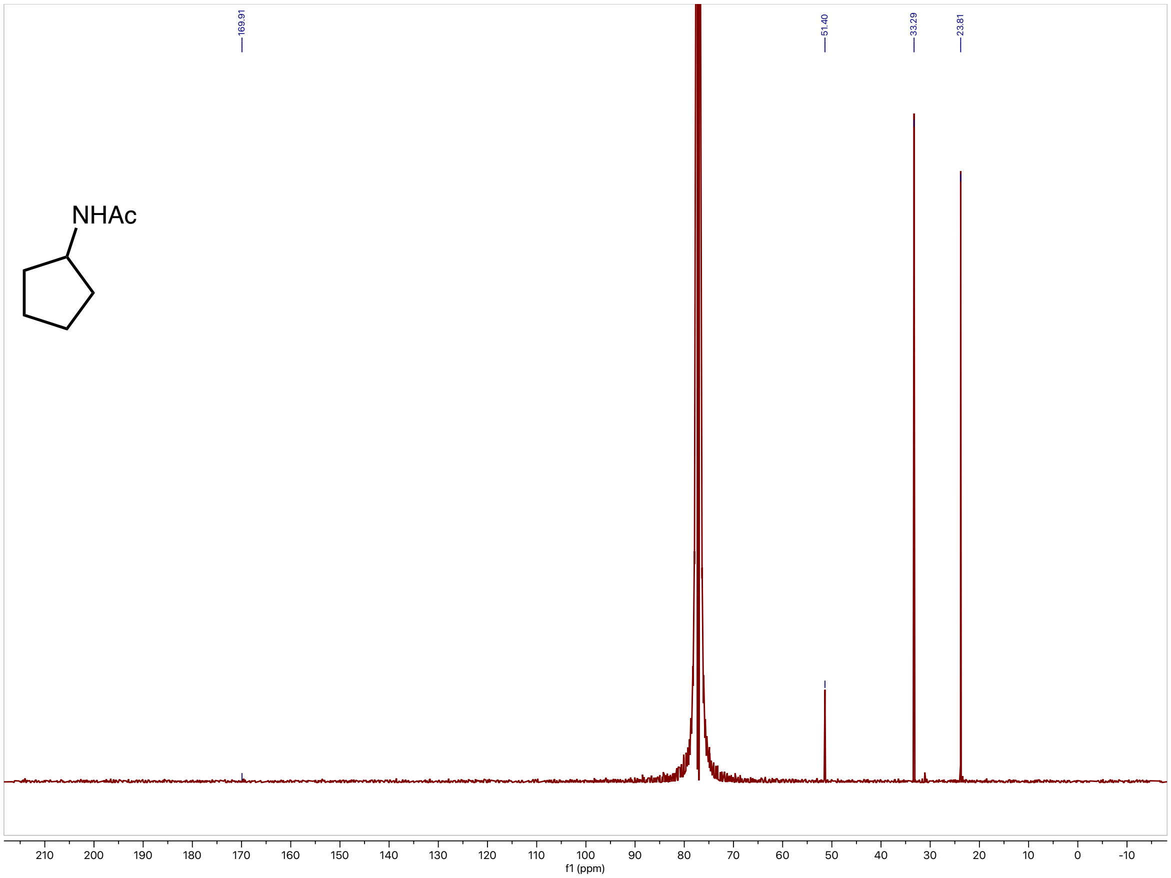

16a; ^{13}C NMR, $CDCl_3$, 125 MHz

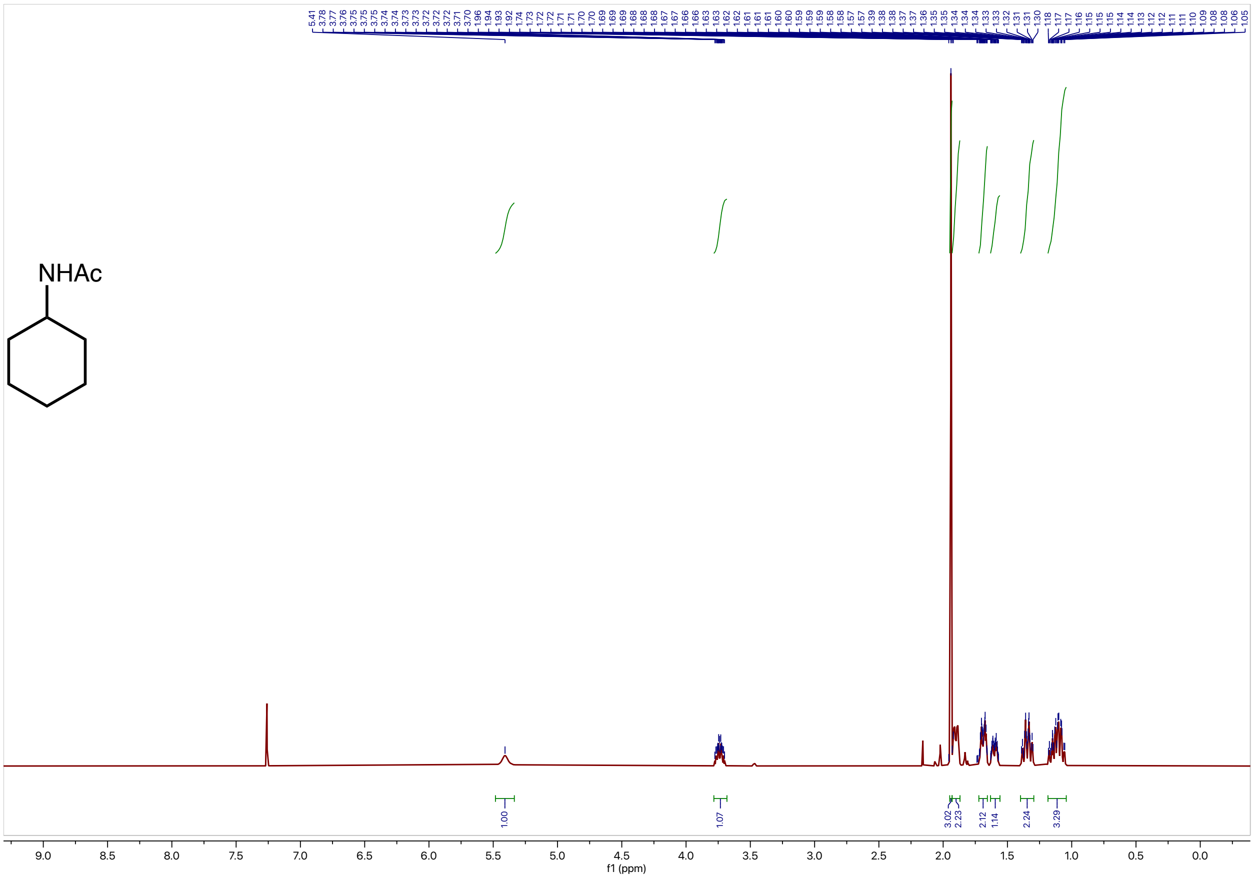

16b; ^{1}H NMR, $CDCl_3$, 500 MHz

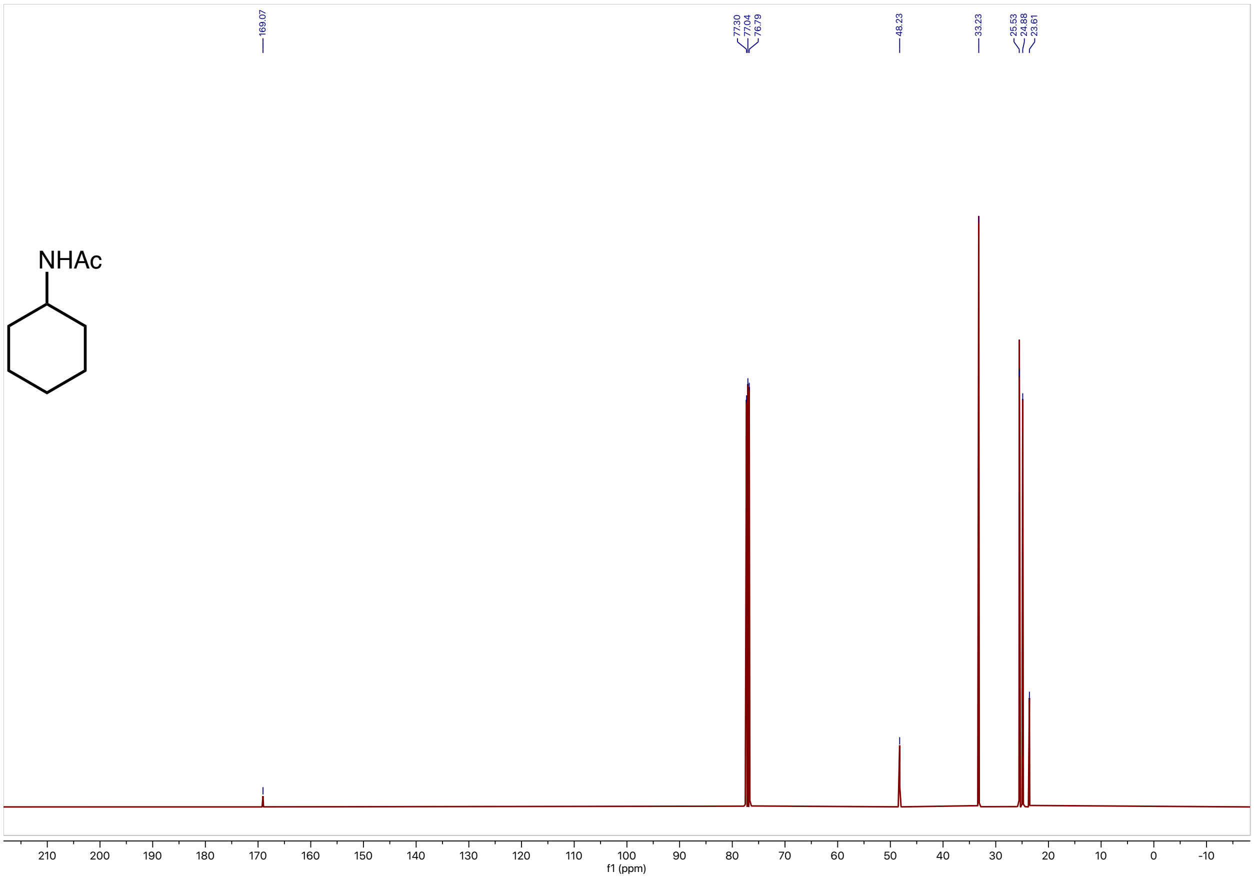

16b; ^{13}C NMR, $CDCl_3$, 125 MHz

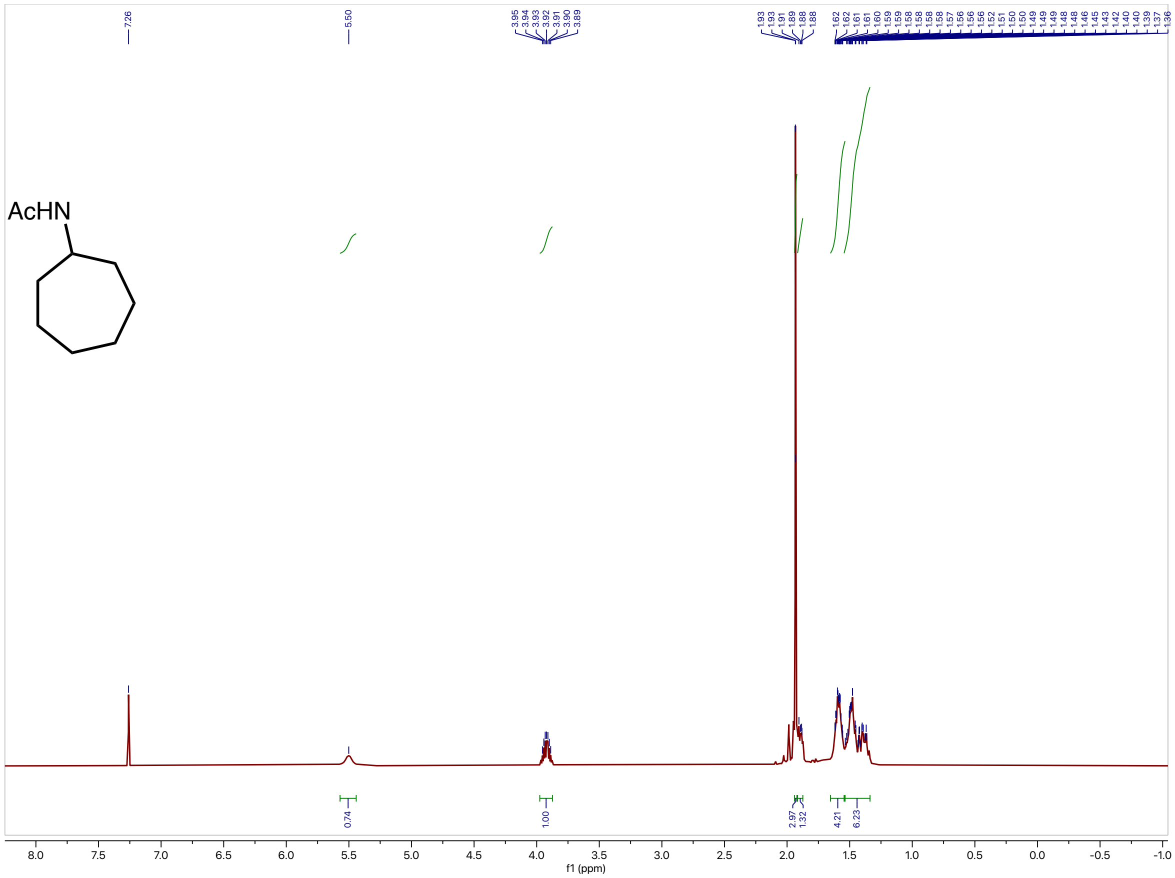

16c; ^1H NMR, $CDCl_3$, 500 MHz

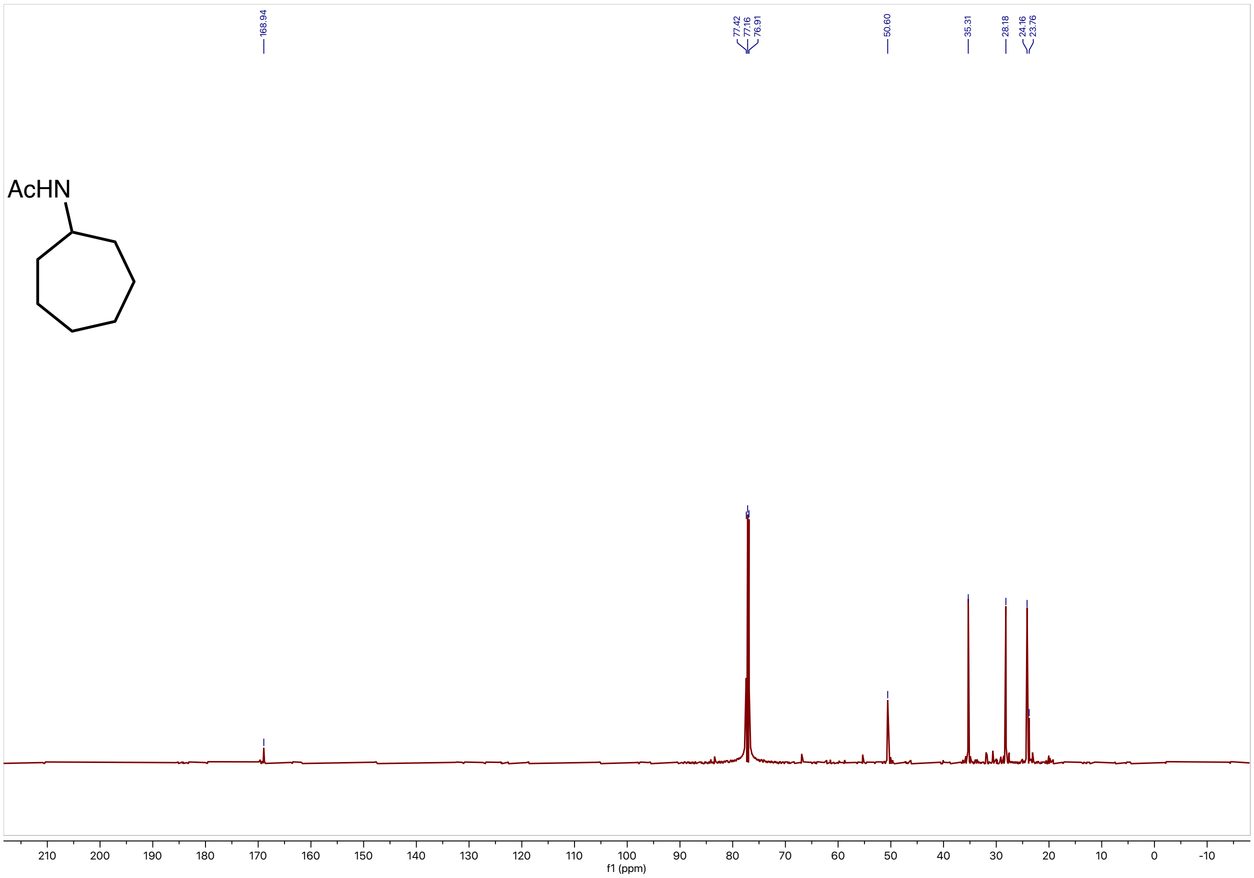

16c; ^{13}C NMR, $CDCl_3$, 125 MHz

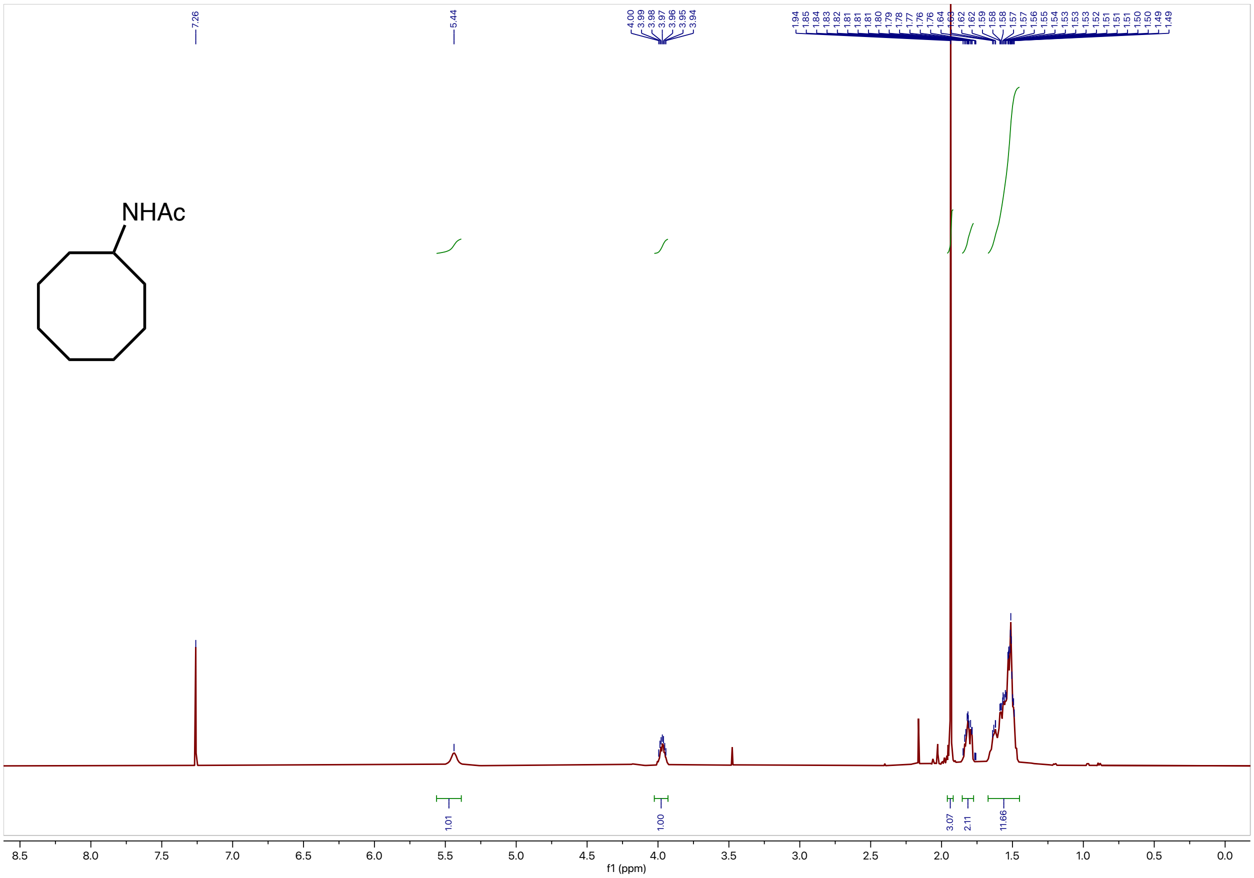

16d; ^{1}H NMR, $CDCl_3$, 500 MHz

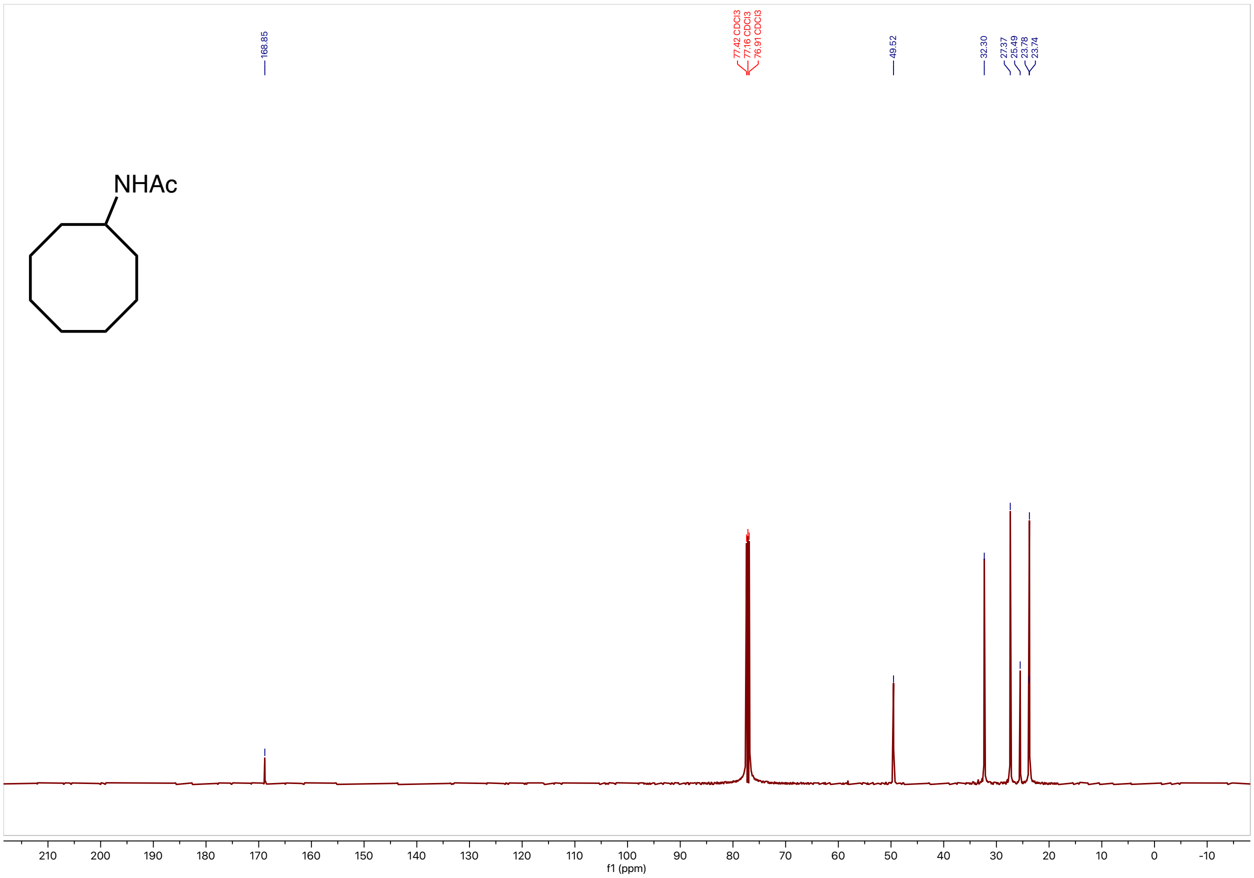

16d; ^{13}C NMR, $CDCl_3$, 125 MHz

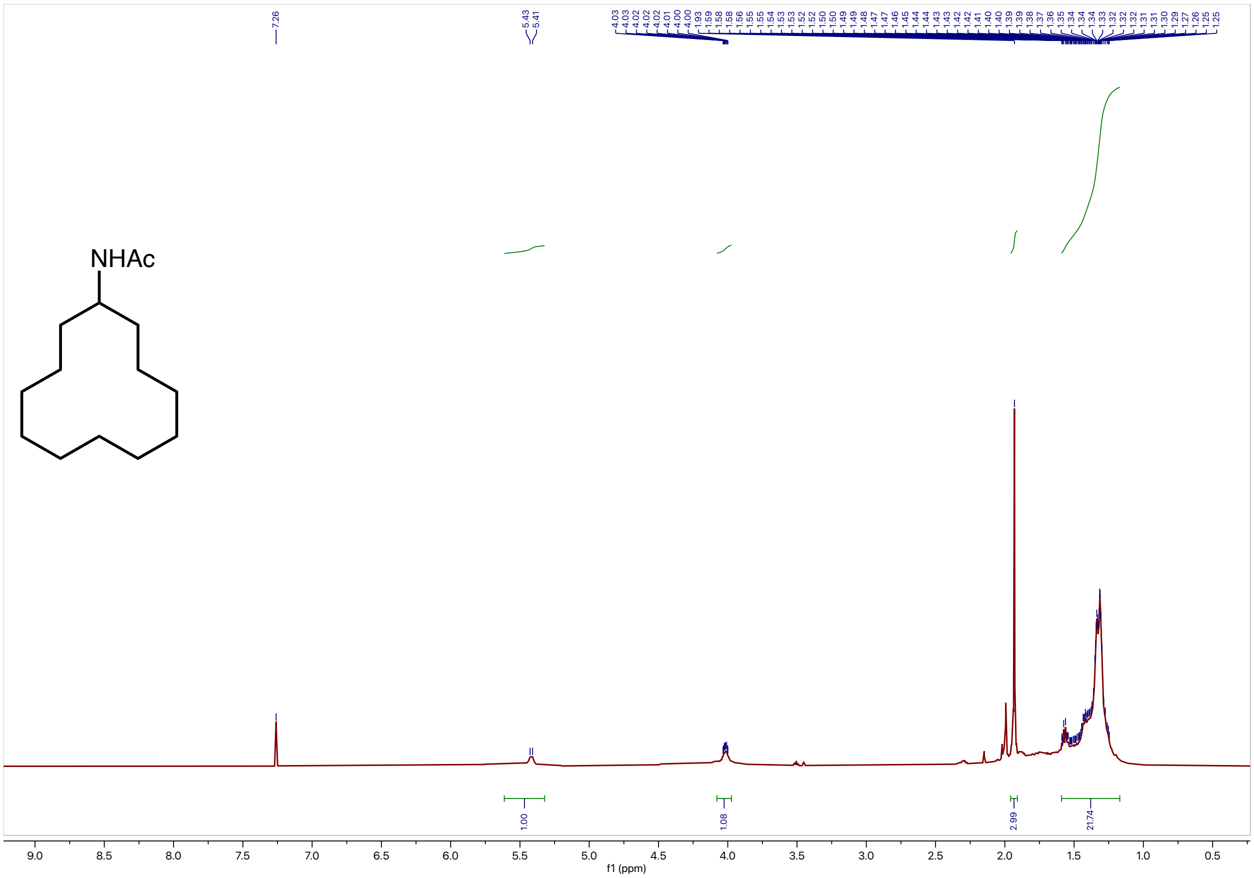

16e; ^{1}H NMR, $CDCl_3$, 500 MHz

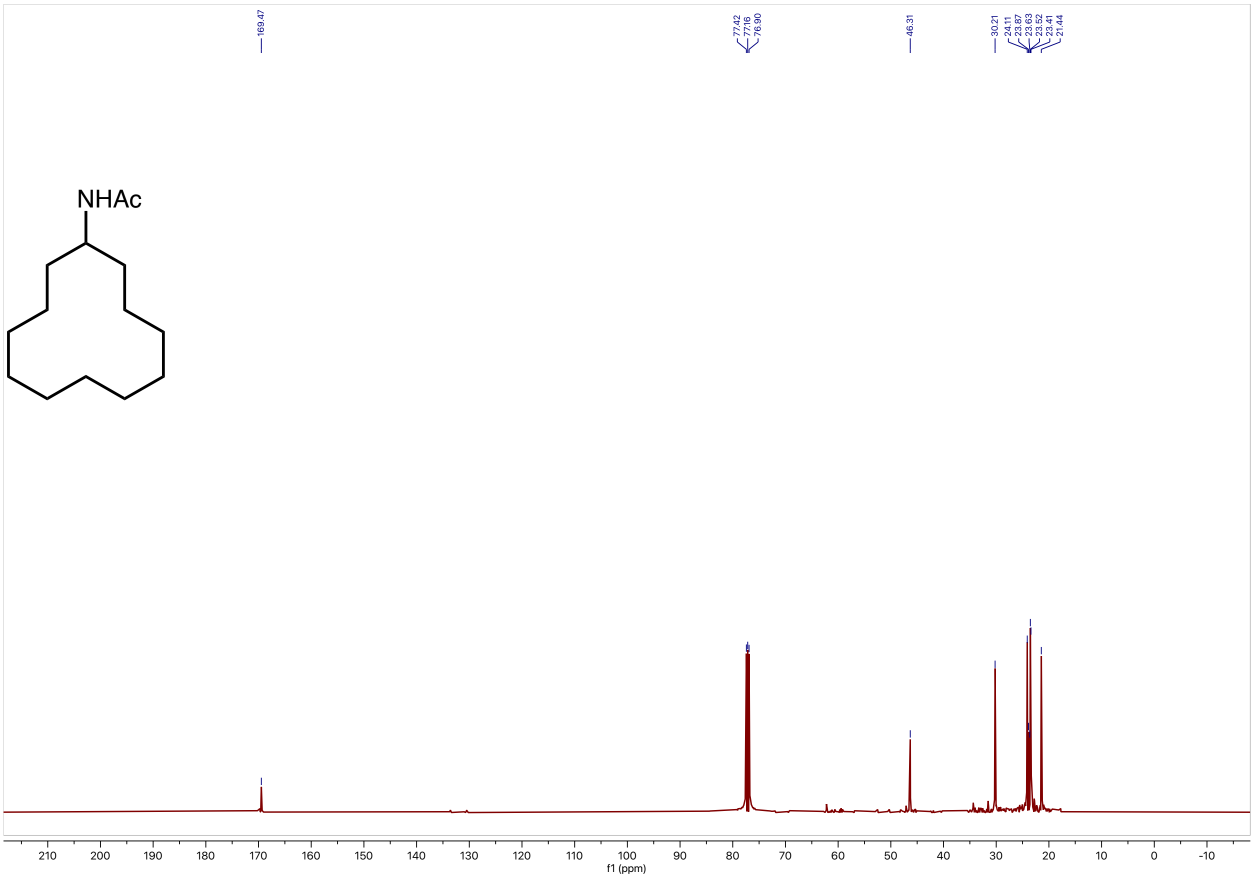

16e; ^{13}C NMR, $CDCl_3$, 125 MHz

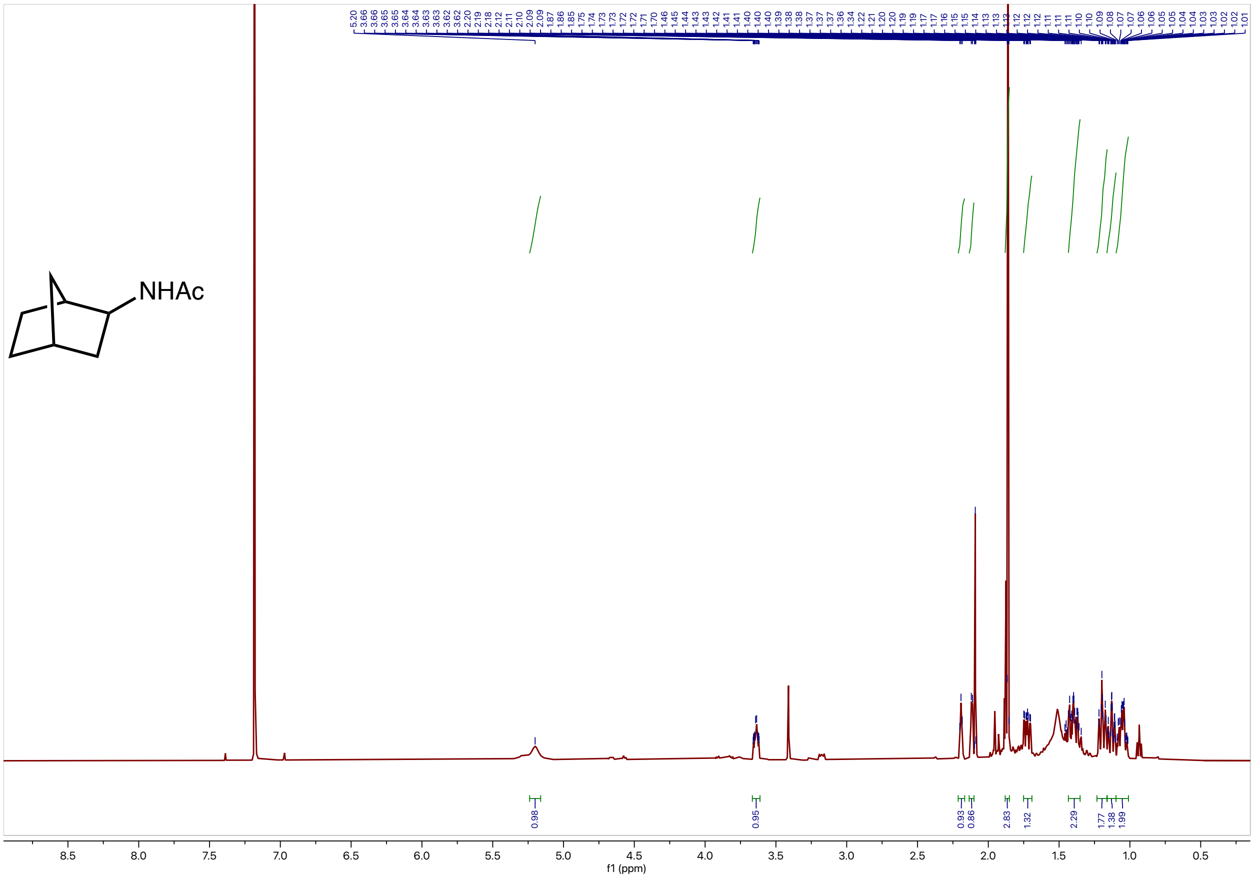

17; ^{1}H NMR, $CDCl_3$, 500 MHz

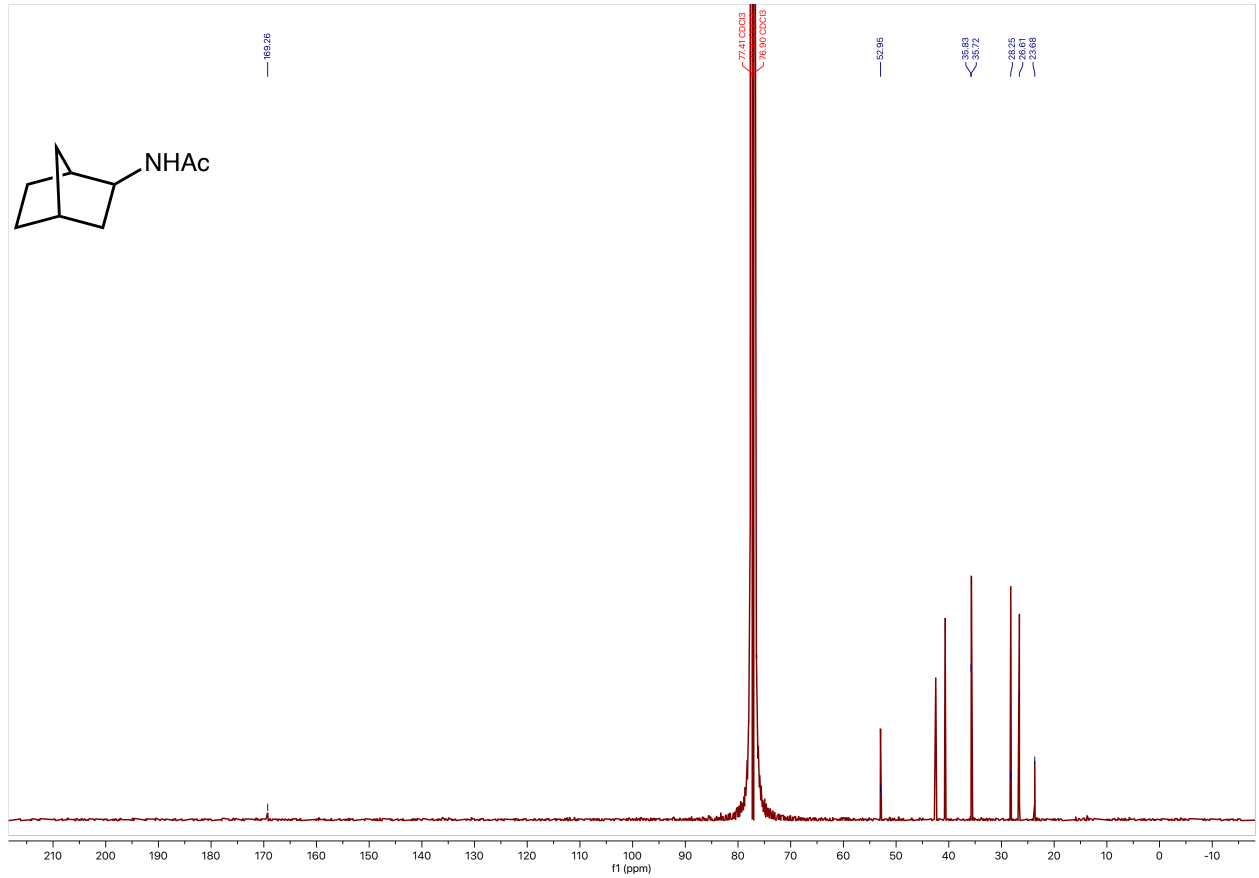

17; ^{13}C NMR, $CDCl_3$, 125 MHz

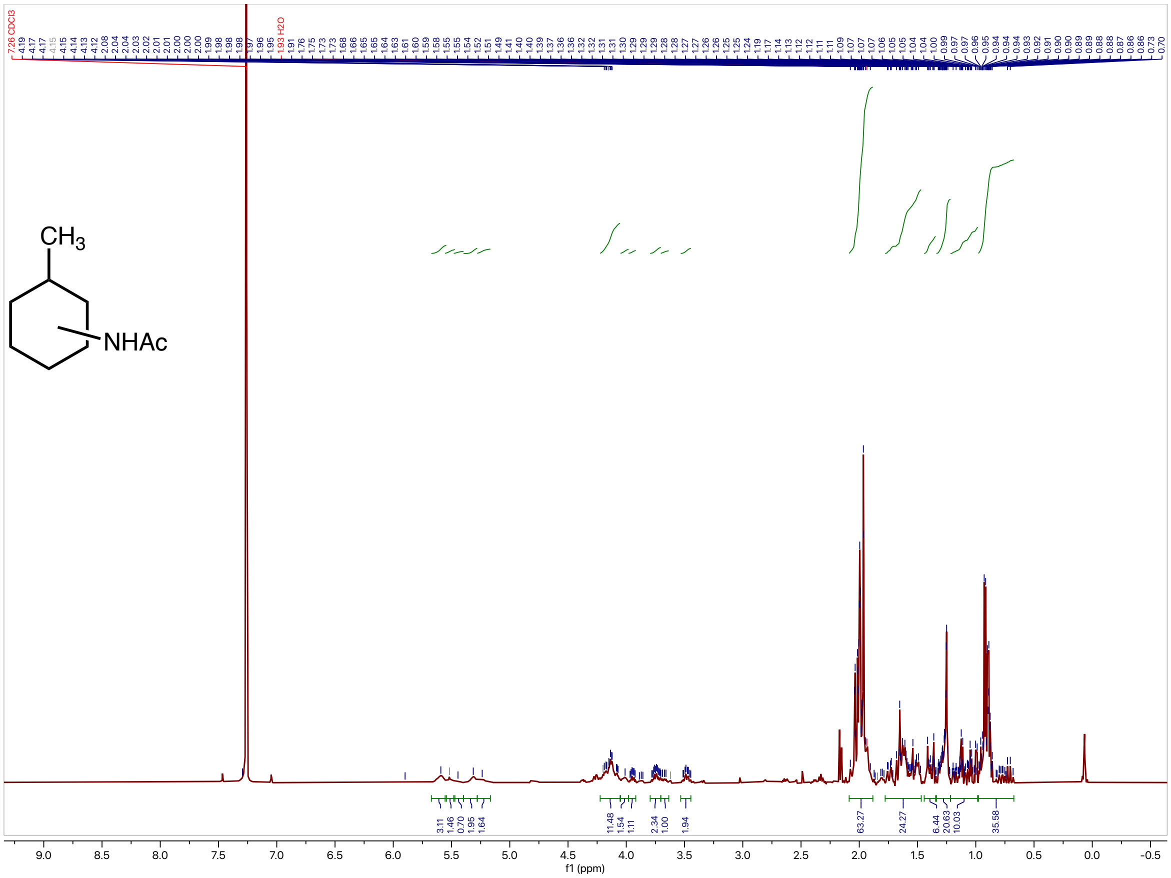

19; ^{1}H NMR, $CDCl_3$, 500 MHz

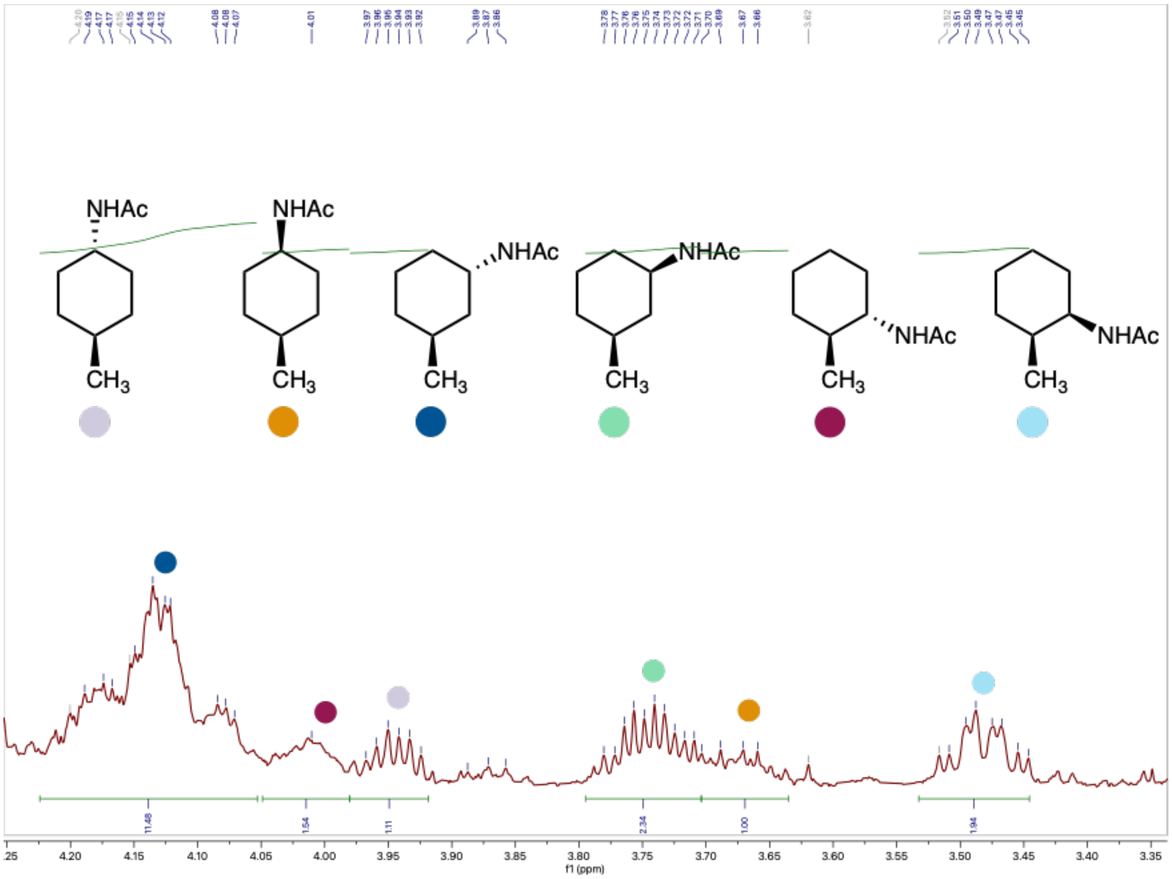

19 zoom; ^{1}H NMR, $CDCl_3$, 500 MHz (ref. *66)*
*Only relative stereochemistry is assigned

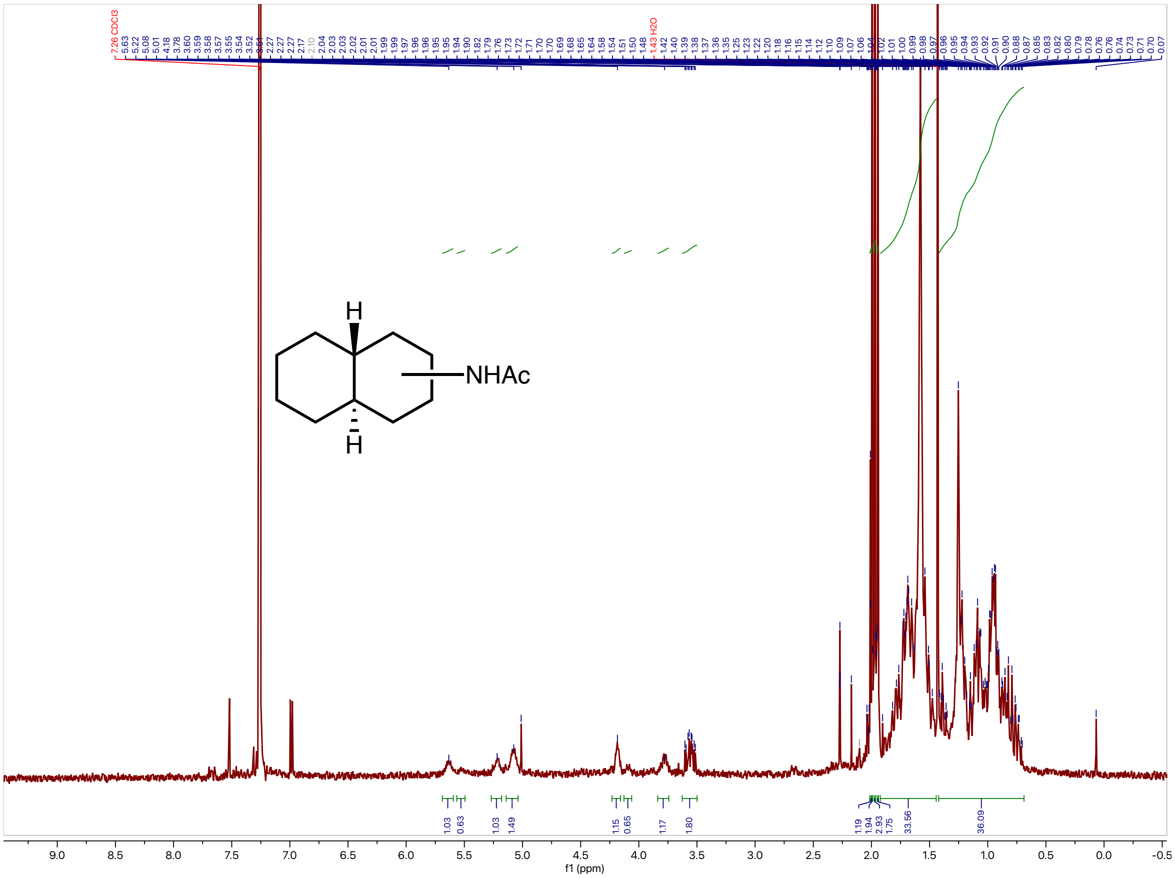

20; ^{1}H NMR, $CDCl_3$, 500 MHz

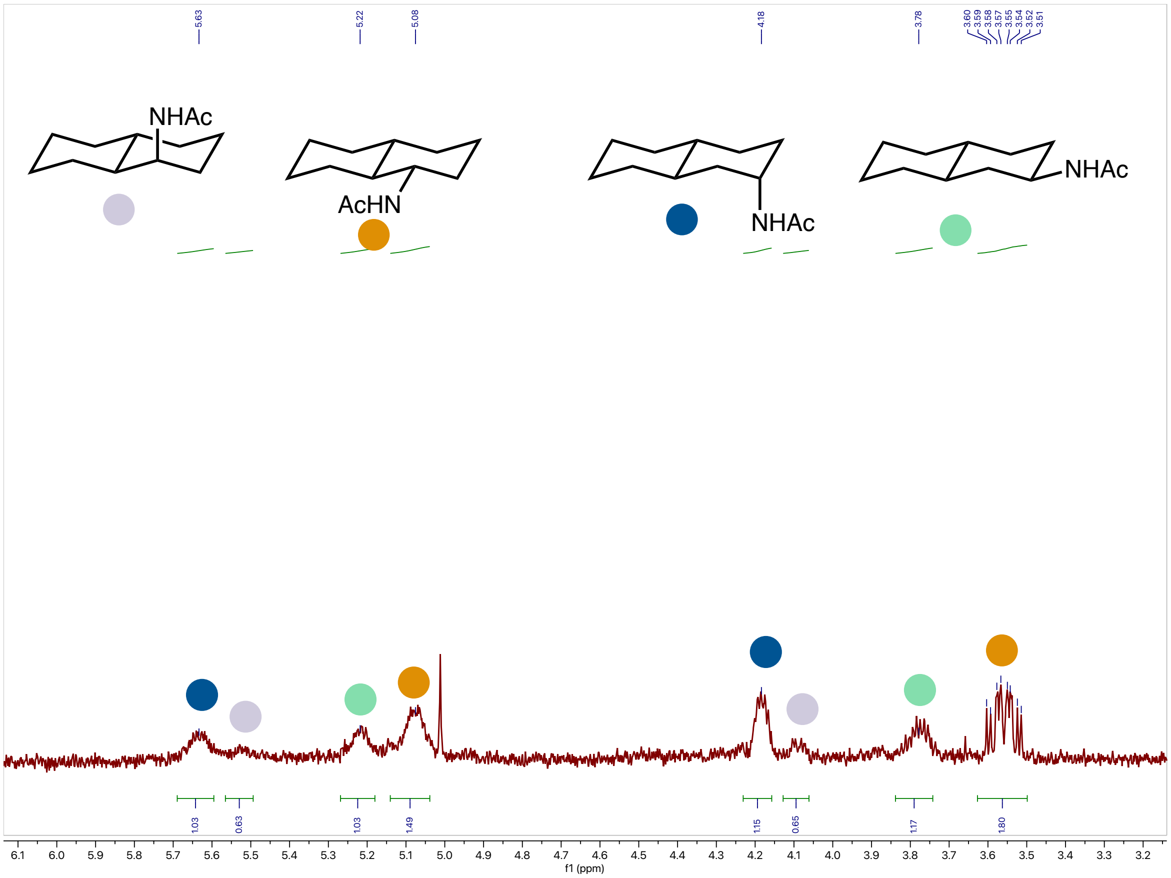

20 zoom; ^{1}H NMR, $CDCl_3$, 500 MHz (ref. *70*)

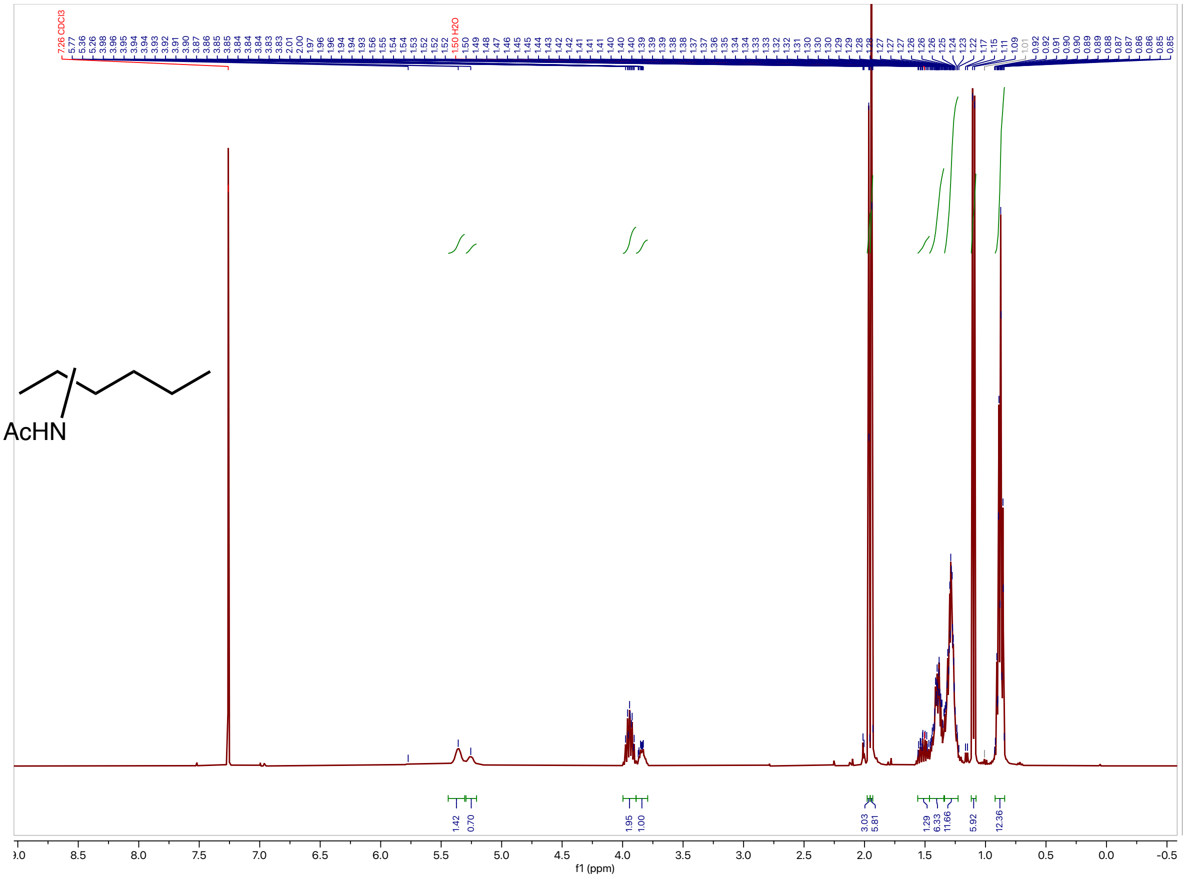

21; ^{1}H NMR, $CDCl_3$, 400 MHz

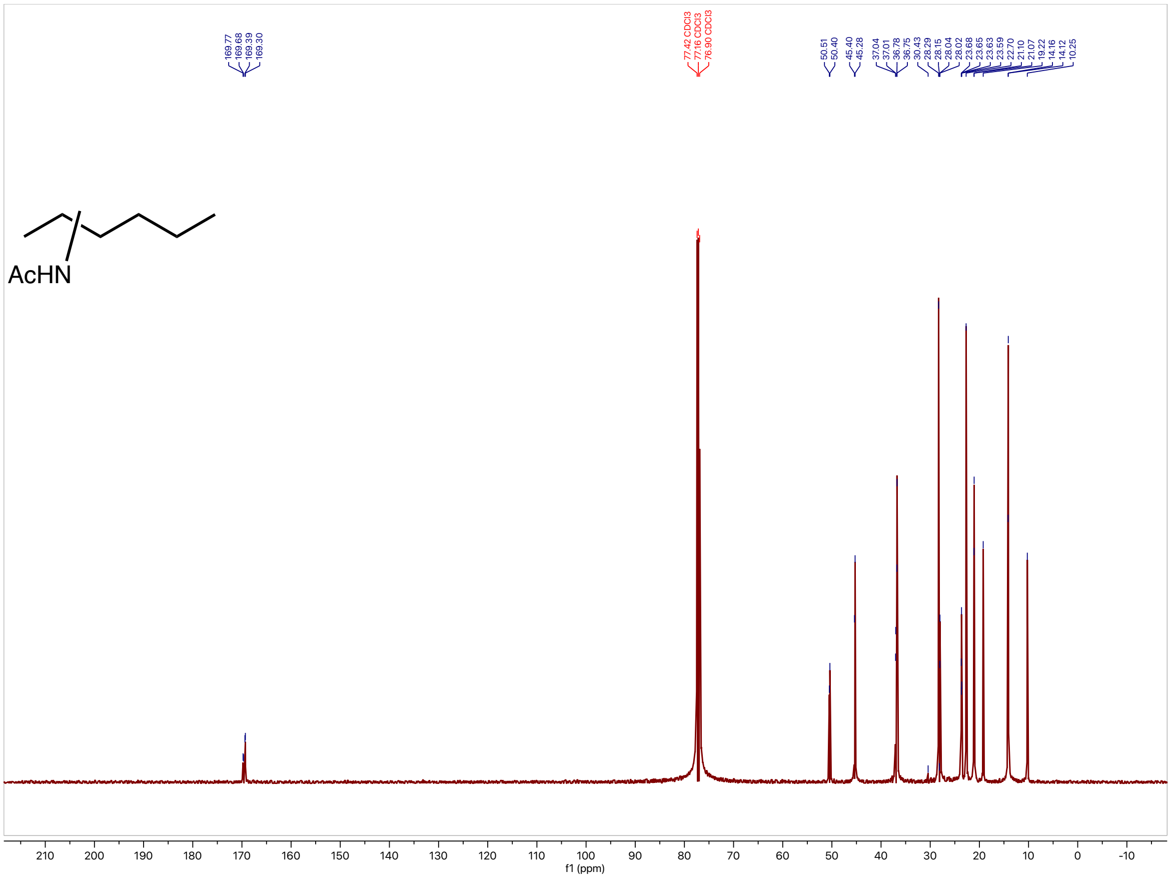

21; ^{13}C NMR, $CDCl_3$, 100 MHz

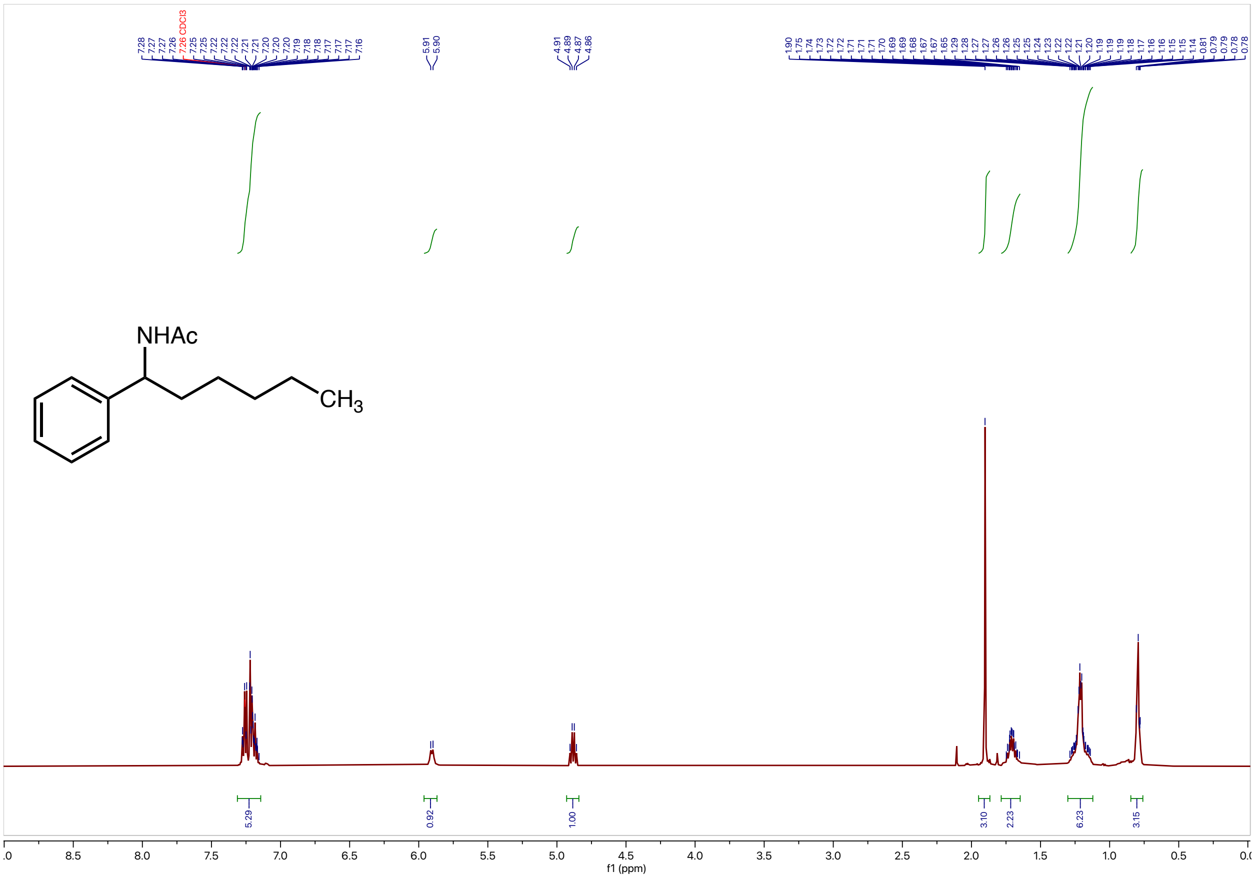

22; ^{1}H NMR, $CDCl_3$, 400 MHz

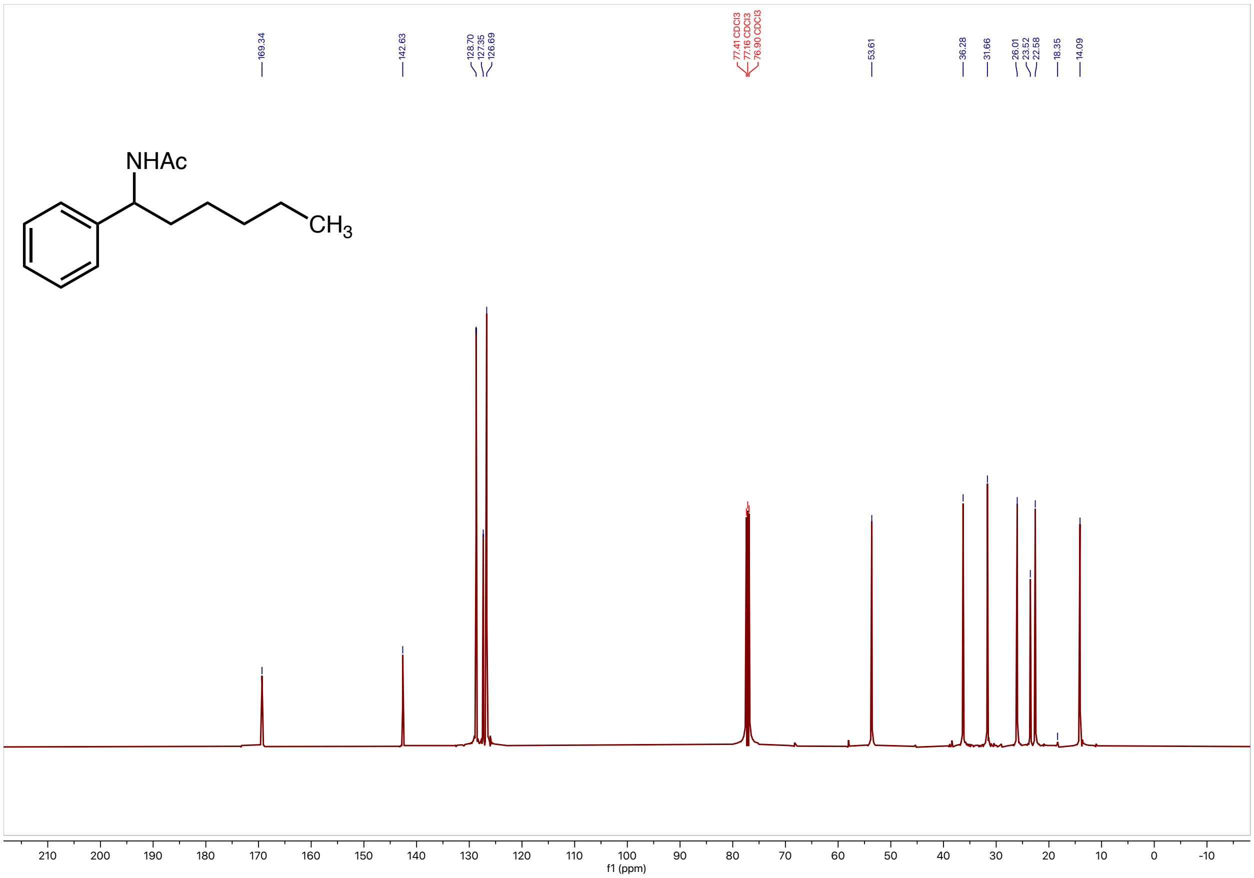

22; ^{13}C NMR, $CDCl_3$, 100 MHz

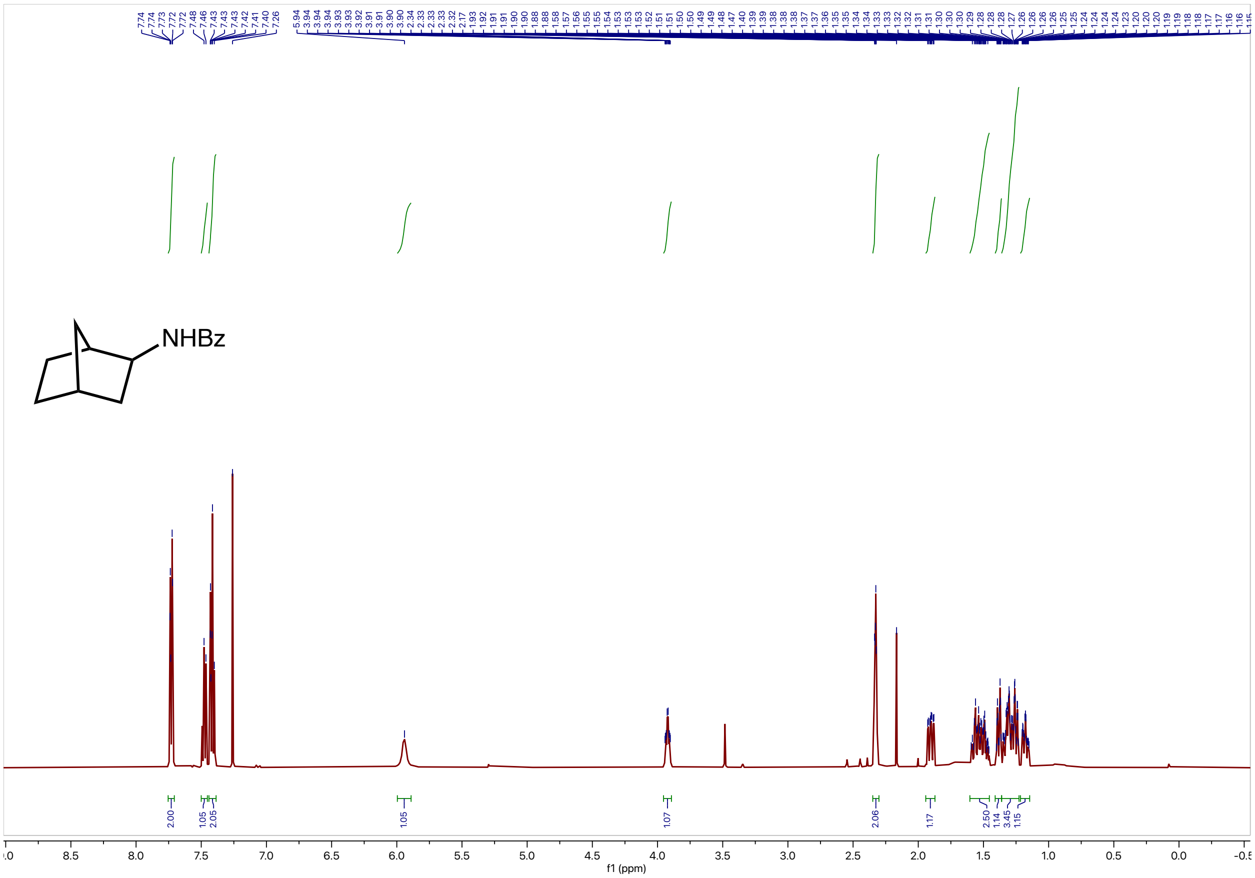

23; ^{1}H NMR, $CDCl_3$, 400 MHz

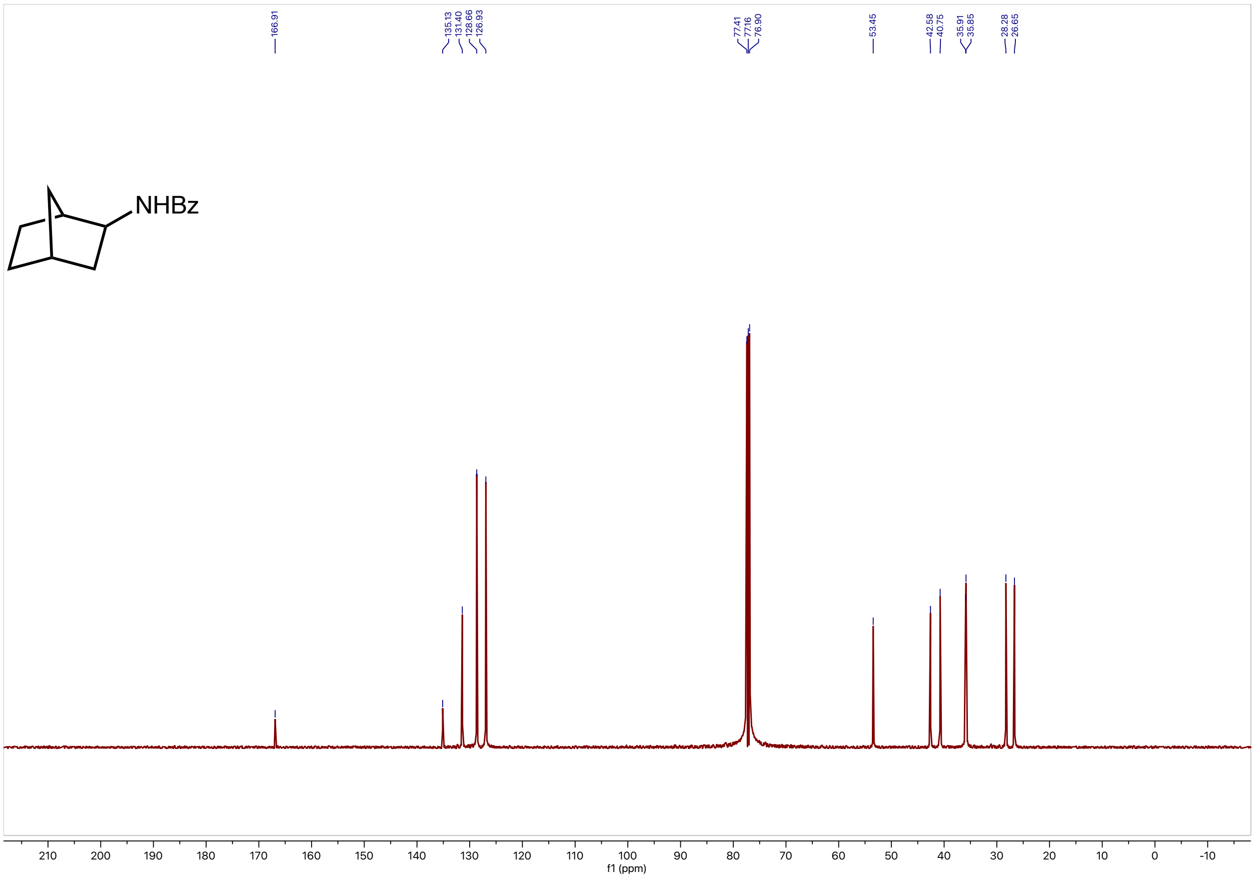

23; ^{13}C NMR, $CDCl_3$, 100 MHz

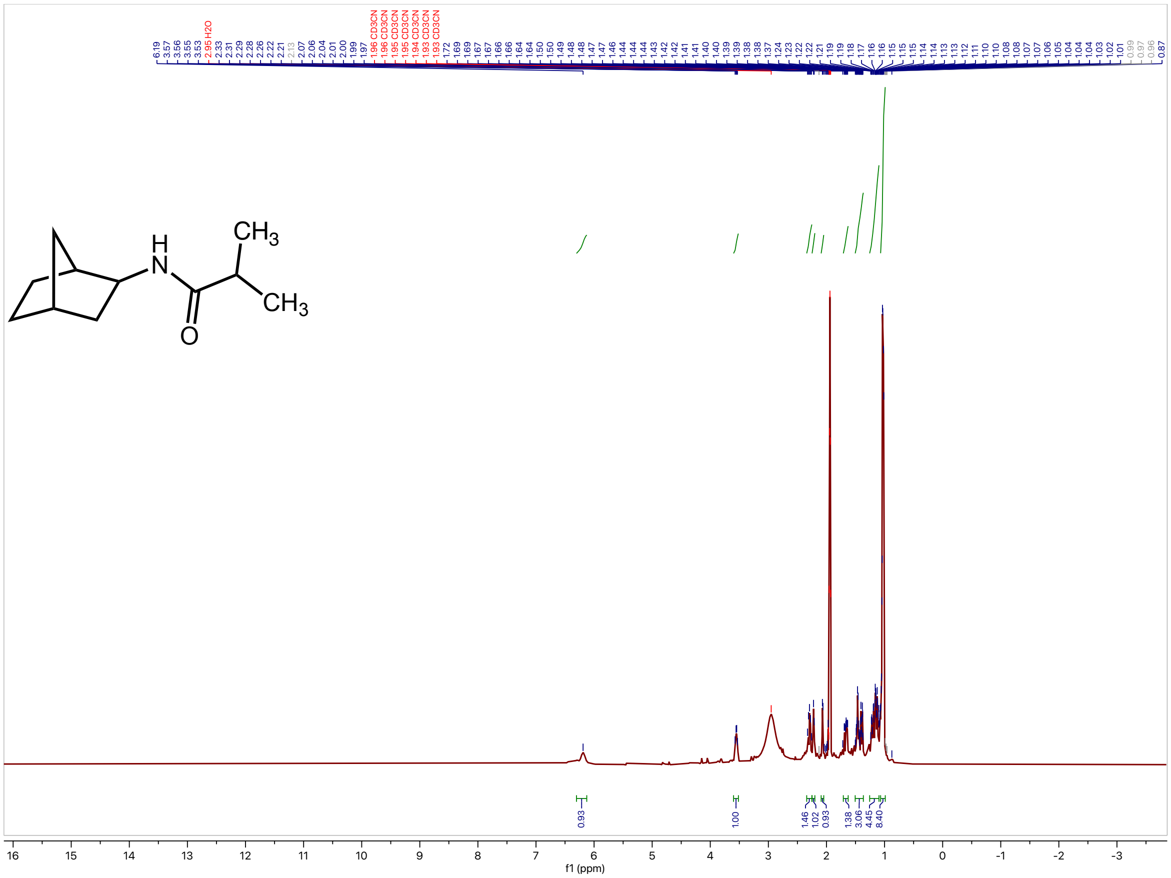

24; ^{1}H NMR, CD_3CN, 500 MHz

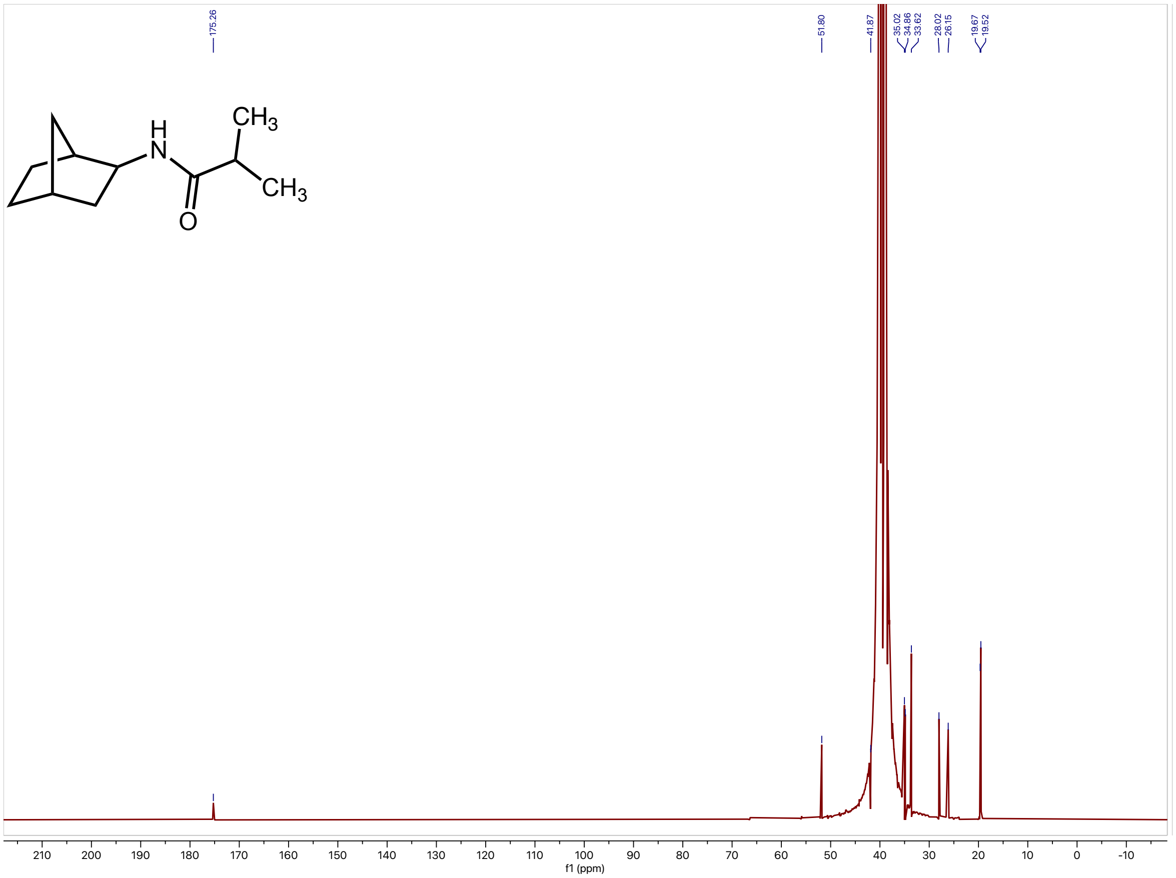

24; ^{13}C NMR, CD_3CN, 125 MHz

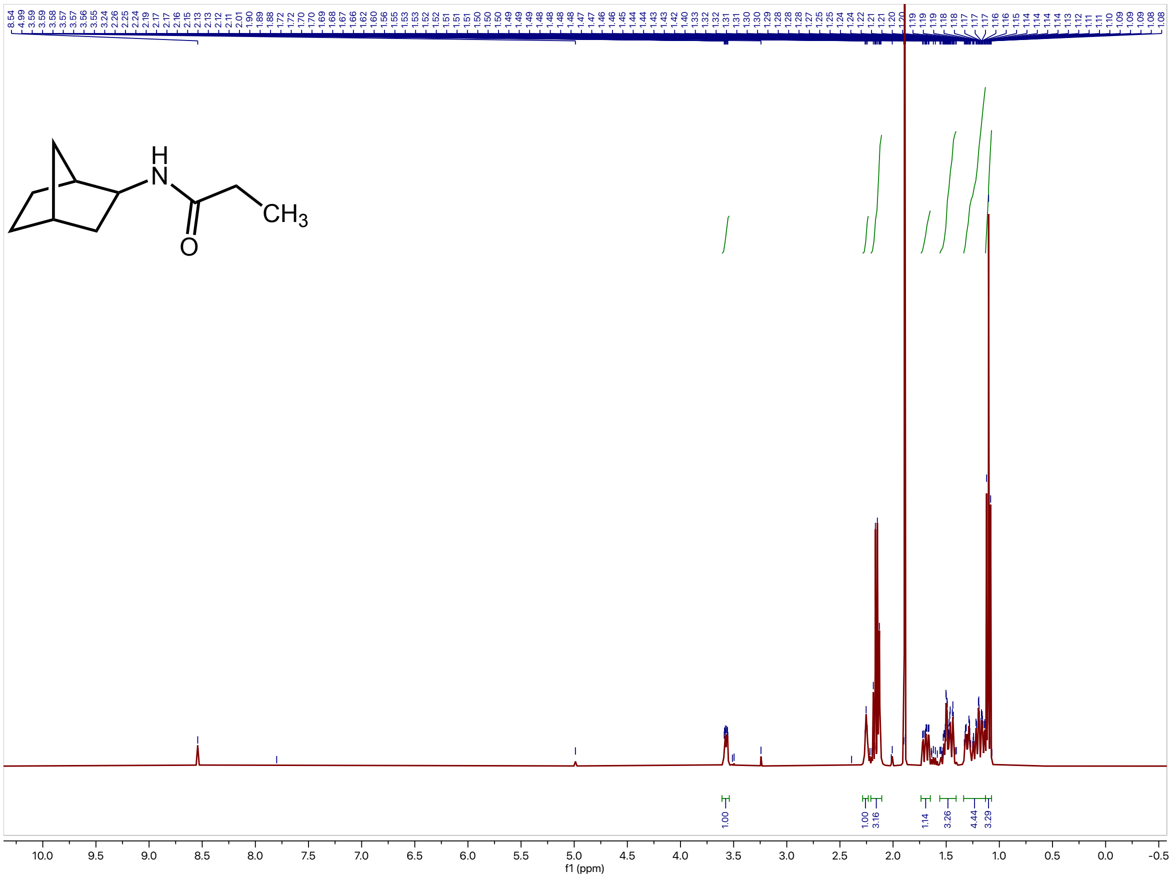

25; ^{1}H NMR, CD_3OD, 400 MHz

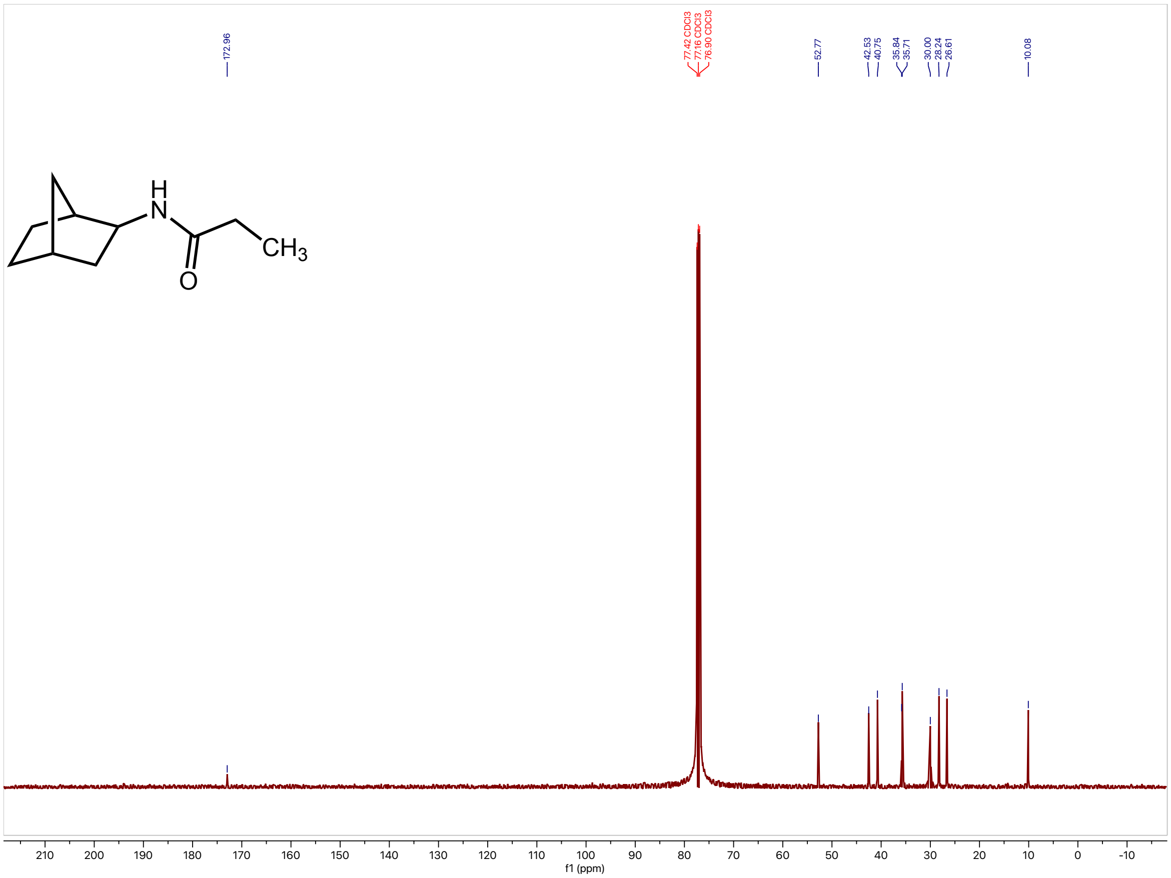

25; ^{13}C NMR, $CDCl_3$, 125 MHz

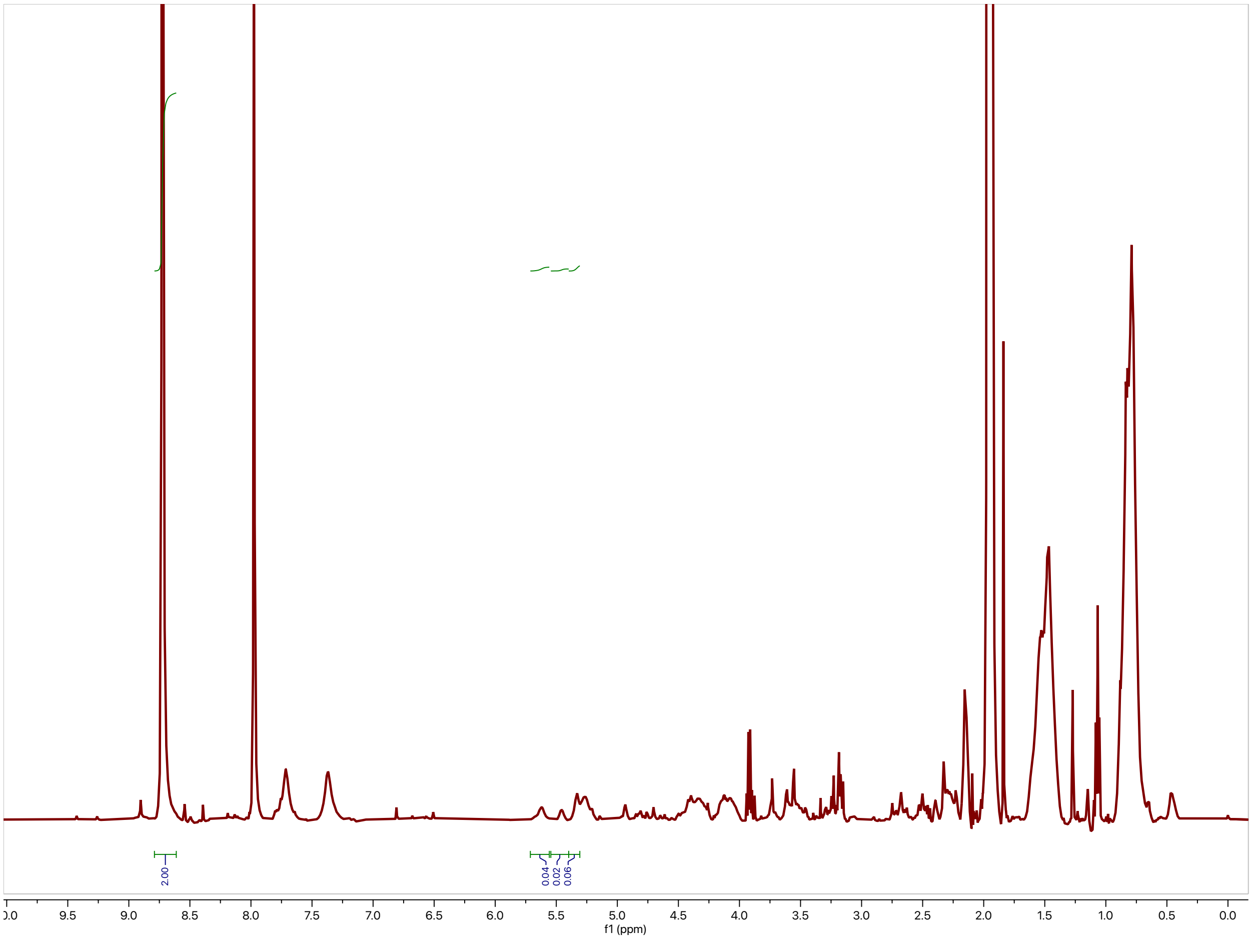

Reaction of kerosene; ^1H NMR, 500 MHz, $(CD_3)_2CO$

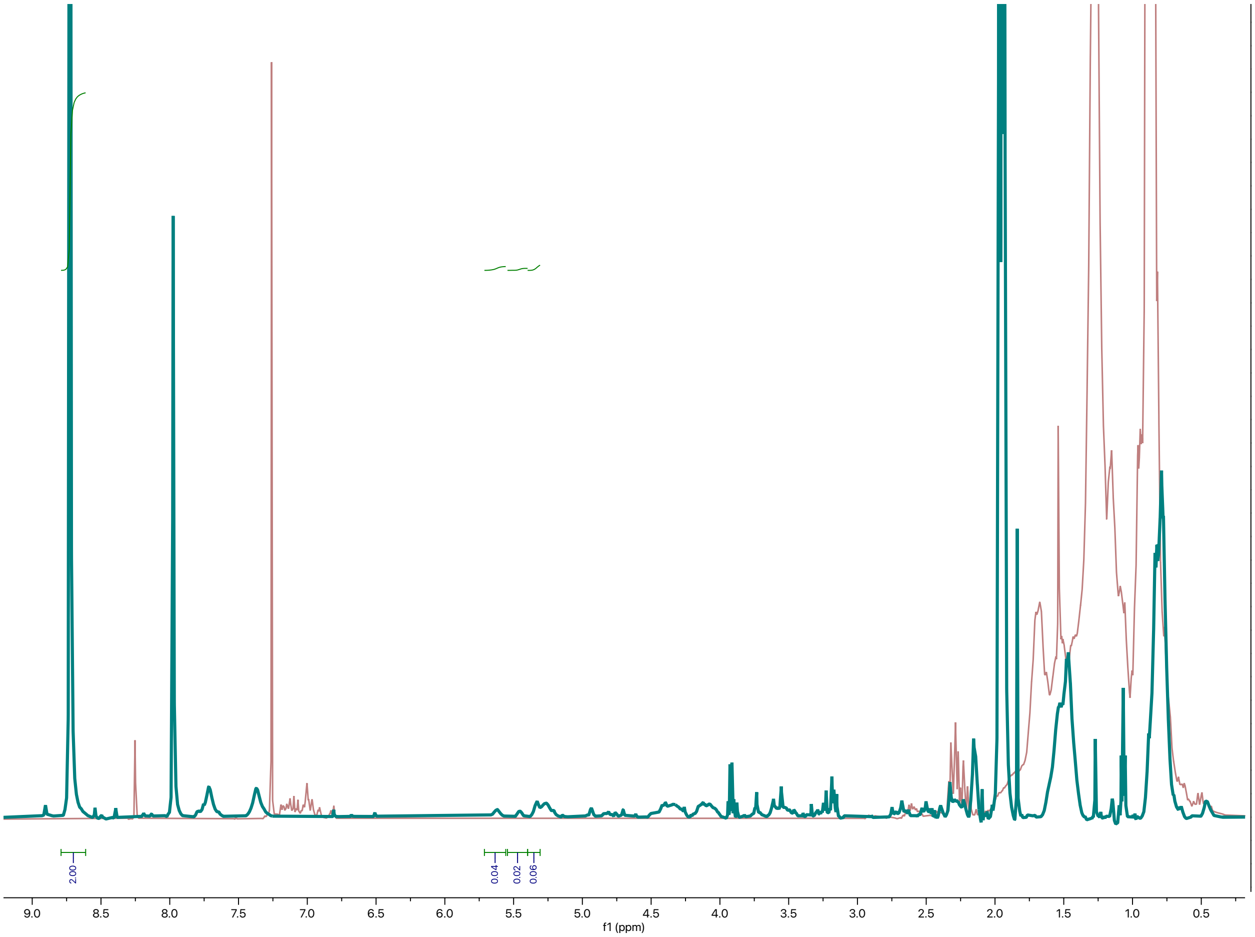

Reaction of kerosene; ^{1}H NMR, 500 MHz, $(CD_3)_2CO$ (green) overlaid with kerosene starting material (maroon)

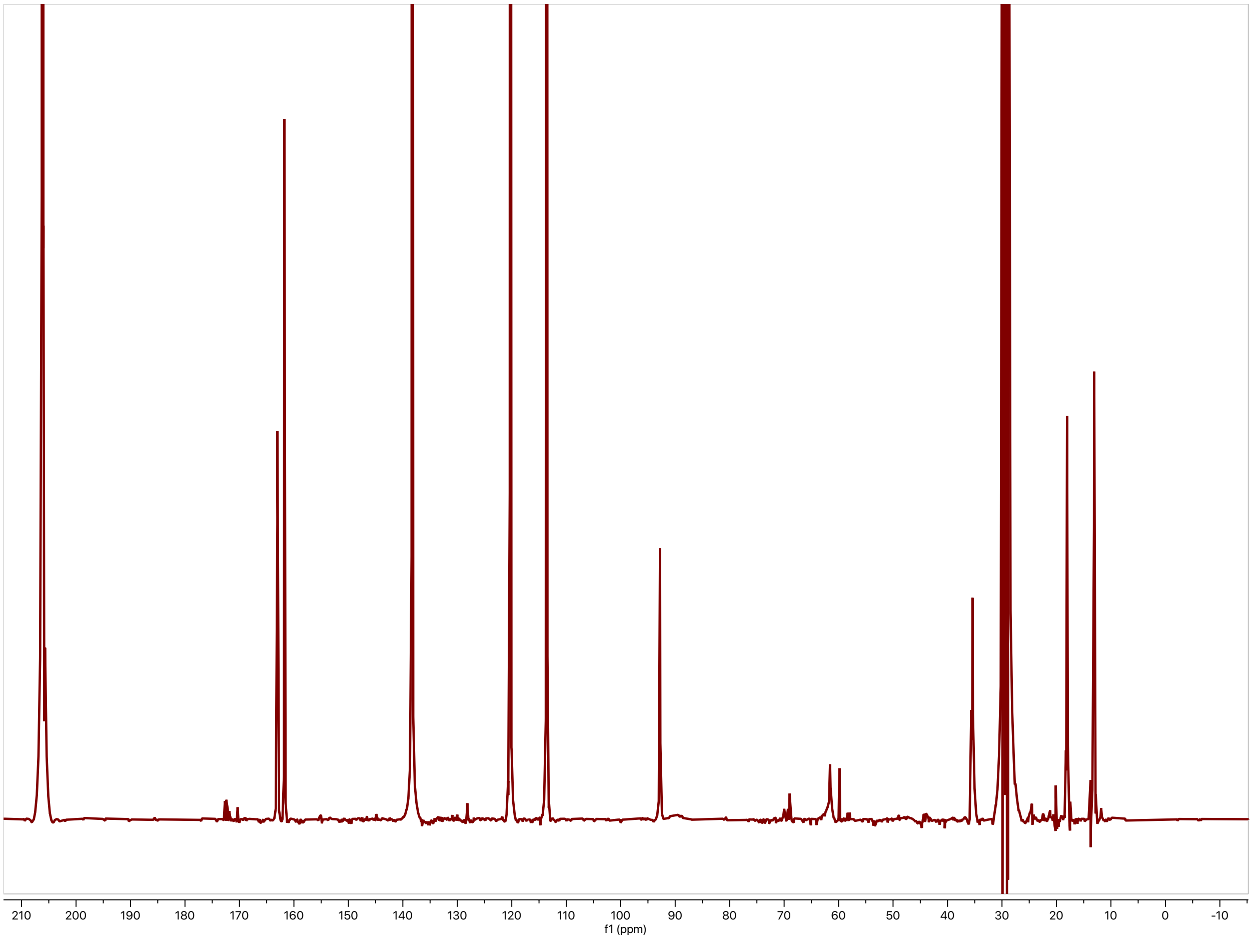

Reaction of kerosene; ^{13}C NMR, 125 MHz, $(CD_3)_2CO$

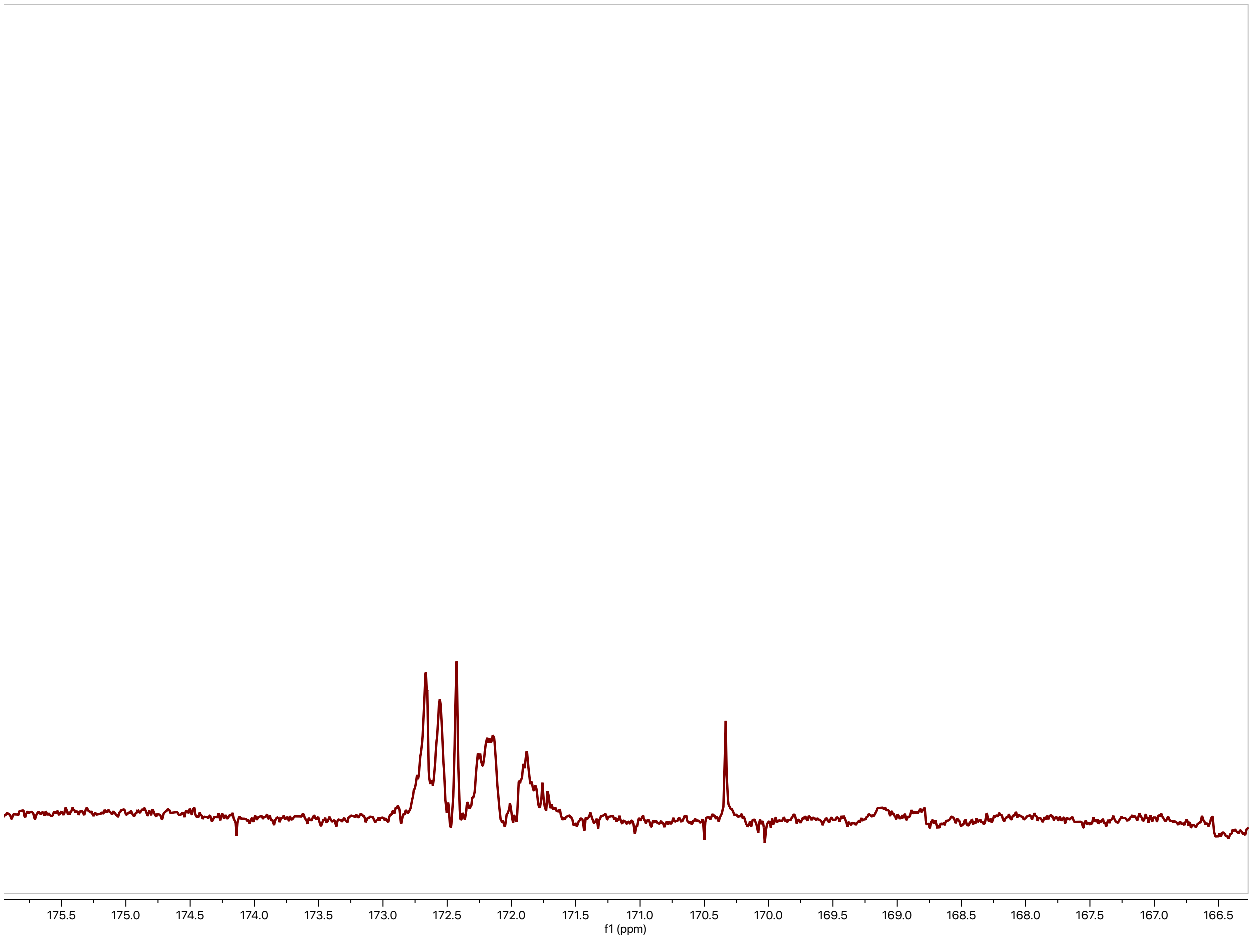

Reaction of kerosene; ^{13}C NMR, 125 MHz, $(CD_3)_2CO$ zoom

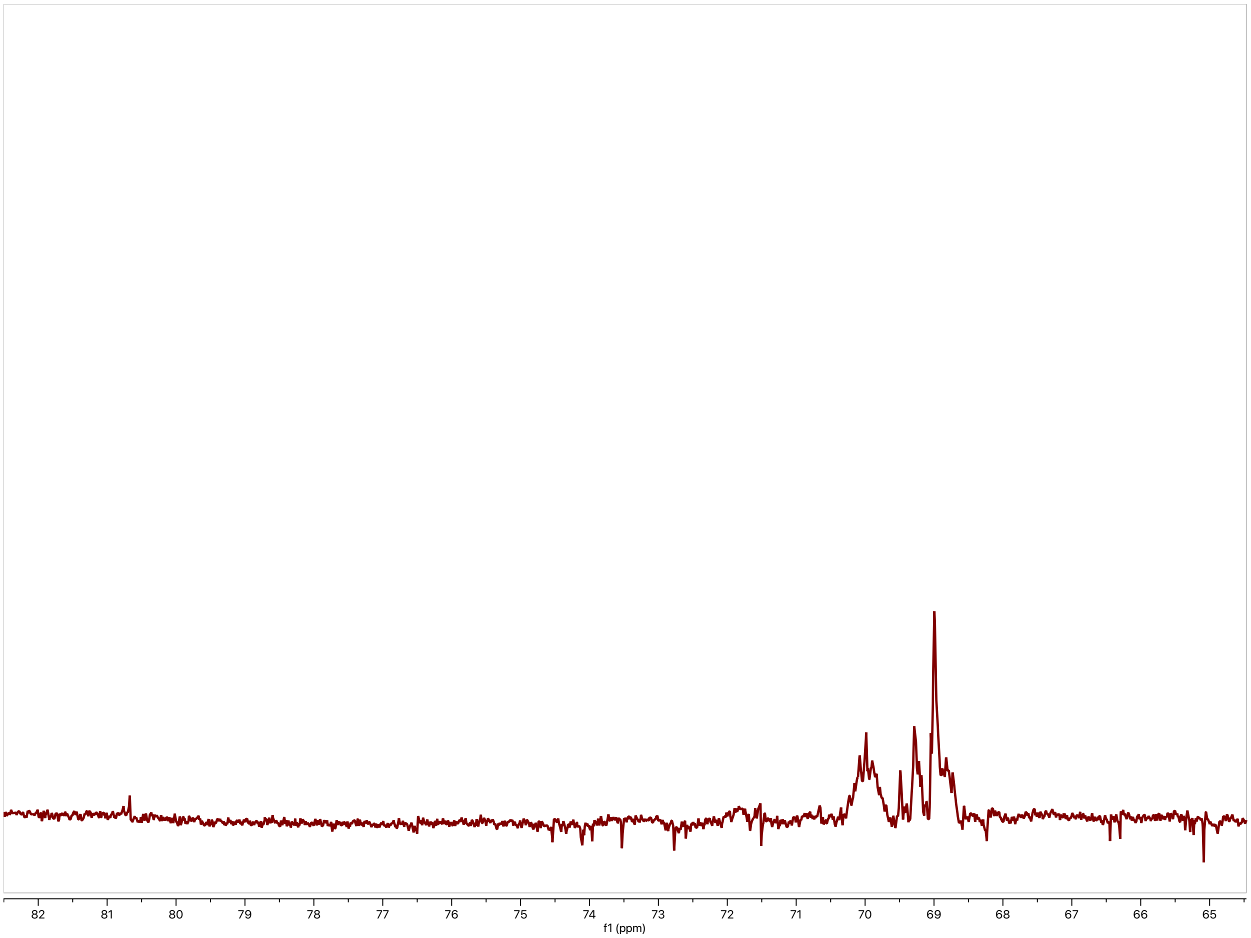

Reaction of kerosene; ^{13}C NMR, 125 MHz, $(CD_3)_2CO$ zoom

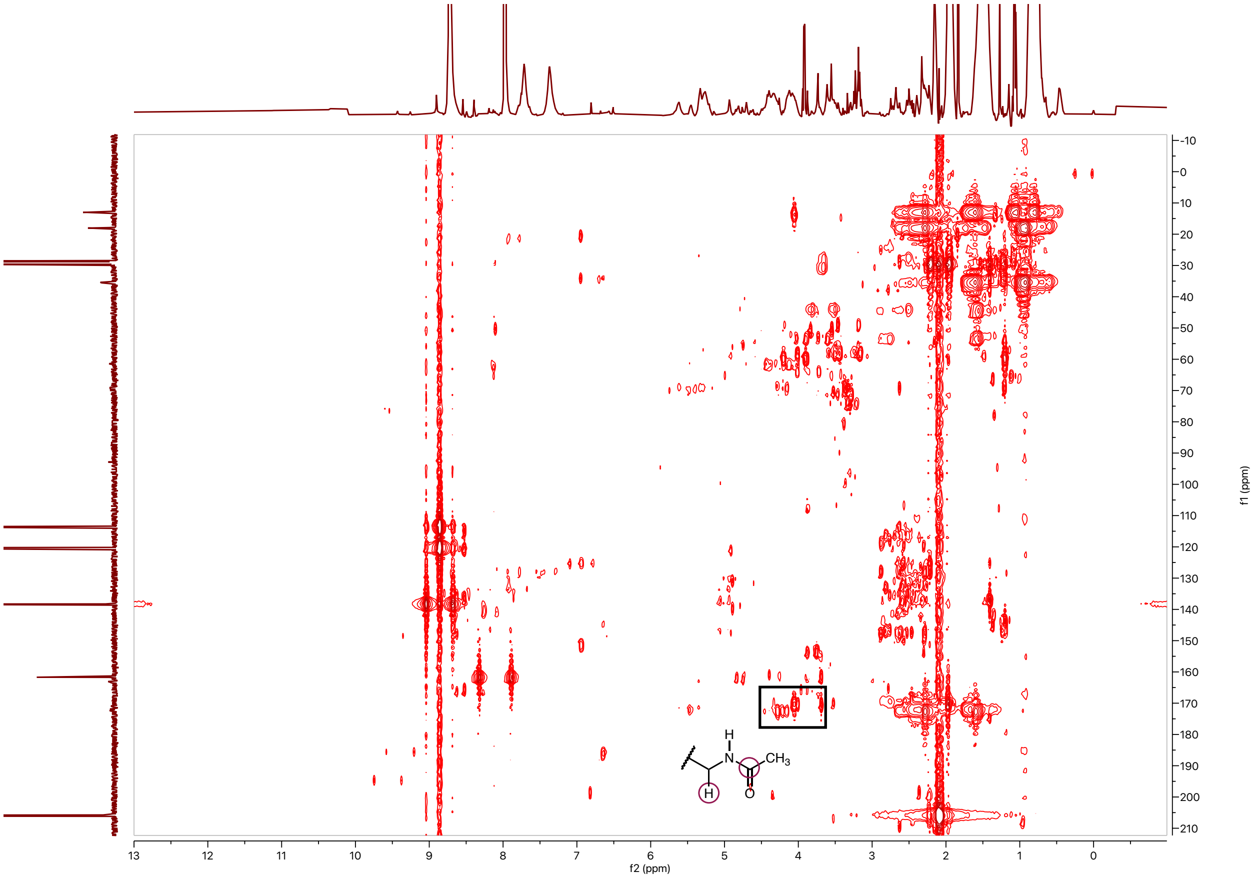

Reaction of kerosene; ^{1}H-^{13}C HMBC NMR, $(CD_3)_2CO$

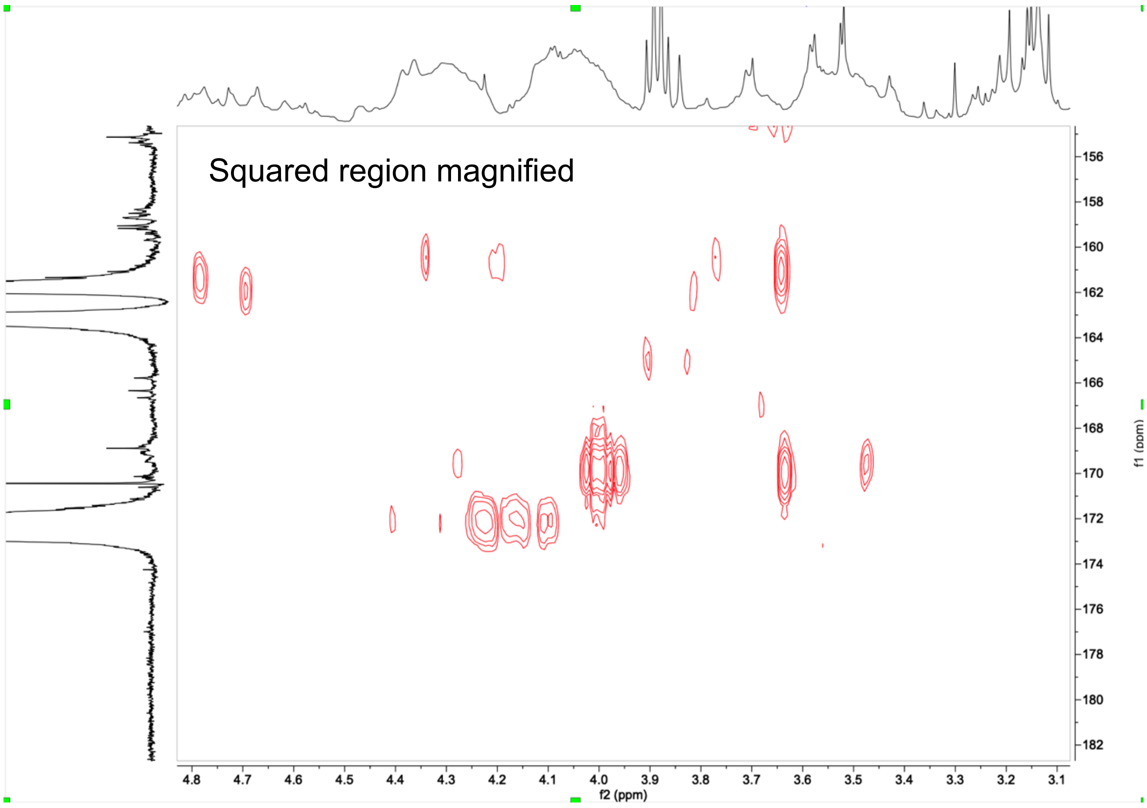

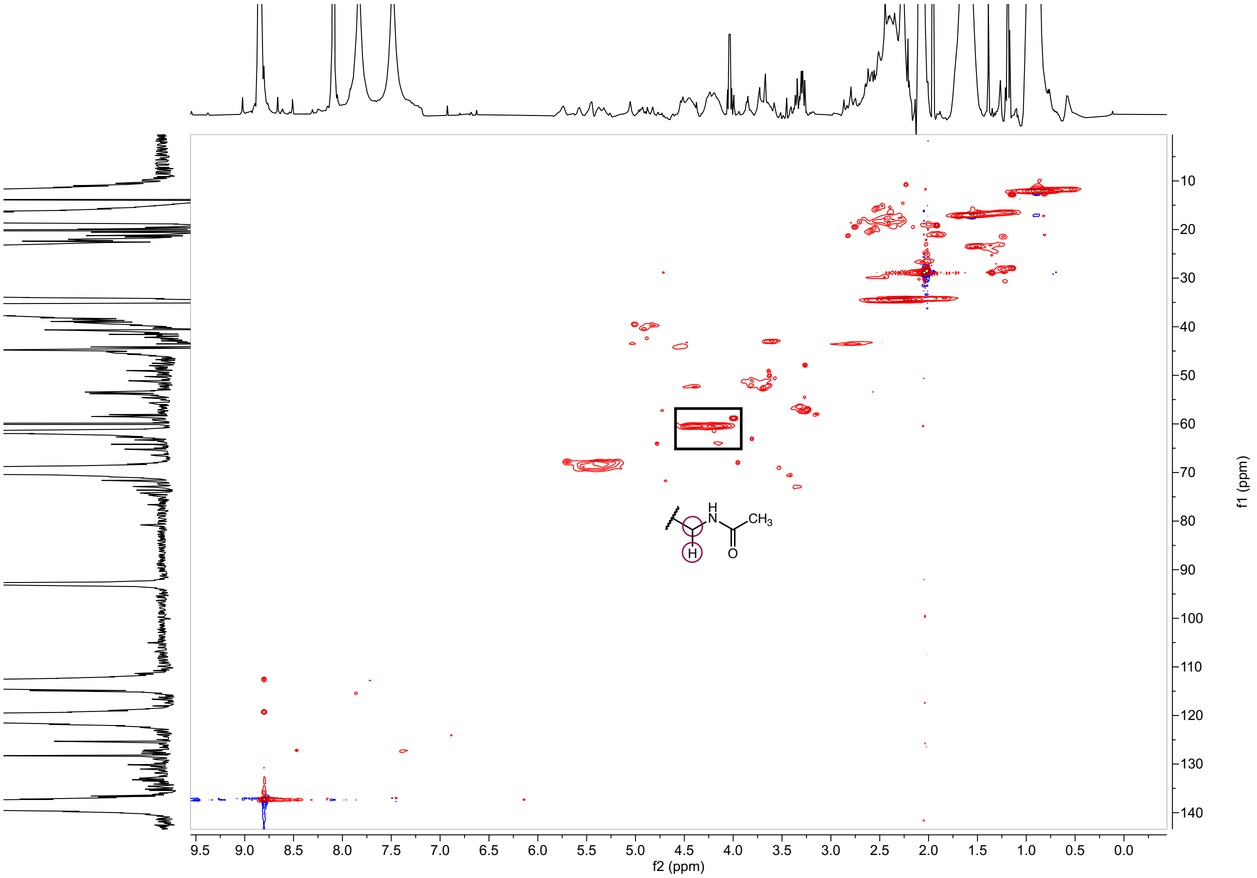

Reaction of kerosene; ^{1}H-^{13}C HSQC NMR, $(CD_3)_2CO$

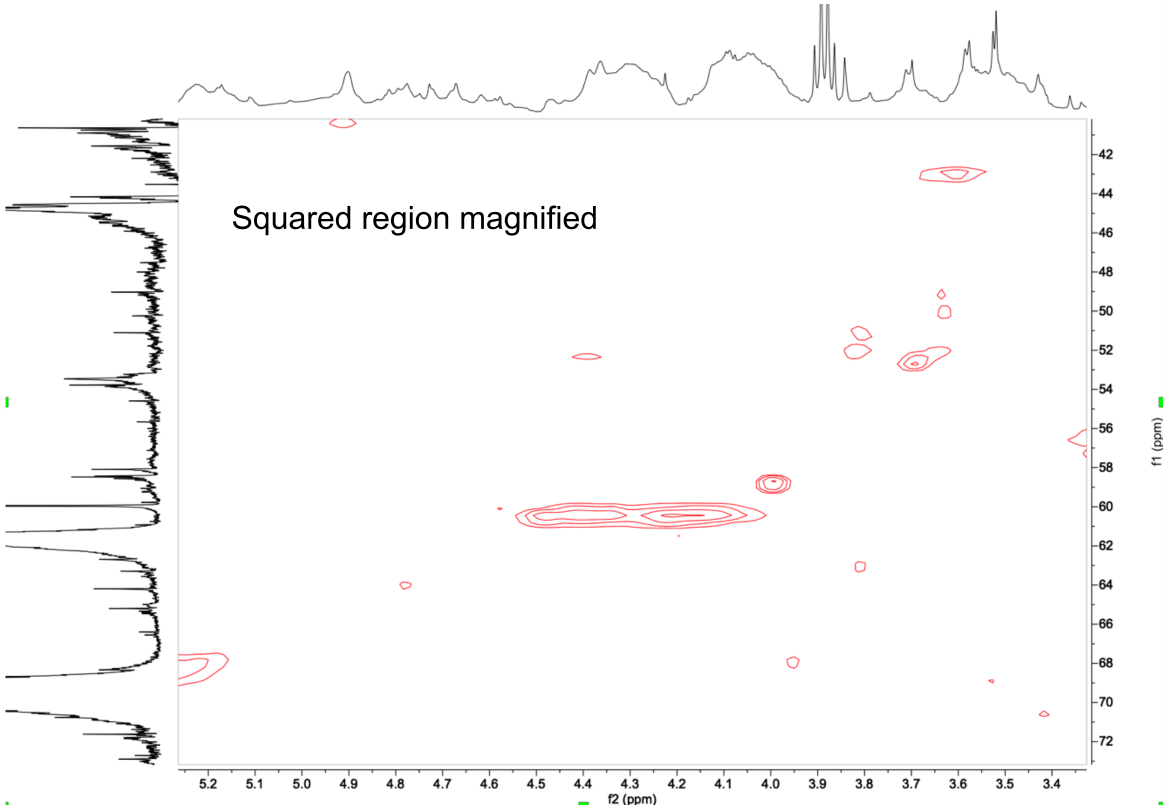

9. Crystallographic Data for Compound 2

General Materials and Methods

Single-crystal X-ray structure analyses were carried out on a Bruker D8 Venture equipped with dual monochromated radiation MoKa (λ = 0.71073 Å) and CuKα (λ = 1.54178). The single crystals were mounted on MicroMesh (MiTeGen) with paratone oil at 100 K under a nitrogen cryostream. Total of 6453 (using Mo) and 12880 (using Cu) for sclareolide. 0.5°-wide w- or f-scan frames with counting times of 1-30 seconds were collected with Bruker PHOTON III C14 detector. X-rays were provided by a four-circle diffractometer rotating anode operating at 50.0 kV and 1.40 mA (for Mo) and 1.10 mA (for Cu) and equipped with Helios multilayer X-ray optics. Preliminary lattice constants were obtained with the Bruker program APEX4. Integrated reflection intensities were produced using the Bruker program APEX4. The structures were determined by intrinsic phasing (SHELXT 2014/5) and refined by full- matrix least-squares refinement on F2 (SHELXL-2017/1) using the Olex2 software package using Least Squares minimization. The final structural model incorporated anisotropic thermal parameters for all non-hydrogen atoms and isotropic thermal parameters for all hydrogen atoms. All hydrogen atoms were located in a difference Fourier and initially included in the structural model as independent isotropic atoms whose parameters were allowed to refine in least-squares refinement cycles. The hydrogen atoms a bonded to carbon were eventually placed in calculated position and refine in the riding model sp^2- or sp^3-hybridized positions with C−H bond lengths of 0.95 - 0.99 Å.

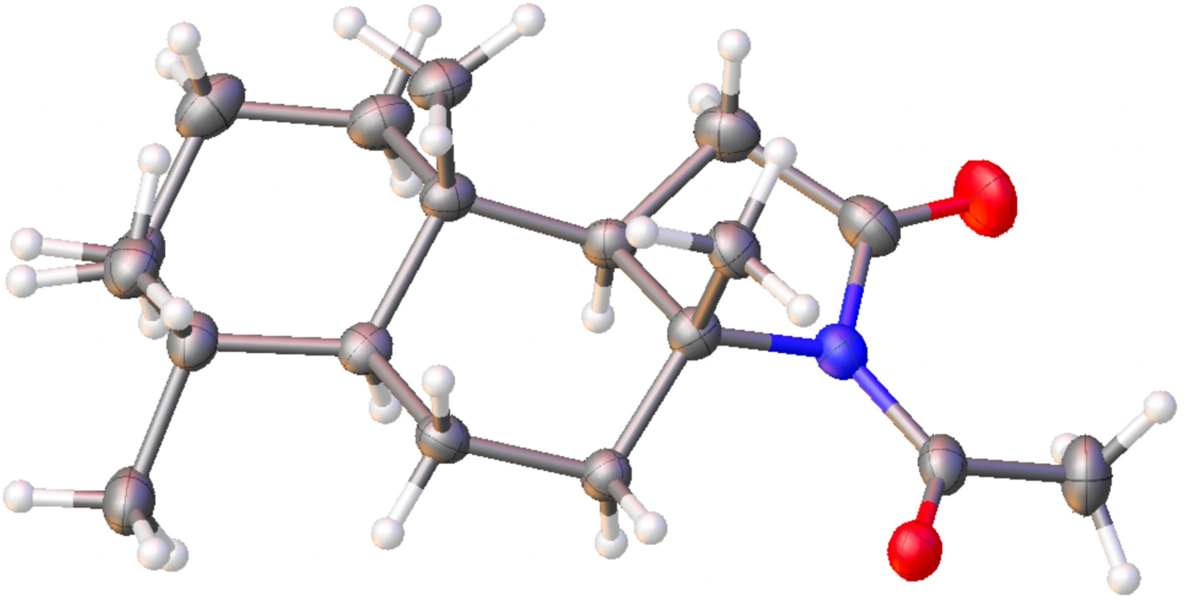

Table S6. Crystal data and structure refinement for mo_Sclareolide.

Identification code	mo_Sclareolide
Empirical formula	$C_{18}H_{29}NO_2$
Formula weight	291.476
Temperature/K	103.00
Crystal system	orthorhombic
Space group	$P2_12_12_1$
a/Å	7.1642(8)
b/Å	10.0219(10)
c/Å	22.705(3)
α/°	90
β/°	90
γ/°	90
Volume/Å^3	1630.2(3)
Z	4
ρ_{calc}g/cm^3	1.188
μ/mm^{-1}	0.076
F(000)	640.4
Crystal size/mm^3	0.1 × 0.1 × 0.05
Radiation	Mo Kα (λ = 0.71073)
2Θ range for data collection/°	4.44 to 50.7
Index ranges	-8 ≤ h ≤ 8, -12 ≤ k ≤ 11, -27 ≤ l ≤ 21
Reflections collected	6453
Independent reflections	2950 [R_{int} = 0.0714, R_{sigma} = 0.0992]
Data/restraints/parameters	2950/0/195
Goodness-of-fit on F^2	0.990
Final R indexes [I>=2σ (I)]	R_1 = 0.0530, wR_2 = 0.0997
Final R indexes [all data]	R_1 = 0.0853, wR_2 = 0.1137
Largest diff. peak/hole / e Å^{-3}	0.27/-0.28
Flack parameter	-0.5(18)

Table S7. Fractional Atomic Coordinates (×10[4]) and Equivalent Isotropic Displacement Parameters (Å^2×10^3) for mo_Sclareolide. U_{eq} is defined as 1/3 of the trace of the orthogonalised U_{IJ} tensor.

Atom	*x*	*y*	*z*	U(eq)
O1	2964(3)	7087.5(18)	7854.2(9)	30.0(5)
O2	7572(3)	5233(2)	8583.1(10)	49.2(7)
C7	5454(4)	4735(3)	5006.3(15)	35.9(8)
C3	7254(4)	5133(3)	5333.2(13)	27.4(7)
C4	6806(3)	5373(2)	5989.1(13)	23.1(6)
C5	8515(3)	5515(3)	6411.8(13)	23.6(6)
C12	7654(3)	5434(3)	7030.1(12)	23.4(6)
C11	6185(4)	6507(2)	7191.3(13)	24.0(6)
N1	5772(3)	6059(2)	7809.5(11)	26.0(6)
C15	4113(4)	6402(3)	8099.1(14)	27.6(7)
C16	3807(5)	5903(3)	8714.2(14)	40.3(8)
C14	7386(4)	5513(3)	8068.4(15)	32.9(7)
C13	8828(4)	5339(3)	7587.5(14)	32.4(7)
C6	9802(4)	4301(3)	6325.0(14)	32.0(7)
C1	10336(4)	4086(3)	5680.3(15)	36.8(8)
C2	8608(4)	3948(3)	5294.2(15)	33.9(7)
C8	8057(4)	6358(3)	5009.1(14)	32.2(7)
C9	5338(4)	6447(3)	6120.8(13)	25.5(7)
C10	4593(4)	6406(3)	6749.1(13)	23.6(7)
C17	6879(4)	7954(3)	7252.8(13)	27.9(7)
C18	9688(4)	6801(3)	6322.4(15)	30.4(8)

Table S8. Anisotropic Displacement Parameters (Å^2×10^3) for mo_Sclareolide. The Anisotropic displacement factor exponent takes the form: -2π^2[h^{2}a*^{2}U$_{11}$+2hka*b*U$_{12}$+…].

Atom	U_{11}	U_{22}	U_{33}	U_{12}	U_{13}	U_{23}
O1	29.9(10)	32.8(10)	27.4(13)	4.2(9)	0.9(10)	-3.5(9)
O2	51.1(13)	70.2(17)	26.2(16)	8.1(12)	-8.9(11)	9.7(12)
C7	41.4(16)	39.0(17)	27(2)	-4.8(15)	7.4(14)	-6.6(14)
C3	31.7(15)	25.0(15)	25.5(17)	-3.7(13)	5.0(13)	-2.5(13)
C4	24.7(13)	17.3(13)	27.3(17)	-2.6(12)	2.4(12)	0.2(11)
C5	21.1(13)	21.2(13)	28.5(18)	1.0(12)	1.1(12)	-1.3(12)
C12	21.6(12)	23.5(13)	25.2(17)	2.9(11)	-2.6(12)	2.3(12)
C11	24.7(14)	21.2(12)	26.0(18)	1.2(12)	0.6(13)	-0.1(12)
N1	28.8(13)	25.4(11)	23.6(15)	-0.7(10)	-2.7(11)	0.5(11)
C15	35.0(16)	25.6(14)	22.3(18)	-4.6(13)	1.3(14)	-4.9(12)
C16	54.4(19)	42.4(17)	24.2(19)	4.6(17)	4.2(16)	-3.2(14)
C14	35.3(16)	33.4(16)	30(2)	-0.4(14)	-8.6(15)	1.3(14)
C13	28.6(15)	34.2(16)	34(2)	3.9(14)	-6.2(14)	4.1(14)
C6	25.8(14)	30.6(16)	40(2)	2.7(13)	2.4(14)	0.8(14)
C1	33.6(16)	29.5(16)	47(2)	4.4(13)	12.8(16)	1.2(15)
C2	37.3(17)	30.6(16)	34(2)	-1.7(14)	13.9(15)	-3.0(14)
C8	33.8(15)	32.9(15)	29.9(19)	-4.1(14)	6.3(15)	0.7(14)
C9	23.2(13)	26.3(14)	26.9(18)	-0.6(12)	-2.8(13)	-3.0(13)
C10	23.4(14)	25.2(14)	22.3(17)	1.3(12)	0.4(12)	1.2(12)
C17	31.0(15)	26.4(14)	26.3(18)	-0.4(13)	-2.6(14)	-3.9(13)
C18	23.9(14)	30.3(15)	37(2)	-2.9(13)	2.6(14)	-0.1(13)

Table S9. Bond Lengths for mo_Sclareolide

Atom	Atom	Length/Å	Atom	Atom	Length/Å
O1	C15	1.208(3)	C12	C13	1.523(4)
O2	C14	1.209(4)	C11	N1	1.503(4)
C7	C3	1.540(4)	C11	C10	1.523(4)
C3	C4	1.542(4)	C11	C17	1.539(3)
C3	C2	1.536(4)	N1	C15	1.401(4)
C3	C8	1.542(4)	N1	C14	1.408(3)
C4	C5	1.562(4)	C15	C16	1.499(4)
C4	C9	1.535(3)	C14	C13	1.513(4)
C5	C12	1.536(4)	C6	C1	1.528(4)
C5	C6	1.539(4)	C1	C2	1.523(4)
C5	C18	1.552(4)	C9	C10	1.524(4)
C12	C11	1.549(4)			

Table S10. Bond Angles for mo_Sclareolide.

Atom	Atom	Atom	Angle/˚	Atom	Atom	Atom	Angle/˚
C4	C3	C7	109.4(2)	C10	C11	N1	116.6(2)
C2	C3	C7	107.5(2)	C17	C11	C12	117.1(2)
C2	C3	C4	107.9(2)	C17	C11	N1	105.1(2)
C8	C3	C7	106.8(2)	C17	C11	C10	111.4(2)
C8	C3	C4	114.5(2)	C15	N1	C11	122.2(2)
C8	C3	C2	110.6(2)	C14	N1	C11	110.1(2)
C5	C4	C3	116.4(2)	C14	N1	C15	126.6(3)
C9	C4	C3	116.1(2)	N1	C15	O1	120.1(3)
C9	C4	C5	110.7(2)	C16	C15	O1	121.2(3)
C12	C5	C4	104.0(2)	C16	C15	N1	118.7(3)
C6	C5	C4	108.6(2)	N1	C14	O2	125.7(3)
C6	C5	C12	108.4(2)	C13	C14	O2	126.6(3)
C18	C5	C4	114.8(2)	C13	C14	N1	107.7(3)
C18	C5	C12	112.4(2)	C14	C13	C12	102.4(2)
C18	C5	C6	108.4(2)	C1	C6	C5	112.6(2)
C11	C12	C5	116.9(2)	C2	C1	C6	111.1(2)
C13	C12	C5	122.7(2)	C1	C2	C3	114.2(2)
C13	C12	C11	102.9(2)	C10	C9	C4	113.8(2)
N1	C11	C12	98.5(2)	C9	C10	C11	110.7(2)
C10	C11	C12	107.8(2)				

Table S11. Torsion Angles for mo_Sclareolide.

A	B	C	D	Angle/˚	A	B	C	D	Angle/˚
O1	C15	N1	C11	-0.6(3)	C4	C5	C12	C13	171.51(18)
O1	C15	N1	C14	166.1(2)	C4	C5	C6	C1	53.0(2)
O2	C14	N1	C11	169.4(3)	C4	C9	C10	C11	55.9(2)
O2	C14	N1	C15	1.4(4)	C5	C12	C11	N1	-179.4(2)
O2	C14	C13	C12	163.1(3)	C5	C12	C11	C10	59.0(2)
C7	C3	C4	C5	168.6(2)	C5	C12	C11	C17	-67.5(3)
C7	C3	C4	C9	-58.4(2)	C5	C12	C13	C14	171.1(3)
C7	C3	C2	C1	-170.5(2)	C5	C6	C1	C2	-56.2(2)
C3	C4	C5	C12	-168.2(2)	C12	C11	N1	C15	-158.53(18)
C3	C4	C5	C6	-52.9(2)	C12	C11	N1	C14	32.9(2)
C3	C4	C5	C18	68.6(2)	C12	C11	C10	C9	-52.0(2)
C3	C4	C9	C10	165.4(2)	C12	C13	C14	N1	-16.8(2)
C3	C2	C1	C6	56.5(3)	C11	N1	C15	C16	179.5(2)
C4	C5	C12	C11	-59.9(2)	C11	N1	C14	C13	-10.8(2)

Table S12. Hydrogen Atom Coordinates (Å×10^4) and Isotropic Displacement Parameters (Å^2×10^3) for mo_Sclareolide.

**Atom**	***x***	***y***	***z***	**U(eq)**
H7a	5765(5)	4443(18)	4606(3)	53.8(11)
H7b	4839(14)	4005(13)	5218(5)	53.8(11)
H7c	4612(12)	5505(6)	4987(8)	53.8(11)
H4	6196(3)	4524(2)	6117.7(13)	27.7(8)
H12	6929(3)	4582(3)	7025.0(12)	28.1(7)
H16a	2465(5)	5870(17)	8797(3)	60.5(12)
H16b	4340(20)	5006(8)	8753(3)	60.5(12)
H16c	4420(20)	6506(10)	8994.2(15)	60.5(12)
H13a	9455(4)	4461(3)	7617.1(14)	38.9(9)
H13b	9780(4)	6054(3)	7601.9(14)	38.9(9)
H6a	10951(4)	4428(3)	6560.3(14)	38.4(9)
H6b	9163(4)	3492(3)	6473.5(14)	38.4(9)
H1a	11092(4)	4851(3)	5541.8(15)	44.2(10)
H1b	11106(4)	3270(3)	5645.5(15)	44.2(10)
H2a	7934(4)	3125(3)	5408.8(15)	40.6(9)
H2b	9009(4)	3842(3)	4879.6(15)	40.6(9)
H8a	8120(20)	6171(7)	4585.8(17)	48.3(11)
H8b	7249(15)	7131(5)	5078(7)	48.3(11)
H8c	9313(11)	6548(11)	5158(6)	48.3(11)
H9a	5896(4)	7336(3)	6048.0(13)	30.6(8)
H9b	4281(4)	6337(3)	5844.4(13)	30.6(8)
H10a	3906(4)	5561(3)	6812.5(13)	28.4(8)
H10b	3713(4)	7154(3)	6810.3(13)	28.4(8)
H17a	7190(20)	8307(6)	6862.9(16)	41.8(10)
H17b	5895(10)	8502(5)	7430(7)	41.8(10)
H17c	7989(16)	7972(3)	7505(7)	41.8(10)
H18a	10650(18)	6638(6)	6024(6)	45.6(11)
H18b	8872(6)	7527(6)	6191(8)	45.6(11)
H18c	10280(20)	7049(11)	6695(2)	45.6(11)

Table S13. Crystal data and structure refinement for Cu_Sclareolide.

Identification code	Cu_Sclareolide
Empirical formula	$C_{18}H_{29}NO_2$
Formula weight	291.436
Temperature/K	100.00
Crystal system	orthorhombic
Space group	$P2_12_12_1$
a/Å	7.1680(3)
b/Å	10.0255(4)
c/Å	22.7079(9)
α/°	90
β/°	90
γ/°	90
Volume/Å^3	1631.85(11)
Z	4
ρ_{calc}g/cm^3	1.186
μ/mm^{-1}	0.593
F(000)	641.8
Crystal size/mm^3	0.1 × 0.1 × 0.05
Radiation	Cu Kα (λ = 1.54178)
2Θ range for data collection/°	9.64 to 100.86
Index ranges	-7 ≤ h ≤ 7, -10 ≤ k ≤ 10, -22 ≤ l ≤ 22
Reflections collected	12880
Independent reflections	1681 [R_{int} = 0.0436, R_{sigma} = 0.0287]
Data/restraints/parameters	1681/0/195
Goodness-of-fit on F^2	1.098
Final R indexes [I>=2σ (I)]	R_1 = 0.0216, wR_2 = 0.0559
Final R indexes [all data]	R_1 = 0.0253, wR_2 = 0.0566
Largest diff. peak/hole / e Å^{-3}	0.13/-0.17
Flack parameter	-0.06(19)

Table S14. Fractional Atomic Coordinates (×10^4) and Equivalent Isotropic Displacement Parameters ($Å^2$×10^3) for Cu_Sclareolide. U_{eq} is defined as 1/3 of of the trace of the orthogonalised U_{IJ} tensor.

**Atom**	***x***	***y***	***z***	**U(eq)**
O1	2966.2(15)	2913.4(11)	2146.6(5)	29.1(3)
O2	7572.8(17)	4761.6(15)	1417.3(5)	49.0(4)
N1	5773.1(16)	3939.4(12)	2189.3(5)	23.5(3)
C7	5452(2)	5266.7(18)	4993.1(7)	33.3(5)
C1	7246(2)	4865.3(17)	4669.1(7)	26.5(4)
C6	6808.5(19)	4630.7(15)	4009.2(7)	21.6(4)
C5	8526(2)	4477.3(16)	3587.6(7)	23.9(4)
C12	7660.2(19)	4559.7(16)	2969.3(6)	21.4(4)
C11	6192(2)	3491.6(14)	2809.8(7)	21.1(4)
C16	4123(2)	3594.9(16)	1899.2(7)	25.2(4)
C17	3814(3)	4096.6(19)	1285.6(7)	38.9(5)
C14	7389(2)	4484.4(17)	1933.8(8)	31.6(4)
C13	8828(2)	4659.4(17)	2411.6(7)	30.8(4)
C8	8049(2)	3640.9(17)	4988.7(7)	31.1(4)
C2	8606(2)	6044.1(17)	4708.9(7)	32.5(4)
C3	10327(2)	5916.6(18)	4319.7(8)	35.9(4)
C4	9802(2)	5694.9(18)	3676.3(7)	30.5(4)
C9	5343.2(19)	3554.7(15)	3882.2(7)	21.9(4)
C10	4599.1(19)	3593.0(16)	3248.7(6)	21.3(4)
C15	6876(2)	2050.6(16)	2751.8(7)	27.2(4)
C18	9675(2)	3200.2(18)	3676.1(7)	28.4(4)

Table S15. Anisotropic Displacement Parameters (Å^2×10^3) for Cu_Sclareolide. The Anisotropic displacement factor exponent takes the form: -2π^2[h^{2}a*^{2}U$_{11}$+2hka*b*U$_{12}$+…].

Atom	U_{11}	U_{22}	U_{33}	U_{12}	U_{13}	U_{23}
O1	29.3(6)	30.9(6)	27.1(6)	-3.2(6)	-2.8(5)	-3.1(5)
O2	50.8(7)	69.9(10)	26.5(8)	-10.2(7)	10.3(6)	10.5(7)
N1	24.8(7)	23.0(7)	22.7(7)	-0.0(6)	2.9(6)	0.3(6)
C7	38.7(9)	38.3(11)	22.8(9)	7.6(8)	-5.8(7)	-7.3(8)
C1	28.6(8)	25.2(10)	25.6(9)	2.2(8)	-8.1(7)	-1.6(7)
C6	19.5(7)	18.2(9)	27.0(9)	3.8(7)	-2.7(6)	0.8(7)
C5	17.7(8)	22.7(9)	31.4(9)	-1.3(8)	-1.2(6)	1.7(7)
C12	16.4(7)	21.0(9)	26.8(9)	-1.3(7)	3.0(7)	2.2(7)
C11	21.5(8)	19.6(8)	22.3(9)	0.2(8)	1.1(7)	1.1(7)
C16	32.5(10)	20.4(9)	22.6(9)	3.9(8)	1.6(8)	-6.9(7)
C17	50.9(10)	39.3(11)	26.6(10)	-0.0(10)	-4.5(8)	-3.6(8)
C14	33.2(9)	29.6(10)	32.1(11)	-0.3(8)	9.8(8)	1.3(8)
C13	25.7(8)	32.3(10)	34.5(10)	-3.5(9)	5.8(7)	3.6(8)
C8	33.4(8)	33.2(10)	26.9(9)	4.1(8)	-5.1(7)	0.8(7)
C2	37.1(9)	29.0(10)	31.4(9)	3.1(8)	-15.5(7)	-3.2(8)
C3	31.5(8)	29.0(10)	47.1(11)	-5.1(8)	-16.6(8)	2.5(9)
C4	20.0(8)	28.6(10)	42.8(11)	-2.4(9)	-4.0(7)	3.5(8)
C9	19.3(7)	25.4(9)	21.0(8)	2.5(7)	2.4(7)	-0.9(7)
C10	16.8(7)	22.6(9)	24.6(9)	-2.3(7)	-0.9(7)	-2.2(7)
C15	28.1(9)	23.9(9)	29.6(9)	0.4(7)	1.4(7)	-3.6(7)
C18	19.5(8)	29.5(10)	36.3(10)	2.7(8)	-1.3(7)	1.1(8)

Table S16. Bond Lengths for Cu_Sclareolide.

Atom	Atom	Length/Å	Atom	Atom	Length/Å
O1	C16	1.2127(19)	C5	C4	1.539(2)
O2	C14	1.212(2)	C5	C18	1.536(2)
N1	C11	1.509(2)	C12	C11	1.544(2)
N1	C16	1.397(2)	C12	C13	1.521(2)
N1	C14	1.406(2)	C11	C10	1.519(2)
C7	C1	1.535(2)	C11	C15	1.531(2)
C1	C6	1.549(2)	C16	C17	1.498(2)
C1	C8	1.538(2)	C14	C13	1.507(2)
C1	C2	1.535(2)	C2	C3	1.522(2)
C6	C5	1.567(2)	C3	C4	1.525(2)
C6	C9	1.533(2)	C9	C10	1.535(2)
C5	C12	1.537(2)			

Table S17. Bond Angles for Cu_Sclareolide.

Atom	Atom	Atom	Angle/˚	Atom	Atom	Atom	Angle/˚
C16	N1	C11	122.37(12)	C13	C12	C11	103.02(12)
C14	N1	C11	109.68(12)	C12	C11	N1	98.53(11)
C14	N1	C16	126.78(13)	C10	C11	N1	116.29(11)
C6	C1	C7	109.50(12)	C10	C11	C12	108.17(12)
C8	C1	C7	107.26(13)	C15	C11	N1	105.31(12)
C8	C1	C6	114.27(13)	C15	C11	C12	117.17(12)
C2	C1	C7	107.60(13)	C15	C11	C10	111.10(12)
C2	C1	C6	107.60(12)	N1	C16	O1	119.99(14)
C2	C1	C8	110.42(12)	C17	C16	O1	121.26(15)
C5	C6	C1	116.56(11)	C17	C16	N1	118.75(15)
C9	C6	C1	115.32(12)	N1	C14	O2	125.28(15)
C9	C6	C5	110.73(12)	C13	C14	O2	126.55(14)
C12	C5	C6	103.64(11)	C13	C14	N1	108.17(13)
C4	C5	C6	108.00(12)	C14	C13	C12	102.42(12)
C4	C5	C12	108.47(12)	C3	C2	C1	114.58(13)
C18	C5	C6	115.04(13)	C4	C3	C2	111.62(12)
C18	C5	C12	112.39(13)	C3	C4	C5	112.81(13)
C18	C5	C4	109.00(12)	C10	C9	C6	113.39(12)
C11	C12	C5	116.86(12)	C9	C10	C11	110.61(11)
C13	C12	C5	122.80(12)				

Table S18. Torsion Angles for Cu_Sclareolide.

A	B	C	D	Angle/˚	A	B	C	D	Angle/˚
O1	C16	N1	C11	0.08(17)	C1	C6	C5	C4	-53.53(14)
O1	C16	N1	C14	166.44(14)	C1	C6	C5	C18	68.44(15)
O2	C14	N1	C11	169.29(17)	C1	C6	C9	C10	165.60(12)
O2	C14	N1	C16	1.5(2)	C1	C2	C3	C4	55.53(14)
O2	C14	C13	C12	163.39(19)	C6	C5	C12	C11	-60.24(12)
N1	C11	C12	C5	-179.36(9)	C6	C5	C12	C13	170.95(11)
N1	C11	C12	C13	-41.60(11)	C6	C5	C4	C3	53.15(13)
N1	C11	C10	C9	-161.53(12)	C6	C9	C10	C11	55.69(13)
N1	C14	C13	C12	-16.55(14)	C5	C12	C11	C10	59.27(14)
C7	C1	C6	C5	168.85(12)	C5	C12	C11	C15	-67.20(14)
C7	C1	C6	C9	-58.75(15)	C5	C12	C13	C14	171.06(15)
C7	C1	C2	C3	-169.70(12)	C5	C4	C3	C2	-55.79(14)
C1	C6	C5	C12	-168.47(14)	C12	C11	C10	C9	-51.88(12)

Table S19. Hydrogen Atom Coordinates (Å×10^4) and Isotropic Displacement Parameters (Å^2×10^3) for Cu_Sclareolide.

Atom	*x*	*y*	*z*	U(eq)
H7a	4835(8)	5991(7)	4778(3)	49.9(7)
H7b	4613(7)	4497(3)	5016(4)	49.9(7)
H7c	5762(3)	5569(10)	5391.7(18)	49.9(7)
H6	6201.5(19)	5479.1(15)	3879.1(7)	25.9(4)
H12	6935.5(19)	5412.0(16)	2975.8(6)	25.7(4)
H17a	4454(13)	3511(7)	1005.4(8)	58.4(7)
H17b	2475(3)	4104(11)	1199.4(19)	58.4(7)
H17c	4312(14)	5004(5)	1250.5(15)	58.4(7)
H13a	9451(2)	5538.3(17)	2382.0(7)	37.0(5)
H13b	9783(2)	3946.6(17)	2395.9(7)	37.0(5)
H8a	7255(8)	2864(3)	4912(4)	46.7(6)
H8b	9314(6)	3463(6)	4844(3)	46.7(6)
H8c	8090(14)	3815(4)	5413.2(8)	46.7(6)
H2a	7933(2)	6869.8(17)	4599.3(7)	39.0(5)
H2b	9016(2)	6140.1(17)	5123.0(7)	39.0(5)
H3a	11085(2)	6738.2(18)	4353.2(8)	43.0(5)
H3b	11095(2)	5158.8(18)	4458.5(8)	43.0(5)
H4a	10954(2)	5570.5(18)	3442.6(7)	36.6(5)
H4b	9160(2)	6500.5(18)	3526.0(7)	36.6(5)
H9a	5900.1(19)	2667.2(15)	3957.1(7)	26.3(4)
H9b	4285.5(19)	3669.5(15)	4157.9(7)	26.3(4)
H10a	3910.5(19)	4436.3(16)	3183.8(6)	25.6(5)
H10b	3721.5(19)	2843.4(16)	3187.2(6)	25.6(5)
H15a	5921(6)	1515(3)	2553(4)	40.8(6)
H15b	8030(8)	2032.7(19)	2521(4)	40.8(6)
H15c	7114(13)	1682(4)	3144.3(7)	40.8(6)
H18a	8853(3)	2478(3)	3808(4)	42.7(6)
H18b	10266(11)	2949(6)	3303.1(13)	42.7(6)
H18c	10639(9)	3359(3)	3974(3)	42.7(6)

10. Computations

Electrooxidation of flavin quinone yields a flavinium salt with five possible resonance and/or tautomeric states. Therefore, to determine the thermicity of proton transfer from the ground- or excited-state of these species, we considered an average of all five forms. The average energies of the five individual ground- and excited-state isodesmic reactions were used to determine the favorability of proton transfer in the case of lactone **3**.

Lactone **3** Neutral

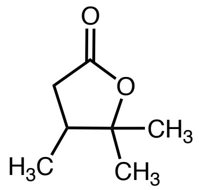

SPARTAN '18 Quantum Mechanics Program: (x86/Darwin) build 1.4.0

Job type: Single point.
Method: RB3LYP
Basis set: 6-311+G**
Number of basis functions: 270
Number of electrons: 70

SCF model:
A restricted hybrid HF-DFT SCF calculation will be
performed using Pulay DIIS + Geometric Direct Minimization

SCF total energy: -424.5679806 hartrees

Reason for exit: Successful completion
Quantum Calculation CPU Time : 1:26.18
Quantum Calculation Wall Time: 1:37.52

SPARTAN '18 Properties Program: (x86/Darwin) build 1.4.0

Reason for exit: Successful completion
Properties CPU Time : .71
Properties Wall Time: 1.73

Lactone **3** Protonated

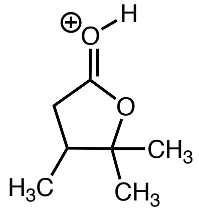

SPARTAN '18 Quantum Mechanics Program: (x86/Darwin) build 1.4.0

Job type: Single point.
Method: RB3LYP
Basis set: 6-311+G**
Number of basis functions: 276
Charge : +1
Number of electrons: 70
Parallel Job: 4 threads

SCF model:
A restricted hybrid HF-DFT SCF calculation will be
performed using Pulay DIIS + Geometric Direct Minimization

SCF total energy: -424.9088547 hartrees

Reason for exit: Successful completion
Quantum Calculation CPU Time : 1:23.63
Quantum Calculation Wall Time: 26.41

SPARTAN '18 Properties Program: (x86/Darwin) build 1.4.0

Reason for exit: Successful completion
Properties CPU Time : .56
Properties Wall Time: .55

N3 Flavinium Ground State

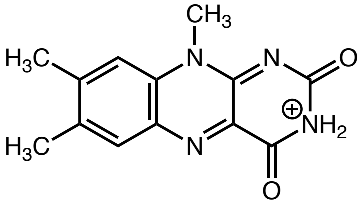

SPARTAN '18 Quantum Mechanics Program: (x86/Darwin) build 1.4.0

Job type: Single point.
Method: RB3LYP
Basis set: 6-311+G**
Number of basis functions: 496
Charge : +1
Number of electrons: 134

SCF model:
A restricted hybrid HF-DFT SCF calculation will be
performed using Pulay DIIS + Geometric Direct Minimization

SCF total energy: -872.7115730 hartrees

Reason for exit: Successful completion
Quantum Calculation CPU Time : 6:38.79
Quantum Calculation Wall Time: 7:06.67

SPARTAN '18 Properties Program: (x86/Darwin) build 1.4.0

Reason for exit: Successful completion
Properties CPU Time : 2.54
Properties Wall Time: 3.67

N3 Flavinium Excited State

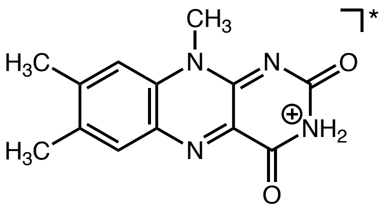

SPARTAN '18 Quantum Mechanics Program: (x86/Darwin) build 1.4.0

WARNING: Optimizations and density not supported for this method.
No wavefunction will be generated

Job type: Single point.
Excited States: 2
Method: RMP2(FC)
Basis set: 6-311+G**
Number of basis functions: 496
Charge : +1
Number of electrons: 134

SCF model:
A restricted Hartree-Fock SCF calculation will be
performed using Pulay DIIS + Geometric Direct Minimization

SCF total energy: -867.3577571 hartrees
CIS Energy: -867.2251161 First Excited State (singlet)

Correlation model:
An MP2 calculation will be performed

Reason for exit: Successful completion
Quantum Calculation CPU Time : 1:38:34.21
Quantum Calculation Wall Time: 3:30:06.29

SPARTAN '18 Properties Program: (x86/Darwin) build 1.4.0
Properties for excited states not implemented for RMP2(FC)
...Wavefunction properties ignored

Reason for exit: Successful completion
Properties CPU Time : .11
Properties Wall Time: 2.30

N10a Flavinium Ground State

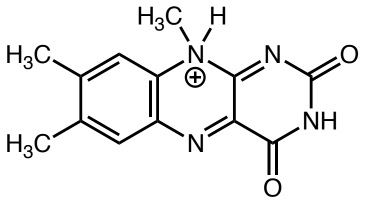

SPARTAN '18 Quantum Mechanics Program: (x86/Darwin) build 1.4.0

Job type: Single point.
Method: RB3LYP
Basis set: 6-311+G**
Number of basis functions: 496
Charge : +1
Number of electrons: 134

SCF model:
A restricted hybrid HF-DFT SCF calculation will be
performed using Pulay DIIS + Geometric Direct Minimization

SCF total energy: -872.6450711 hartrees

Reason for exit: Successful completion
Quantum Calculation CPU Time : 8:01.05
Quantum Calculation Wall Time: 8:32.83

SPARTAN '18 Properties Program: (x86/Darwin) build 1.4.0

Reason for exit: Successful completion
Properties CPU Time : 2.97
Properties Wall Time: 4.13

N10a Flavinium Excited State

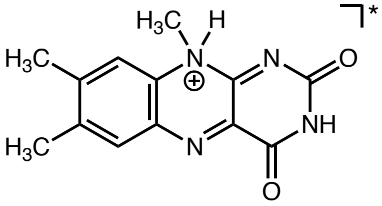

SPARTAN '18 Quantum Mechanics Program: (x86/Darwin) build 1.4.0

WARNING: Optimizations and density not supported for this method.
No wavefunction will be generated

Job type: Single point.
Excited States: 2
Method: RMP2(FC)
Basis set: 6-311+G**
Number of basis functions: 496
Charge : +1
Number of electrons: 134

SCF model:
A restricted Hartree-Fock SCF calculation will be
performed using Pulay DIIS + Geometric Direct Minimization

SCF total energy: -867.3370630 hartrees
CIS Energy: -867.2183282 First Excited State (singlet)

Correlation model:
An MP2 calculation will be performed

Reason for exit: Successful completion
Quantum Calculation CPU Time : 1:42:56.57
Quantum Calculation Wall Time: 3:34:41.99

SPARTAN '18 Properties Program: (x86/Darwin) build 1.4.0
Properties for excited states not implemented for RMP2(FC)
...Wavefunction properties ignored

Reason for exit: Successful completion
Properties CPU Time : .12
Properties Wall Time: .16

N1 Flavinium Ground State

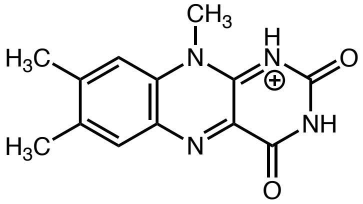

SPARTAN '18 Quantum Mechanics Program: (x86/Darwin) build 1.4.0

Job type: Single point.
Method: RB3LYP
Basis set: 6-311+G**
Number of basis functions: 496
Charge : +1
Number of electrons: 134
Parallel Job: 4 threads

SCF model:
A restricted hybrid HF-DFT SCF calculation will be
performed using Pulay DIIS + Geometric Direct Minimization

SCF total energy: -872.7041066 hartrees

Reason for exit: Successful completion
Quantum Calculation CPU Time : 7:26.29
Quantum Calculation Wall Time: 2:13.54

SPARTAN '18 Properties Program: (x86/Darwin) build 1.4.0

Reason for exit: Successful completion
Properties CPU Time : 2.23
Properties Wall Time: 3.42

N1 Flavinium Excited State

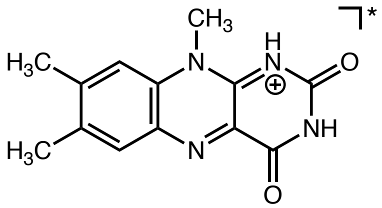

SPARTAN '18 Quantum Mechanics Program: (x86/Darwin) build 1.4.0

WARNING: Optimizations and density not supported for this method.
No wavefunction will be generated

Job type: Single point.
Excited States: 2
Method: RMP2(FC)
Basis set: 6-311+G**
Number of basis functions: 496
Charge : +1
Number of electrons: 134

SCF model:
A restricted Hartree-Fock SCF calculation will be
performed using Pulay DIIS + Geometric Direct Minimization

SCF total energy: -867.3871844 hartrees
CIS Energy: -867.2832555 First Excited State (singlet)

Correlation model:
An MP2 calculation will be performed

Reason for exit: Successful completion
Quantum Calculation CPU Time : 1:05:30.21
Quantum Calculation Wall Time: 1:08:06.99

SPARTAN '18 Properties Program: (x86/Darwin) build 1.4.0
Properties for excited states not implemented for RMP2(FC)
...Wavefunction properties ignored

Reason for exit: Successful completion
Properties CPU Time : .10
Properties Wall Time: .16

N5 Flavinium Ground State

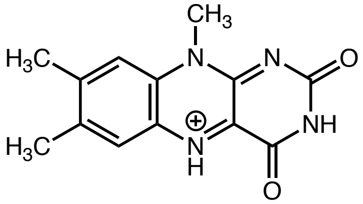

SPARTAN '18 Quantum Mechanics Program: (x86/Darwin) build 1.4.0

Job type: Single point.
Method: RB3LYP
Basis set: 6-311+G**
Number of basis functions: 496
Charge : +1
Number of electrons: 134

SCF model:
A restricted hybrid HF-DFT SCF calculation will be
performed using Pulay DIIS + Geometric Direct Minimization

SCF total energy: -872.7184894 hartrees

Reason for exit: Successful completion
Quantum Calculation CPU Time : 7:48.29
Quantum Calculation Wall Time: 8:19.37

SPARTAN '18 Properties Program: (x86/Darwin) build 1.4.0

Reason for exit: Successful completion
Properties CPU Time : 2.90
Properties Wall Time: 4.04

N5 Flavinium Excited State

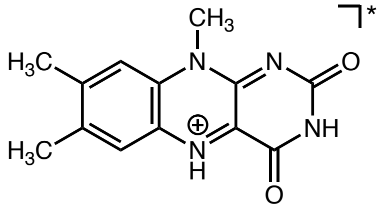

SPARTAN '18 Quantum Mechanics Program: (x86/Darwin) build 1.4.0

WARNING: Optimizations and density not supported for this method.
No wavefunction will be generated

Job type: Single point.
Excited States: 2
Method: RMP2(FC)
Basis set: 6-311+G**
Number of basis functions: 496
Charge : +1
Number of electrons: 134

SCF model:
A restricted Hartree-Fock SCF calculation will be
performed using Pulay DIIS + Geometric Direct Minimization

SCF total energy: -867.4201076 hartrees
CIS Energy: -867.2716317 First Excited State (singlet)

Correlation model:
An MP2 calculation will be performed

Reason for exit: Successful completion
Quantum Calculation CPU Time : 1:39:26.65
Quantum Calculation Wall Time: 3:29:48.08

SPARTAN '18 Properties Program: (x86/Darwin) build 1.4.0
Properties for excited states not implemented for RMP2(FC)
...Wavefunction properties ignored

Reason for exit: Successful completion
Properties CPU Time : .12
Properties Wall Time: 2.31

N10b Flavinium Ground State

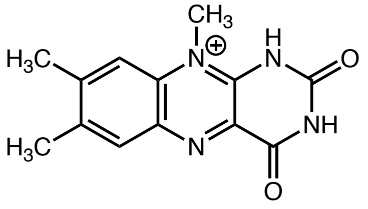

SPARTAN '18 Quantum Mechanics Program: (x86/Darwin) build 1.4.4

Job type: Single point.
Method: RB3LYP
Basis set: 6-311+G**
Number of basis functions: 496
Charge : +1
Number of electrons: 134
Parallel Job: 4 threads

SCF model:
A restricted hybrid HF-DFT SCF calculation will be
performed using Pulay DIIS + Geometric Direct Minimization

SCF total energy: -872.7185159 hartrees

Reason for exit: Successful completion
Quantum Calculation CPU Time : 6:43.60
Quantum Calculation Wall Time: 1:47.48

SPARTAN '18 Properties Program: (x86/Darwin) build 1.4.4

Reason for exit: Successful completion
Properties CPU Time : 2.23
Properties Wall Time: 3.14

N10b Flavinium Excited State

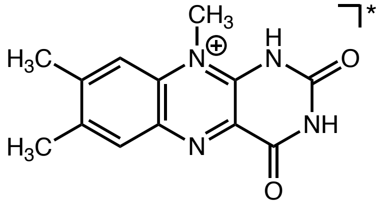

SPARTAN '18 Quantum Mechanics Program: (x86/Darwin) build 1.4.0

WARNING: Optimizations and density not supported for this method.
No wavefunction will be generated

Job type: Single point.
Excited States: 2
Method: RMP2(FC)
Basis set: 6-311+G**
Number of basis functions: 496
Charge : +1
Number of electrons: 134

SCF model:
A restricted Hartree-Fock SCF calculation will be
performed using Pulay DIIS + Geometric Direct Minimization

SCF total energy: -867.4206009 hartrees
CIS Energy: -867.2715451 First Excited State (singlet)

Correlation model:
An MP2 calculation will be performed

Reason for exit: Successful completion
Quantum Calculation CPU Time : 1:22:37.41
Quantum Calculation Wall Time: 1:52:02.29

SPARTAN '18 Properties Program: (x86/Darwin) build 1.4.0
Properties for excited states not implemented for RMP2(FC)
...Wavefunction properties ignored

Reason for exit: Successful completion
Properties CPU Time : .10
Properties Wall Time: 1.28

Lumiflavin Ground State

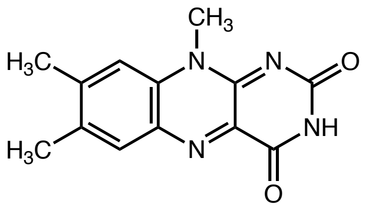

SPARTAN '18 Quantum Mechanics Program: (x86/Darwin) build 1.4.0

Job type: Single point.
Method: RB3LYP
Basis set: 6-311+G**
Number of basis functions: 490
Number of electrons: 134
Parallel Job: 4 threads

SCF model:
A restricted hybrid HF-DFT SCF calculation will be
performed using Pulay DIIS + Geometric Direct Minimization

SCF total energy: -872.3461190 hartrees

Reason for exit: Successful completion
Quantum Calculation CPU Time : 6:52.87
Quantum Calculation Wall Time: 1:56.99

SPARTAN '18 Properties Program: (x86/Darwin) build 1.4.0

Reason for exit: Successful completion
Properties CPU Time : 2.15
Properties Wall Time: 3.23

Lumiflavin Excited State

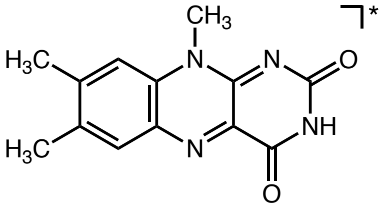

SPARTAN '18 Quantum Mechanics Program: (x86/Darwin) build 1.4.0

WARNING: Optimizations and density not supported for this method.
No wavefunction will be generated

Job type: Single point.
Excited States: 2
Method: RMP2(FC)
Basis set: 6-311+G**
Number of basis functions: 490
Number of electrons: 134

SCF model:
A restricted Hartree-Fock SCF calculation will be
performed using Pulay DIIS + Geometric Direct Minimization

SCF total energy: -867.0400524 hartrees
CIS Energy: -866.9013792 First Excited State (singlet)

Correlation model:
An MP2 calculation will be performed

Reason for exit: Successful completion
Quantum Calculation CPU Time : 1:26:30.09
Quantum Calculation Wall Time: 1:55:52.68

SPARTAN '18 Properties Program: (x86/Darwin) build 1.4.0
Properties for excited states not implemented for RMP2(FC)
...Wavefunction properties ignored

Reason for exit: Successful completion
Properties CPU Time : .10
Properties Wall Time: .10

11. References

1. National Institute for Occupational Safety and Health (NIOSH), NIOSH pocket guide to chemical hazards-Kerosene. URL: https://www.cdc.gov/niosh/npg/npgd0366.html

2. Goia, S.; Turner, M. A. P.; Woolley, J. M.; Horbury, M. D.; Borrill, A. J.; Tully, J. J.; Cobb, S. J.; Staniforth, M.; Hine, N. D. M.; Burriss, A.; Macpherson, J. V.; Robinson, B. R.; Stavros, V. G. Ultrafast transient absorption spectroelectrochemistry: femtosecond to nanosecond excited-state relaxation dynamics of the individual components of an anthraquinone redox couple. *Chem. Sci.*, **2022**, *13*, 486-496.

3. Woolley, J. M.; Staniforth, M.; Horbury, M. D.; Richings, G. W.; Wills, M.; Stavros, V. G.Unravelling the photoprotection properties of mycosporine amino acid motifs. *J. Phys. Chem. Lett.*, **2018**, *9*, 3042-3048.

4. Satoh, A. Y.; Trosko, J. E.; Masten, S. J. Methylene blue dye test for rapid qualitative detection of hydroxyl radicals formed in a Fenton's reaction aqueous solution. *Environ. Sci. Technol.*, **2007**, *41*, 2881-2887.

5. Wang, Z.; Zhao, H.; Qi, H.; Liu, X.; Liu, Y. Free radical behaviours during methylene blue degradation in the Fe^{2+}/H_2O_2 system. *Environ. Technol.*, **2019**, *40*, 1138-1145.

6. Mullen, K. M.; Van Stokkum, I. H. M. TIMP: An R package for modeling multi-way spectroscopic measurements. *J. Stat. Softw.*, **2007**, *18*, 1–46.

7. Snellenburg, J.; Laptenok, S.; Seger, R.; Mullen, K.; Van Stokkum, I. Glotaran: A Java-based graphical user interface for the R package TIMP. *J. Stat. Softw*. **2012**, *49*, 1–22.

8. Meng, Q.-Y.; Wang, S.; König, B. Carboxylation of aromatic and aliphatic bromides and triflates with CO2 by dual visible-light-nickel catalysis. *Angew. Chem. Int. Ed.*, **2017**, *56*, 13426-13430.

9. Nandy, S.; Das, A. K.; Bhar, S. Chemoselective formation of C–N bond in wet acetonitrile using amberlyst®-15(H) as a recyclable catalyst. *Synth. Commun.*, **2020**, *21*, 3326-3336.

10. De Luca, L.; Giacomelli, G.; Porcheddu, A. Beckmann rearrangement of oximes under very mild conditions. *J. Org. Chem.*, **2002**, *67*, 6272-6274.

11. Michaudel, Q.; Thevenet, D.; Baran, P. S. Intermolecular ritter-type C-H amination of

unactivated sp3 carbons. *J. Am. Chem. Soc*., **2012**, *134*, 2547-2550.

12. Nair, V.; Suja, T. D.; Mohanan, K. A. A convenient protocol for C-H oxidation mediated by an azido radical culminating in ritter-type amidation. *Tetrahedron Lett*., **2005**, *46*, 3217-3219.

13. Khusnutdinov, R. I.; Oshnyakova, T. M. Amidation of norbornene with organic nitriles in the presence of water catalyzed by iron compounds. *Tetrahedron Lett*., **2015**, *56*, 6368-6369.

14. Athavale, S. V.; Gao, S.; Das, A.; Mallojjala, S. C.; Alfonz, E.; Long, Y.; Hirschi, J. S.; Arnold, F. H. Enzymatic nitrogen insertion into unactivated C-H bonds. *J. Am. Chem. Soc.*, **2022**, *144*,19097-19105.

15. Zhang, L.; Chen, B.; He, P.; Li, G.; Zhang, L.-C.; Gao, S. Polyoxometalate-ionic liquid catalyzed ritter reaction for efficient synthesis of amides. *SynLett*, **2022**, *33*, 1515-1518.

16. Motokura, K.; Nakagiri, N.; Mori, K.; Mizugaki, T.; Ebitani, K.; Jitsukawa, K.; Kaneda, K. Efficient C-N bond formations catalyzed by a proton-exchanged montmorillonite as a heterogeneous Brønsted acid. *Org. Lett*., **2006**, *8*, 4617-4620.
